# Pervasive Inter-Individual Variation in Allele-Specific Expression in Monozygotic Twins

**DOI:** 10.3389/fgene.2019.01178

**Published:** 2019-11-26

**Authors:** Ronaldo da Silva Francisco Junior, Cristina dos Santos Ferreira, Juan Carlo Santos e Silva, Douglas Terra Machado, Yasmmin Côrtes Martins, Victor Ramos, Gustavo Simões Carnivali, Ana Beatriz Garcia, Enrique Medina-Acosta

**Affiliations:** ^1^Laboratório de Bioinformática, Laboratório Nacional de Computação Científica, Petrópolis, Brazil; ^2^Laboratório de Biotecnologia, Núcleo de Diagnóstico e Investigação Molecular, Universidade Estadual do Norte Fluminense, Campos dos Goytacazes, Brazil; ^3^Department of Genetics, Faculdade de Medicina de Ribeirão Preto, Universidade de São Paulo, Ribeirão Preto, Brazil; ^4^Department of Computational Science, Universidade Federal de Minas Gerais, Belo Horizonte, Brazil

**Keywords:** allele-specific expression, allele imbalance, Down syndrome, genomic imprinting, heterokaryotypic monozygotic co-twins, mitochondrial heteroplasmy, random monoallelic expression, trisomy 21

## Abstract

Despite being developed from one zygote, heterokaryotypic monozygotic (MZ) co-twins exhibit discordant karyotypes. Epigenomic studies in biological samples from heterokaryotypic MZ co-twins are of the most significant value for assessing the effects on gene- and allele-specific expression of an extranumerary chromosomal copy or structural chromosomal disparities in otherwise nearly identical germline genetic contributions. Here, we use RNA-Seq data from existing repositories to establish within-pair correlations for the breadth and magnitude of allele-specific expression (ASE) in heterokaryotypic MZ co-twins discordant for trisomy 21 and maternal 21q inheritance, as well as homokaryotypic co-twins. We show that there is a genome-wide disparity at ASE sites between the heterokaryotypic MZ co-twins. Although most of the disparity corresponds to changes in the magnitude of biallelic imbalance, ASE sites switching from either strictly monoallelic to biallelic imbalance or the reverse occur in few genes that are known or predicted to be imprinted, subject to X-chromosome inactivation or A-to-I(G) RNA edited. We also uncovered comparable ASE differences between homokaryotypic MZ twins. The extent of ASE discordance in MZ twins (2.7%) was about 10-fold lower than the expected between pairs of unrelated, non-twin males or females. The results indicate that the observed within-pair dissimilarities in breadth and magnitude of ASE sites in the heterokaryotypic MZ co-twins could not solely be attributable to the aneuploidy and the missing allelic heritability at 21q.

## Introduction

Monozygotic (MZ) twinning entails the partitioning of progenitor cells derived from one zygote collapsing into two sets that form two separate fetuses (co-twins) of nearly identical genotypes. MZ co-twins develop through monochorionic or dichorionic placentation as a result of when the sets of progenitor cells are split. The exact mechanisms that trigger MZ twinning are vague but genetic (Liu et al., 2018), epigenetic, and environmental factors have been implicated (Knopman et al., 2014).

A considerable body of experimental evidence demonstrates that most MZ co-twins are not identical but discordant for (epi)genetic traits (Bennett et al., 2008; Baranzini et al., 2010; Furukawa et al., 2013; Souren et al., 2016) and congenital diseases (Chaiyasap et al., 2014; Huang et al., 2019). In stark contrast to homokaryotypic MZ co-twins, the heterokaryotypic MZ co-twins differ for constitutive chromosomal anomalies (Scott and Ferguson-Smith, 1973; Nieuwint et al., 1999). Typically, a pair of heterokaryotypic MZ co-twins exhibits discordant karyotypes for autosomal or gonosomal aneuploidies (i.e., trisomy 21, trisomy 13, XO or XXY) arising most likely post-zygotically and leading to mosaicism at various degrees (Gilbert et al., 2002; Tachon et al., 2014). Heterokaryotypic MZ co-twins may be discordant for structural chromosomal rearrangements (Leung et al., 2009; Essaoui et al., 2013), including genome-wide copy number variation (CNV) that is also commonplace in homokaryotypic MZ twins (Abdellaoui et al., 2015; Huang et al., 2019). Other likely causes for genotypic discordance in MZ monochorionic co-twins include alterations in gene expression (Buil et al., 2015), parent-of-origin effects associated to abnormal non-random (skewed) X-chromosome inactivation (XCI) (Orstavik et al., 1995), and genomic imprinting (Weksberg et al., 2002; Begemann et al., 2018). There are 43 well-documented cases of heterokaryotypic MZ co-twins in humans (**Table S1**). Most of the reported cases are spontaneous pregnancies, rather than associated with assisted reproductive technology.

Epigenomic studies in heterokaryotypic MZ co-twins are of the most significant value for assessing the effects on gene- and allele-specific expression of an extranumerary chromosomal copy or structural chromosomal disparities in otherwise nearly identical genomes.

Oligo microarray (Yan et al., 2002; Lo et al., 2003; Morley et al., 2004) and genome-wide transcriptome shotgun sequencing (RNA-Seq) studies in multiple biological samples have unveiled that many genes are subjected to the differential transcriptional expression of one allele of a pair of alleles (Dixon et al., 2015; Pirinen et al., 2015; Weissbein et al., 2016). Allele-specific expression (ASE) refers to the departure from the Mendelian 1:1 allelic expression ratio assumption. Typically, the patterns of allele expression include symmetrically (strictly) biallelic, asymmetrically biallelic (biallelic imbalance or allelic bias), and strictly monoallelic (Dixon et al., 2015; Pirinen et al., 2015; Weissbein et al., 2016).

RNA-Seq analysis allows determining the breadth and magnitude of ASE sites simultaneously. At a given experimental condition, each cell type should exhibit an array of ASE sites, an ASE signature, or transcriptome fingerprint, which is expected to be remarkably particular to the individual biological sample. The ASE signatures may be altered by environmental, health, and disease conditions (Moyerbrailean et al., 2016; Weissbein et al., 2016). In essence, the same source of cells from MZ co-twins should exhibit identical ASE signatures. However, studies based on transcriptome sequence analysis disclosed widespread discordance in ASE sites in biological samples from apparently healthy homokaryotypic MZ twins (Cheung et al., 2008; Buil et al., 2015). Therefore, at the RNA level, the occurrence of ASE discordance constitutes a form of a cryptic, unexplained/missing heritability in individuals who share, in principle, “identical” genomes. On the other hand, genome-wide ASE discordance implies that the mechanisms for reliable transfer or flow of genetic information from DNA to RNA within humans are loose, with profound implication(s) for human health and disease (Chakravarti, 2011).

**Figure 1:**
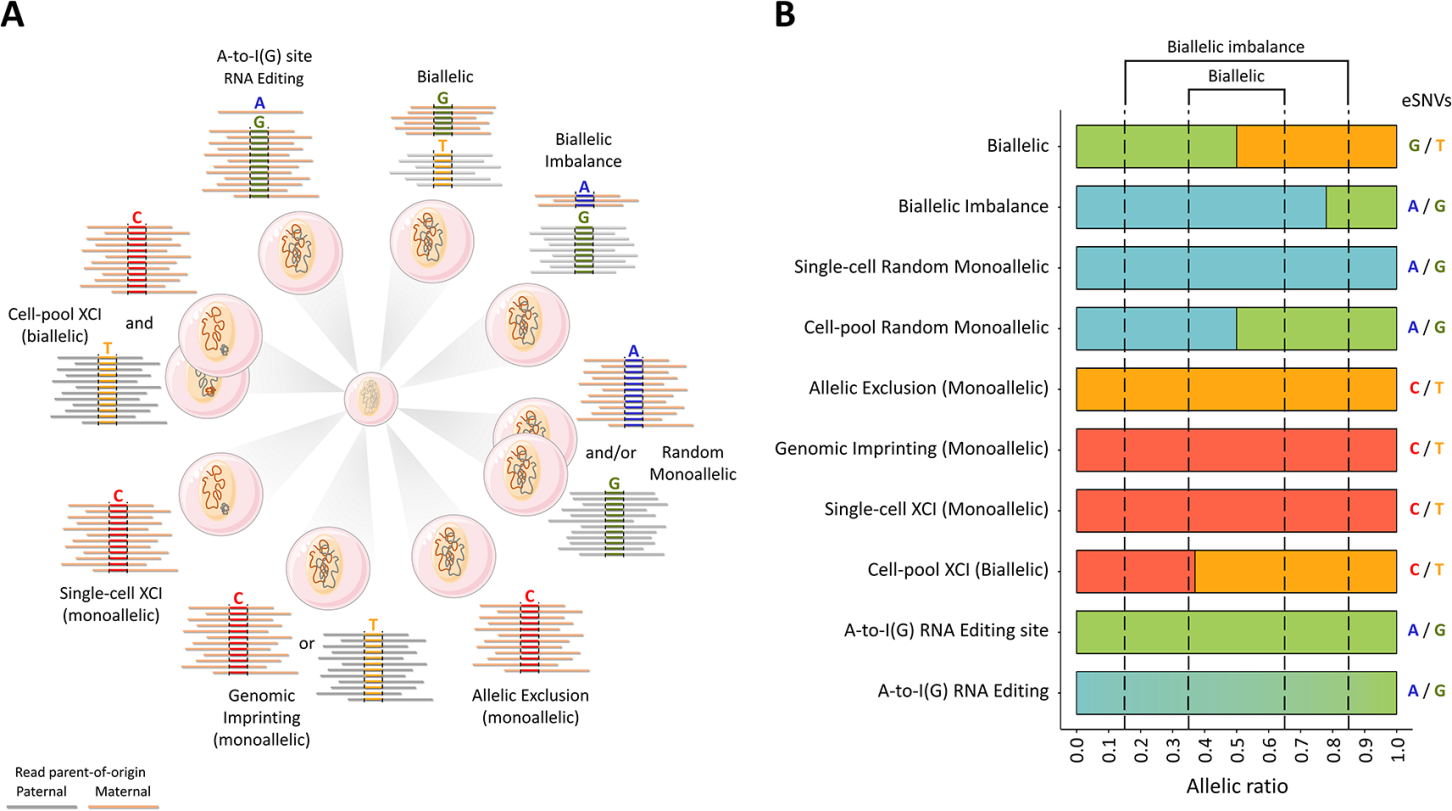
Epigenetic processes involved in allele-specific RNA expression. **(A)** The differential allele expression of genes best reflects dynamic regulation processes consistent with either an allele being preferentially silenced or an inactive allele being restored. The scenarios are for total steady-state RNA, for which a minimum of 12 reads are depicted across the reference and alternative alleles at hypothetic heterozygote or A-to-I(G) RNA editing sites. The breadth and magnitude of the deviation from the expected strictly biallelic 6:6 read ratio may be ascribed to one of several epigenetic regulatory processes involving compensatory and non-compensatory cis-acting variation epistatic to trans-acting variation. The scenarios are organized clockwise: strictly biallelic, biallelic imbalance, random monoallelic, allelic exclusion, genomic imprinting, single-cell X-chromosome inactivation (XCI), cell-pool XCI, and RNA editing. Up to 30% of all tested protein-coding autosomal genes are subjected to clonal (mitotically) stable, random monoallelic expression, which can be either coordinated or uncoordinated (Gimelbrant et al., 2007; Savova et al., 2016a; Savova et al., 2016b; Vigneau et al., 2018). Up to 23% of genes linked to the X-chromosome are expressed from the inactive X (i.e., XCI escapee genes) and, therefore, are biallelically expressed in each female somatic cell (Tukiainen et al., 2017). About 2.6 million ribonucleotide sites genome-wide are known to be subjected to A-to-I(G) RNA editing (Ramaswami and Li, 2014). Thus, the human tissues are, in essence, expression mosaics due to epigenetic-, cis-, and trans-acting covariates. **(B)** The extent of the allele-specific expression for the scenarios illustrated in panel **(A)** using RNA-Seq reads across SNVs in genes known to be subjected to the indicated regulatory processes. *WRB* (biallelic) (Alves Da Silva et al., 2016; De Sa Machado Araujo et al., 2018), *SH3BP5L* (biallelic imbalance) (Baran et al., 2015), *EVC* (random monoallelic) (Gimelbrant et al., 2007), *SNURF* (maternally imprinted) (Gray et al., 1999; De Sa Machado Araujo et al., 2018), *OR2L13* (allelic exclusion) (De Sa Machado Araujo et al., 2018), *DGKZP1* and *AL391244.3* (RNA editing; the present study), *FMR1* (subject to XCI) (Tukiainen et al., 2017).The data supporting the allele ratios depicted in the histograms are presented in **Dataset S1**.

The causes of ASE discordance are associated with (epi)genetic factors, gene-gene, and gene-environment interactions (**Figure 1** **,** **Dataset S1**). For genes that are not subjected to either epigenetic regulatory mechanisms such as genomic imprinting (Baran et al., 2015), and XCI (Tukiainen et al., 2017), ASE mostly relates to the expression effects associated to quantitative trait loci (eQTLs), which can be ascribed to sequence variants of both alleles (cis effect), whereas the extent of the ASE effect relies on trans genetic variants and environmental factors interacting with the cis genetic variants (Buil et al., 2015).

Furthermore, over 2.6 million ribonucleotide sites are known to be post-transcriptionally subjected to allele-specific editing at varying extents in several human tissues, thus contributing, at a much higher degree, to the phenotypic expression of likely mutational sites in the form of differential epitranscriptomes (Li et al., 2009; Ramaswami and Li, 2014; Zhou et al., 2018). Among the genetic factors, there are also differences in meiotic recombination and chromosomal aberrations (Weissbein et al., 2016).

Here, we carried out a comparative computation analysis of RNA-Seq data from heterokaryotypic MZ co-twins discordant for trisomy 21 and homokaryotypic MZ co-twins. We cross-referenced the ASE sites with public data repositories to exemplify the sources and consequences of within-pair disparities to annotate ASE effects in genes that are subjected to the (epi)genetic processes of genomic imprinting, XCI, and RNA editing. We identified considerable ASE disparity between either heterokaryotypic or homokaryotypic co-twins.

## Materials and Methods

### Bioprojects

We used primary (unprocessed) RNA sequence filed data from the Sequence Read Archive (SRA) public experiments in 10 twin pairs, being one pair of heterokaryotypic co-twins and nine pairs of homokaryotypic co-twins. The biological samples included: primary fetal fibroblasts (GEO BioProject PRJNA239814) from the study by Letourneau and collaborators (2014), induced pluripotent stem cells (iPSC) from the study by (Hibaoui et al., 2014) (GEO BioProject PRJNA227902), and cultured B-cells (Epstein-Barr virus transformed lymphoblastoid cell lines from peripheral adult blood B-lymphocyte; GEO BioProject PRJNA170210) (**Dataset S2**). We selected the transcriptome study by Letourneau and collaborators (2014) on a pair of MZ co-twins who were karyotypically discordant for trisomy 21 (T21) of maternal origin (Dahoun et al., 2008), and therefore are heterokaryotypic twins (i.e., co-twins that differ concerning constitutive chromosomal anomalies). Comparative transcriptomics in these heterokaryotypic twins lead to the proposal of the so-called domains of genome-wide gene expression dysregulation in Down syndrome (Letourneau et al., 2014). The case is emblematic because, in addition to the discordant maternal T21 aneuploidy, primary fetal fibroblasts from the MZ twins exhibited missing allelic heritability at 21qter as a result of recombination event(s) (Dahoun et al., 2008). A diagram of the discordant maternal 21q inheritance in the pair of co-twins heterokaryotypic for trisomy 21 is represented in **Figure S1**. To estimate the extent and magnitude of ASE discordance in unrelated, non-twin individuals, we included the RNA-Seq run experiments from BioProject PRJNA316578 (**Dataset S2**), which comprises whole blood samples from two males and two females, mean age 34-year-old, healthy controls.

### Identification, Quantification, and Sorting Out Allele-Specific Expression Sites in Transcriptome Data

We implemented PipASE, an in-house computational pipeline to identify, quantify, and sort out ASE sites in the transcriptome data (**Figure S2**). PipASE scans genome-wide for expressed single nucleotide variants (eSNVs) in high quality aligned reads. We recognize that RNA-Seq read counts and, therefore, expressed allele rates, maybe artifactually made discordant between co-twins as a result from sequencing chemistry and forward/reverse strand biases in the error rate of the high-throughput sequencing technology (Heap et al., 2010; Pickrell et al., 2012; Liu et al., 2014; Soderlund et al., 2014; Hu et al., 2015; Wood et al., 2015; Raghupathy et al., 2018; Richard Albert et al., 2018). Therefore, primary sources of technical artifacts such as systematic errors in sequencing and mapping sequence reads to a haploid reference genome were curbed by including in the PipASE the following specific algorithms that reduce or control the mapping bias: i) relaxing the number of mismatches admitted per string, yet excluding reads with spurious mismatches at the last bases of reads aligning just to one DNA strand; ii) excluding reads aligning around insertions, deletions, and simple tandem repeats; iii) excluding reads mapping to paralogous genomic regions (i.e., segmental duplications); iv) requiring ≥ 12 high-quality read depth to call a candidate informative site, and v) prioritizing the ranking of ASE sites by multiple consistent expression patterns.

Raw reads were trimmed with Trimmomatic (Bolger et al., 2014), and aligned to the hg38 reference genome using the Spliced Transcripts Alignment to a Reference (STAR, v3.5a) software (Dobin et al., 2013). We required uniquely and high-quality mapped reads (MAPQ ≥ 30) by filtering them using the sequence alignment/map tools (SAMtools) (Li et al., 2009). We processed the RNA-Seq data according to the best practice guidance using the ASEReadCounter tool from the open-source Genome Analysis Toolkit (GATK, v3.8), instrumented for variant discovery in high-throughput sequencing data (McKenna et al., 2010; Depristo et al., 2011; Van Der Auwera et al., 2013). Annotated single nucleotide polymorphisms (SNP) and private SNVs were identified using HaplotypeCaller from GATK at each hypothetical heterozygous position according to HapMap (International HapMap, 2003) and database of SNP (Sherry et al., 2001). The annotation of ASE variant site positions to the hg38 reference genome was performed using the R/Bioconductor biomaRt package (Durinck et al., 2005; Durinck et al., 2009). SNP population data (MAF, ancestral allele) were integrated using rsnps package version 0.3.0 (Chamberlain et al., 2018). For the assessment of ASE, the read counts from the replicas were amalgamated, and Q1 values across each informative eSNV site were calculated for all biosamples on a per twin basis. For ASE sites that occurred only once in each set of biosamples, the ASE value was given by the informative run. Thus, ASE sites are supported by at least one informative run. For example, BioProject PRJNA239814, which refers to fetal fibroblasts biosamples collected from the MZ twin pair discordant for trisomy 21, comprises 12 RNA-Seq run experiments, being six per twin. The project includes four biosamples for each twin, and two of which are replicas. For that project, the distribution of informative ASE sites is 51.7, 18.2, 18.5, and 11.6% sites supported by at least 1, 2, 3, and 4 biosamples, respectively. ASE across imputed heterozygous SNP sites was calculated as the difference of RNA-Seq read counts between the two alleles, using the equation ASE =|0.5 — Ref _allele_read count / (Ref _allele _read count + Alt _allele _read count)| The allelic expression imbalance value per site (ranging between 0 and 0.5) is, therefore, a measure of departure from the expected Mendelian 1:1 allelic expression ratio (Babak et al., 2015; Baran et al., 2015). We annotated the ASE data by calculating the expected null reference/alternative ratios and binomial test P-values (Wang and Clark, 2014) using the *binom.tes*t R code function (R Core Team, 2019), and according to their gene structure sequence context (exon, intron, 5´ UTR, 3´ UTR, and intergenic) using the GRCh38.92 Ensembl release 96 in gtf format and the *GenomicFeatures* annotation package in R code (Lawrence et al., 2013). I-square statistical test was used to assess the degree of heterogeneity in the ASE profiles of genes supported by multiple eSNVs. The test is based on the chi-square and degree of freedom values, and it was used to measure the inconsistency of ASE profiles in each gene. We ranked genes according to the following criteria: homogeneity (I-square <30%), moderate heterogeneity (between 30 and 50%), substantial heterogeneity (between 50 and 75%), and considerable heterogeneity (> 75%). The negative I-square values were considered as 0% (Wang and Clark, 2014; Von Hippel, 2015). A flowchart for the PipASE used for scanning and sorting out genome-wide, allele-specific differences between MZ co-twins is shown in **Figure S2**.

### Cross-Referencing With Public Data Repositories

For every ASE site observed in each RNA-Seq sample, we extracted functional information by computational cross-referencing with public databases regarding pathogenic expression-altering or loss-of-function risk variant alleles (Adzhubei et al., 2010; Landrum et al., 2016; Vaser et al., 2016), genomic imprinted genes (Jirtle and Murphy, 2012; Wei et al., 2014; Baran et al., 2015; Pirinen et al., 2015), A-to-I(G) RNA editing sites (Ramaswami and Li, 2014), germline ASE discordant sites in MZ twins (Cheung et al., 2008), and XCI escapee and non-escapee genes (Carrel and Willard, 2005; Cotton et al., 2013; Balaton et al., 2015; Cotton et al., 2015; Tukiainen et al., 2017; Garieri et al., 2018; Shvetsova et al., 2019; Wainer Katsir and Linial, 2019). Allelic expression profiles were validated computationally by data integration with the ASE profiles observed in multiple human tissues from the Genotype-Tissue Expression (GTEx) project (The GTEX Project, 2015), using the *Data Integrator* tool available at the UCSC Genome Browser, that contains track hubs for the second source GTEx data (release V6, October 2015), mainly as previously reported (De Sa Machado Araujo et al., 2018).

### Canonical A-to-I(G) Ribonucleic Acid Editing

ASE sites were queried in the RADAR database, which comprises a list of about 2.6 million rigorously annotated database of A-to-I(G) RNA editing sites. For cross-referencing of the ASE sites, we merged RADAR data version 1 (available online from the RADAR browser) and version 2, which is based on the GTEx RNA-Seq dataset from 30 tissues (hg19; version 6p), and reports RNA editing levels for sites with ≥20 reads (Tan et al., 2017), kindly provided as a flat database by Dr. Jin Billy Li at Stanford University (Ramaswami and Li, 2014). The hg19 coordinates were lifted over to hg38 using “hg19ToHg38.over.chain” file and R scripts based on *AnnotationHub* (Morgan, 2017) and rtracklayer libraries (Lawrence et al., 2009). We limited the analysis to base positions corresponding to canonical A-to-I(G) variants, excluding all SNVs that map within segmental duplications or simple repeats in the hg38 reference genome, using the ShortMatch tool with query strings of 50 bases in length containing the variant at position 26th. The filter-selection step above followed published quality guidelines (Lin et al., 2012b; Ramaswami et al., 2012; Piskol et al., 2013). For every ASE site matching a RADAR reference editing site location, we calculated the A-to-I(G) RNA editing levels as the ratio of G-containing reads divided by the sum of A- and G-containing reads in RNA-Seq experiments of each pair of co-twins. The strength of the co-association between the levels of RNA editing at ASE sites within twin-pairs was measured using linear models in R.

## Results

### Transcriptome-Wide, Allele-Specific Differences Observed in Monozygotic Co-Twins Discordant for Both Trisomy 21 and Recombination

**Figure 2:**
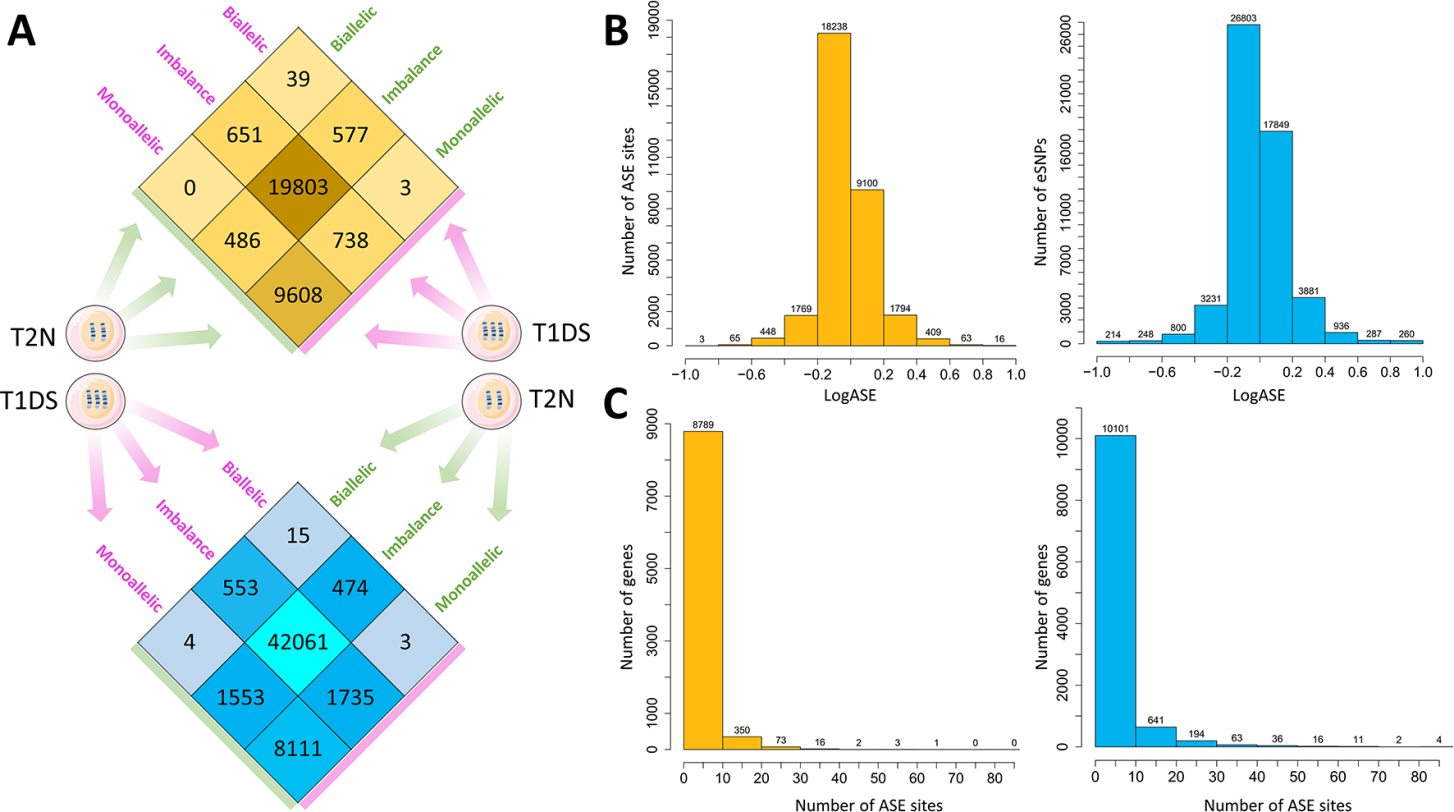
Overview of the breadth and magnitude of allele-specific expression disparity between heterokaryotypic monozygotic (MZ) twins. **(A)** Number of allele-specific expression (ASE) sites distributed by the within-pair status of concordance or discordance in MZ twins heterokatyotypic for trisomy 21 and discordant for maternal 21q inheritance tested in primary fibroblasts (upper panel in orange heat plot) and iPSC (lower panel in blue heat plot). In each cell type, the majority of ASE sites are concordant by biallelic imbalance status in both the trisomy 21 (T1DS) and the normal (T2N) co-twins. On average, the co-twins are discordant in 2,261 ± 1,462.3 ASE sites. **(B)** Comparison of the effect size of the LogASE between fibroblasts and iPSC, respectively. We calculated the log2 of allele-specific expression fold change using the equation *LogASE = log2*(*T*1*DS _ ASE / T*2*N _ ASE*) for each expressed single nucleotide variant in each tissue. LogASE estimates the magnitude of expression change between conditions for the variant. **(C)** Distribution of genes by numbers of ASE sites observed in fibroblasts (orange bars) and iPSC (blue bars).

Recombination and sequence variation are major evolutionary sources of diversity in the human genome. We, therefore, wished first to evaluate how these two forces impacted on ASE in “identical” co-twins. Between the MZ co-twins discordant for T21, we identified 1,227 (3.8%) ASE sites whose allelic patterns were discordant (i.e., monoallelic *versus* biallelic) in fibroblasts and 3,295 (6%) such sites in iPSC (**Figure 2A** **,** **Dataset S3**). We estimated the magnitude of expression change between conditions for the variants called (**Figure 2B**). The bulk of the ASE sites exhibited a LogASE value close to zero, which means that the majority of the ASE sites were not altered in trisomy 21 condition. Importantly, 19 eSNVs were significantly altered in fibroblasts of the trisomy 21 (T1DS) affected twin, being 16 sites with LogASE ≥ 0.8 and three sites with LogASE ≤ −0.8. Noteworthy, 11 implicated genes mapped to the 21q region discordant for maternal inheritance due to a recombination event. Among those genes, *CASP6*, *FAM86GP,* and *PDXDC1*/*PKD1P6* were expressed monoallelically, whereas the *IL17RA* gene was expressed biallelically in fibroblasts from the T1DS twin. In iPSC, we observed 260 eSNVs with LogASE ≥0.8 and 214 ≤ −0.8 annotated in 274 genes (**Figure 2B**). Of the 19 ASE sites with ASE values ≤ −0.8 or ≥ 0.8 in fibroblasts, 14 were also called in iPSC. However, only 10 sites were altered in both cell types with values of ASE ≥ 0.8 (**Dataset S3**), and are located within the 21q region spanning the recombination event. The overall distribution of genes by the numbers of ASE sites observed in fibroblasts and iPSC is shown in **Figure 2C**.

**Figure 3:**
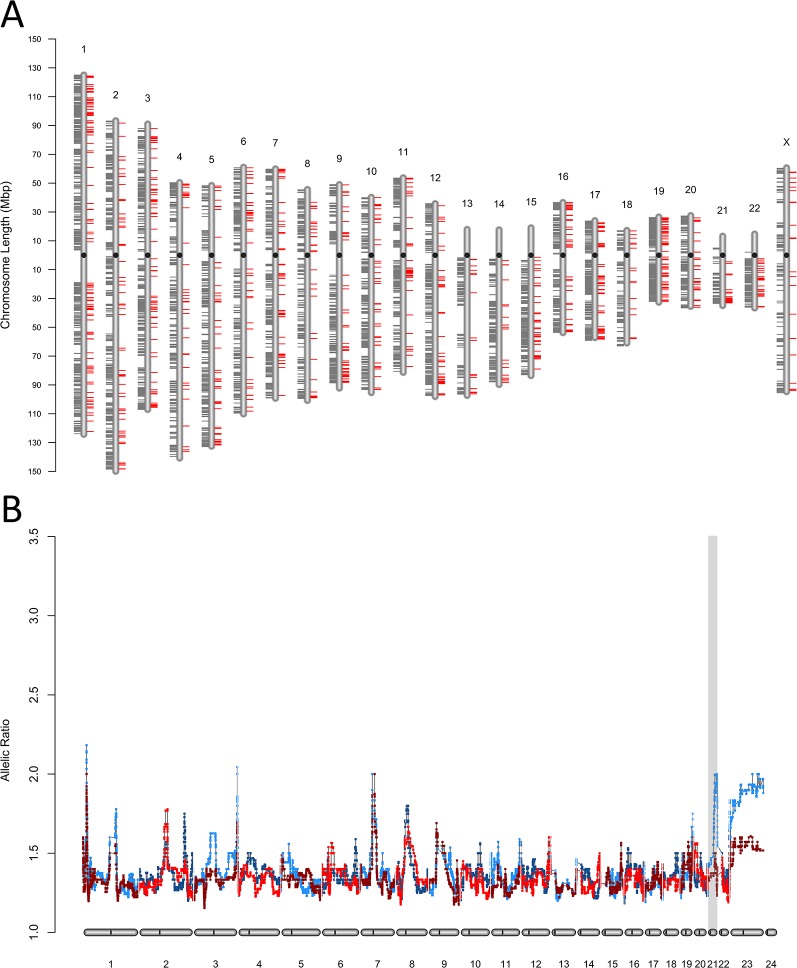
Chromosomal distribution of expressed single nucleotide variants. **(A)** Genome-wide e-karyotyping for the SNPs and variants exhibiting allele-specific expression in primary fetal fibroblasts from the co-twins discordant for T21 and maternal recombination at 21q. Shown is the distribution of all ASE sites that were concordant (gray ticks toward the left of each chromosome ideogram) or discordant (red ticks toward the right side). **(B)** Detection of trisomy 21 by e-karyotyping allelic bias using RNA-Seq data from primary fibroblasts in **(A)**. The gray shading highlights the occurrence of a discordant third copy of chromosome 21 in one twin (T1DS).

The discrepancies in ASE between the MZ co-twins discordant for T21 observed in both fibroblasts (**Figure 3A**), and iPSC (**Figure S3A**) were widespread in the genome (average of 20 ASE sites per Mb). We validated the heterokaryotypic status of the MZ twins discordant for T21 by comparing the within-pair global allele-ratios and plotted them as expression karyotypes (e-karyotypes) (**Figure 3B** and **Figure S3B**).

### Allele-Specific Expression Disparity Observed in Homokaryotypic Twin-Pairs

To begin to sort out the likely causes of the widespread ASE discordance found in co-twins, we examined the breadth and magnitude of discordant ASE sites in nine pairs of co-twins not discordant for aneuploidy and recombination. Surprisingly, the breadth and magnitude of ASE concordance and discordance in the control twin pairs were comparable to those observed in the heterokaryotypic twin pair, with an average 1,074 discordant sites (2.7%) per twin pair (**Figure S4**). The discordant ASE sites were also distributed genome-wide (**Figure S5**). Despite their diverse parental origins, there were, on average, 19,488 ASE sites common within the nine pairs of homokaryotypic MZ twin pairs; 90 (0.46%) sites were discordant in the entire set of twin pairs. Nevertheless, there were, on average, 571 ASE sites discordant in a given twin pair, but concordant in another. The recurrent sites in all nine pairs best reflect identity by state. We note, however, that monochorionic twin developed with a shared circulation, and therefore, the ASE profiles assessed in cultured transformed B-cells isolated at an early age will tend to be similar. Unfortunately, we could not trace the chorion type of the nine homokaryotypic twin-pairs, which were sampled at the age ranging 19 to 65 (**Dataset S2**).

For the entire set of MZ twin pairs, the average distribution of eSNVs per gene was the following: 34.3% (n = 3,162) of genes were called by one eSNV; 57.8% (n = 5,333) were supported by 2 to 10 eSNVs; 7.9% (n = 729) were called by 11 to 200 eSNVs; 0.02% (n = 2.4) were called by 201 to 500 eSNVs, and 0,01% (n = 1.1) exhibited >500 eSNVs (**Dataset S4A and S4B**). We carried a statistical test for heterogeneity to query for intervention effects (variation in effect estimates beyond chance) across a given genomic region. For the entire set of biosamples, we found, on average, that 43.6% (n = 2,619) of genes supported by multiple eSNVs exhibited considerable homogeneity across the eSNV profiles; 3.8% (n = 225) had moderate heterogeneity; 6.1% (n = 366) had substantial heterogeneity; and 46.5% (n = 2,795) had considerable heterogeneity (**Dataset S4C and S4D**). We note that genes exhibiting considerable heterogeneity are large (on average 130 Kbp, i.e., *CD226*) and are supported on average by 7.2 (range 2 to 318) eSNVs. Conversely, the most homogeneous profiles are in genes with an average size of 12 Kbp (i.e., *JRK*), which are supported on average by 3.7 eSNVs (range 2 to 38 sites). Moreover, comparing genes supported by the same number of eSNV (i.e., 30 sites), we note that the eSNVs are distributed differently, toward the 3´UTR in genes ranked as homogeneous (i.e., *LGALS8* and *PLEC*) and spread along the gene body in those ranked as heterogeneous (i.e., *CD226* and *GLEC17A*).

We also validated the homokaryotypic status of the nine MZ control twin pair by comparing the within-pair global allele-ratios and plotted them as e-karyotypes (**Figure S6**). Jointly, the e-karyotyping analyses demonstrate that there is pervasive missing allelic heritability between the transcriptome of MZ co-twins and that the bulk of the ASE site within-pair disparities in the heterokaryotypic co-twins cannot be solely attributed to the differential occurrence of aneuploidy and the missing allelic heritability at 21q.

### Allele-Specific Expression Disparity Observed in Unrelated, Non-Twin Males and Females

Unrelated, non-twin males and females exhibited comparable extents of ASE discordance genome-wide: 24.8% (6,546/26,371 eSNV sites) in males (**Dataset S5A**) and 25.57% (5,992/23,431 eSNV sites) in females (**Dataset S5B**). Therefore, the extent of ASE discordance in unrelated, non-twin males and females is about 10-fold higher than the observed between pairs of MZ twin-pairs (2.7%). In the unrelated male and female set, 47.4 and 45.4% of genes supported by ≥ 2 eSNVs, respectively, exhibited considerably heterogeneous ASE profiles, whereas 43.4 and 45.5% of genes were ranked as considerably homogeneous (**Dataset 5C**). Similar to the finding in MZ twins, genes exhibiting considerable heterogeneity are large (on average 83 Kbp, i.e., *GAK* in males and 221 Kbp in females, i.e., *SAMD3*) and are supported on average by 8.2 (range 2 to 104) eSNVs in males and 7.7 (range 2 to 106) eSNVs in females. Conversely, the most homogeneous profiles are in genes with an average size of 18 Kbp (i.e., *UBE2I* in males and *EEF1D* in females), which are supported on average by 3.7 (range 2 to 39) eSNVs in males and 3.5 (2 to 36) eSNVs in females. Again, comparing genes supported by the same number of eSNVs (i.e., 25 sites), we note that the eSNVs are distributed differently, toward the 3´UTR in genes ranked as homogeneous (i.e., *HCP5* and *PRRC2B* in males and *AC004151.1* and *NOTCH1* in females) or spread along the gene body in those ranked as heterogeneous (i.e., *FCGBP* and *GAK* in males and *SAMD3* and *SYNE3* in females).

### Assessment of the Underlying Causes of the Observed Pervasive Missing Allelic Inheritability

The underlying causes of the observed pervasive missing allelic inheritability can include i) genome-wide DNA sequence variations within pairs of MZ co-twins, as supported by recent findings in MZ twins discordant for autism spectrum disorder (ASD) using whole-genome sequencing (Huang et al., 2019) and ii) differential expression of alleles. Given that none of the ten MZ twin pairs referred here has genomic sequences available in public repositories, we first cross-referenced the observed ASE sites with data about the distribution of eSNVs reported between the MZ co-twins discordant for ASD (Huang et al., 2019). On average, the MZ co-twins discordant for ASD exhibited 54 eSNVs disparities annotated in exons, 3,912 in introns, 13 in 5´ UTR, and 74 in 3´ UTR for 2,786 genes (**Table S2**). Remarkably, between either the MZ co-twins heterokaryotypic for T21 or the homokaryotypic MZ co-twins, we identified, on average, 10,111 ASE discordant sites in annotated exons, 8,037 in introns, 2,066 in 5´ UTR, and 18,032 in 3 UTR for 8,495 genes. Thus, a mean 120-fold increase in discordant ASE sites per annotation category. This fold difference cannot be attributed solely to the average distribution rate of discordant eSNVs of 1.1x10^−4^ per exonic site reported across human genes between the genomes of MZ co-twins (Huang et al., 2019). Furthermore, there is a 20-fold deficit in ASE sites annotated in intergenic regions as compared with the number of eSNV sites discordant by whole genome sequencing, which supports the view that the biased distribution of ASE sites discordances within genes may be biologically relevant. We also validated some of the ASE discordant sites by cross-referencing with the sets ASE sites in MZ twins from the study by Cheung et al. (2008) (**Dataset S3**).

Next, we cross-referenced the ASE sites with data about genes known or predicted to be expressed from one allele at a time through genomic imprinting, XCI, and A-to-I(G) RNA editing. Overall, we identified discordant ASE sites in either 205 known or candidate imprinted genes (**Dataset S3**), 12 X-linked genes (**Table S3**), and 3,955 sites likely subjected to A-to-I(G) RNA editing (**Dataset S3**).

### Allele-Specific Expression Switching in Imprinted Genes

We note that, on average, 4,574 ASE sites were monoallelic concordant within co-twins. Annotation of those sites revealed that 8,867 genes exhibited multiple monoallelically eSNVs with no biallelically expressed sites (**Datasets S3** **,** **S6**–**S14**). Among those genes, we annotated five known imprinted genes (*DR1*, *BRD2*, *VARS2*, *MEG3,* and *H19*), each one ranked with ≥8 eSNVs. Cross-reference of those genes with secondary data from the GTEx project validated their monoallelic expression in multiple tissues (**Dataset S15**), and therefore their imprinted status (Jirtle and Murphy, 2012; Wei et al., 2014; Baran et al., 2015; Pirinen et al., 2015). Unfortunately, the GTEx project does not include samples of embryonic fibroblasts, iPSC, or B-cells. In contrast, most other genes ranked with ≥8 monoallelic eSNVs were expressed biallelically in multiple tissues in the GTEx database (**Dataset S15**). We speculate that the monoallelic concordance at multiple sites observed in the co-twins reflects extended homozygosis, rather than parent-of-origin effects. We note five gene exceptions. First, the *AC091729.3* gene, which exhibited an average of 12 monoallelically eSNVs in two of the ten MZ co-twin pairs, is also expressed monoallelically in an isoform-specific fashion with four SNPs in 35 tissues in the GTEx samples (**Dataset S15**). Second, the *SPINK5* gene with 11 monoallelically eSNVs, expressed monoallelically exclusively in the bladder in the GTEx data. While the ASE profile across the *AC091729.3* gene was homogeneous, the *SPINK5* gene ranked moderately heterogeneous. We view these two genes as potential leads and suggest that the *AC091729.3* gene is subjected to isoform-specific genomic imprinting, whereas the *SPINK5* gene is imprinted in a tissue-specific manner. Third, the known imprinted genes *SNURF*, *SNHG14*, and *ZNF264,* which are expressed monoallelically in multiple tissues in the GTEx samples, exhibited ≥ 10 biallelically imbalance eSNVs in iPSC (**Dataset S15**). Interestingly, various biallelically imbalance eSNVs in these three genes are listed in the RADAR database and are likely subjected to A-to-I(G) RNA-editing: 5 out of 16 eSNVs (*SNURF*), 19/35 (*SNHG14*), 4/14 (*ZNF264*) (**Dataset S3**). We, therefore, suggest that the epitranscriptome modification of these gene products by RNA editing alters their expected imprinting phenotype (at least *in vitro*) in iPSCs.

### Estimated Impact of Canonical A-to-I(G) Ribonucleic Acid-Editing on Allele-Specific Expression Disparity

**Figure 4:**
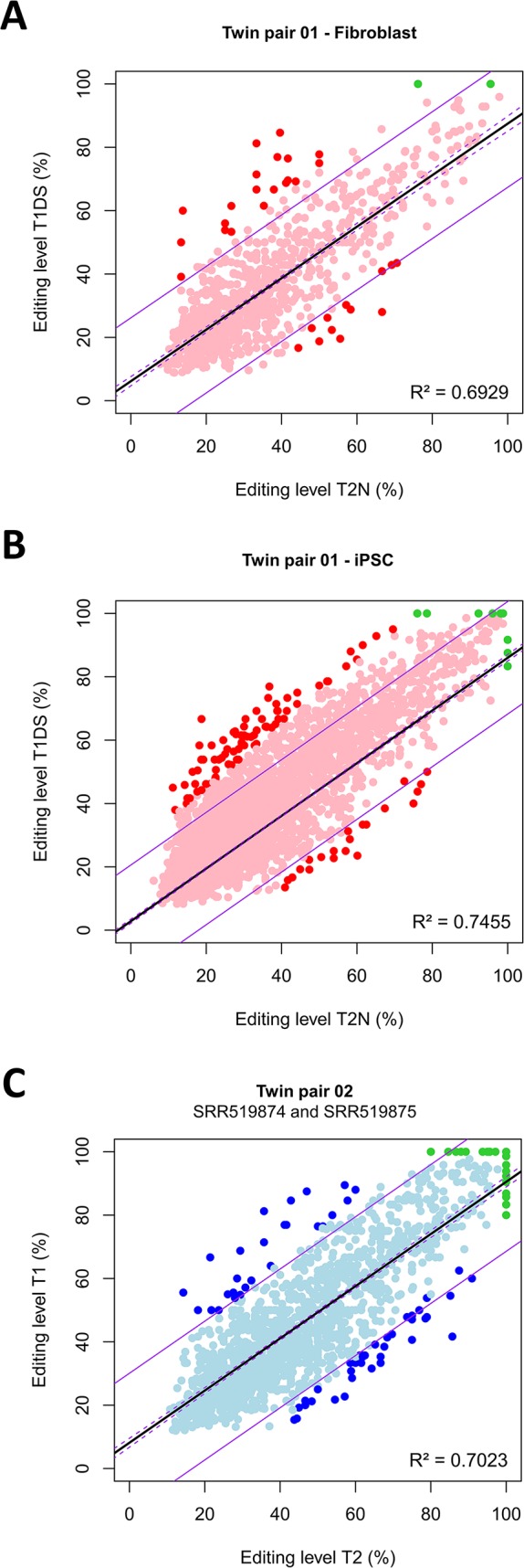
Within twin-pair disparities in allele expression proportions at expressed single nucleotide variants (eSNVs) that are coincident with canonical A-to-I(G) RNA editing sites. Shown is the distribution of eSNVs that positionally match canonical RNA editing sites between heterokaryotypic co-twins, assayed either in fetal fibroblasts **(A)**, fetal fibroblast-derived iPSC **(B)**, or between homokaryotypic co-twins tested in culture-B-cells **(C)**. Each dot corresponds to an eSNV. The vast majority of sites exhibited a concordant biallelic imbalance profile (pink and light blue dots). Red dots represent eSNVs that were discordant between co-twins in that they showed allelic proportions differences higher than 25%, regardless of the discordance or concordance in the karyotype. Green dots represent eSNVs that exhibited discordant allelic profiles, being biallelic in one twin and monoallelic in the other. The linear models (solid black lines), the confidence interval of the models (broken purple lines), and the predictions (solid purple lines) were constructed using R. Model equations: (A) Y = 6.15718 + 0.81302X; (B) Y = 2.832681 + 0.831631X; (C) Y = 8.15439 + 0.82362X. For all pairs, *P* < 2.2e−16.

The number of ASE sites that positionally correspond to canonical A-to-I(G) sites was, on average, 2,012 ± 786 per twin pair (**Datasets S3** **,** **S6**–**S14**), and all the sites cover 419 ± 116 genes. The vast majority of sites exhibited a concordant biallelic imbalance profile (pink and light blue dots in **Figure 4** and **Figure S7**). Thus, within the co-twins, there was an overall concordance in the biallelic imbalance state. The number of sites that exhibited discordant allelic profiles, being biallelic in one twin and monoallelic in the other, was however minimal, albeit more abundant in the homokaryotypic than in the heterokaryotypic co-twins (green dots in **Figure 4** and **Figure S7**). This observation indicated that in the cells analyzed, few eSNVs were 100% edited (i.e., expressed strictly monoallelically) in the complete set of 10 pairs of twins. Between the heterokaryotypic co-twins, there were only seven such discordant sites, being monoallelically expressed in T1DS and biallelically imbalance in the normal co-twin (**Figure 4**). The seven discordant sites occur in seven protein-coding genes, including *CD46* (an immune type I receptor) and *ING5* (a tumor suppressor).

In the heterokaryotypic twins, 117 expressed genes exhibited high proportions of ASE sites (≥ 4 sites per gene) coincident with RNA editing sites. Notably, for the *CYP20A1* and *ZNF621* genes, 74 and 77% of all ASE sites are canonical A-to-I(G) sites validated in the RADAR database. The extent of allele imbalance ranged from 6 to 98%. However, about 5% of all sites exhibited discordant RNA editing levels higher than 25% between co-twins, regardless of whether hetero- or homokaryotypic conditions (red and blue dots in **Figure 4**, **Figure S5** and **Dataset S3**). For example, the gene *CYP20A1* presented the highest percentage of allele imbalance between the heterokaryotypic co-twins in fibroblasts (T1DS = 81.25%/T2N = 33.33%) whereas in iPSC the gene *GCFC2*/*MRPL19* exhibited the highest discrepancy (T1DS = 66.66%/T2N = 18.75%).

Since A-to-I(G) RNA-editing of mRNAs can create stop codons (protein-truncating effect variants) or result in non-synonymous mutations, it was important to annotate the sites with discordant allelic proportions. In the heterokaryotypic co-twins, none of the annotated sites created stop codons, but nine sites are predicted to cause non-synonymous mutations (**Dataset S16**). The cyclin-dependent kinase 13 gene *CDK13*, presented two non-synonymous editing sites that change lysine (Lys; chr7_39950928) and glutamine (Gln; chr7_39950949) to arginine. Within the 5,372 eSNVs annotated as canonical RNA editing sites in the transcriptomes of the homokaryotypic twin pairs, none creates stop codons, and 21 sites correspond to non-synonymous mutations (**Dataset S16**).

### MZ Co-Twins Are Discordant in the Allele-Specific Expression of X-Chromosome Inactivation Non-Escapee Genes

We scanned for discordant ASE sites in X-linked genes within the seven female twin pairs, and integrated data for the intersected sites about the XCI classification status from public repositories (Carrel and Willard, 2005; Cotton et al., 2013; Balaton et al., 2015; Cotton et al., 2015; Tukiainen et al., 2017; Garieri et al., 2018; Shvetsova et al., 2019; Wainer Katsir and Linial, 2019). The analysis was restricted to non-escapee genes because, in pooled cells, those genes must exhibit biallelic expression profiles. For this specific analysis, we only accepted gene products that displayed at least two discordant eSNVs (i.e., monoallelic in one twin *versus* biallelic in the sister twin). For the heterokaryotypic discordant co-twins, there was ASE disparity in the *UBL4A* gene products in fibroblasts, whereas, in iPSC, there was ASE disparity in the *FANCB* and *FTX* gene products (**Table S3**). Three control twin pairs expressed genes with at least two eSNVs: *TAB3*, *WDR44,* and *XIAP* genes in twin pair 05; *IDS*, *MAP7D3*, *RLIM*, *RPL10*, *SLC9A7*, *TBC1D25*, *TLR7*, *XIAP,* and *ZNF275* genes in twin pair 08; and the *ZNF275* gene in twin pair 09 (**Table S3**). None of the ASE disparities above are A-to-I(G) RNA editing sites in the RADAR database.

### The Overall Impact of Allele-Specific Expression of Pathogenic Variants

We annotated 32 eSNVs associated with 131 human pathologies in the transcriptomes of the heterokaryotypic twins (**Dataset S17A**). Most pathogenic eSNVs are linked to autosomal recessive phenotypes and were coexpressed with the wild type allele, likely outbalancing the predicted deleterious effects. Four pathogenic alleles (rs1799990*G > A, rs1800562*G > A, rs200855215*A > G, and rs4784677*A > G) were expressed monoallelically, and are associated with Jakob-Creutzfeldt disease (OMIM #123400), hemochromatosis (OMIM #235200), Leber optic atrophy (OMIM #535000), and Bardet-Biedl syndrome 2 (OMIM #615981), respectively. One pathogenic allele (rs11583680*C > A), associated with autosomal dominant familial hypercholesterolemia (OMIM #603776), was also coexpressed with the wild type allele. In the homokaryotypic twin sets, 23 eSNVs, predicted to be pathogenic in the ClinVar database, predominantly coexpressed with the wild type alleles (**Dataset S17B**). For example, rs1799958*G > A, associated with deficiency of butyryl-coenzyme A dehydrogenase (OMIM #201470), was coexpressed with the wild-type allele in MZT3.

### Evidence for Expressed Mitochondrial Microheteroplasmy

We also identified an ASE form of mitochondrial microheteroplasmy (Souren et al., 2016), albeit at lower limits, in all 10 MZ twin pairs, demonstrated by the presence of 237 eSNVs (median number of 25 eSNVs per dataset, **Table S4**). The observed limited number of mitochondrial eSNVs does not relate exclusively with the early embryonic age at sampling because the age of the control twin pairs ranged from 19- to 65-year-old (median age 26 years) (**Dataset S2**). Thus, for the set of donors investigated, we did not observe the accumulation of mitochondrial eSNVs with age (Smigrodzki and Khan, 2005).

Lastly, we queried ClinVar, PolyPhen, and SIFT public databases for evidence about the pathogenicity prediction for the mitochondrial eSNVs to assess the most functionally crucial mitochondrial point mutations. Fifteen eSNVs are predicted to be likely pathogenic in at least one database (**Table S4**). For example, rs28358569*A > G, monoallelically expressed in MZT9, is related to mitochondrial non-syndromic sensorineural hearing loss (OMIM #500008) and aminoglycoside-induced deafness (OMIM #580000); rs193302980*C > T and rs2853508*A > G are related to familial breast cancer (OMIM #114480).

### Gene Ontology Analysis of Discordant Allele-Specific Expression Sites

In fibroblasts, the *CASP6* and *PDXDC1* genes, represented by ASE sites exhibiting biallelic to monoallelic switch (LogASE ≥ 0.8) within the heterokaryotypic co-twins were related with nitrogen compound and organic substance metabolic processes (**Dataset S18A**). On the other hand, *IL17RA*, the only gene with LogASE ≤ −0.8 and mapping outside the well-characterized 21q recombination region, is enriched in immunological processes such as leukocyte migration, signal transduction, cytokine production, and cell activation (**Dataset S18B**). In iPSCs, most of the genes (72 of 100 genes) with discordant ASE sites (either bi-to-mono or mono-to-biallelic switches) are related to the regulation of biological process (**Datasets S9C and DatasetsS9D**).

## Discussion

We aimed to compile variant sites with expression profiles that are dissimilar between MZ co-twins who are discordant or not for a specific condition. Our scanning strategy permitted the identification, quantification, and classification of differential allelic expression by way of ASE discordant sites (i.e., eSNV) occurring genome-wide between co-twins who are either discordant or not for T21. Remarkably, the breadth and magnitude of ASE discordant sites were high and comparable between either heterokaryotypic or homokaryotypic co-twins. On average, we identified about 1,342 ASE discordant sites in the 10 pairs of MZ co-twins.

The extent of the ASE sites in T21 discordant co-twins was comparable between the non-discordant co-twins, assayed in three cell types (fibroblasts, iPSC, and B-cells). Overall, the analyses indicate that ASE discordance between MZ co-twins stems from aneuploidy, recombination, genomic imprinting, and RNA editing. We interpret the widespread occurrence of ASE discordance between MZ co-twins as being the result of sister chromatid-specific alterations in transcription. The discordant ASE sites observed between co-twins best reflect a combined effect of genetic and epigenetic processes on differential allele expression.

We note that e-karyotyping unveils dynamic arrays of ASE sites that can be considered as signatures that exhibit remarkable singularity to the individual biological sample. For example, for the heterokaryotypic co-twins, the sets of eSNVs observed in fibroblasts or iPSC do not overlap entirely. Overall, 38.67% (n = 24,103) of ASE sites were called in both fibroblasts and iPSC samples, 804 (3.3%) of which exhibited discordant allele-expression profiles in fibroblasts, but concordant in iPSC. Similarly, 1,318 sites (5.4%) were concordant in fibroblasts but discordant in iPSC. Moreover, 187 sites (0.7%) were discordant in both sample types. The relative lack of overlap among the experiments is likely explained by the differential expression of genes in these cell types. Therefore, e-karyotyping signatures might have forensic value and resolution power to discriminate clinically non-discordant co-twins. The e-karyotyping signatures may be specific to the level of each experimental condition for the same source of a biological sample. In principle, no sharing of e-karyotyping signatures is expected to occur within co-twins.

The observation of allelic bias is becoming commonplace in high-throughput transcriptome analyses (Deveale et al., 2012; Marinov et al., 2014; Metsalu et al., 2014; Wood et al., 2015; Weissbein et al., 2016). It is acceptable that the expression of most genes can be altered among biological sample replicas and that the total cellular RNA is not constant. Allelic bias in RNA-Seq can be, in part, attributed to the differential impact of the *in vitro* culture conditions (Gimelbrant et al., 2007; Weissbein et al., 2014). Thus, part of the ASE discordance observed between fibroblasts and iPSC in co-twins in our analysis may be due to acquired chromosomal abnormalities during the iPSC derivation and their propagation in culture.

Because the onset of MZ twinning, XCI, and genomic imprinting may occur at about the same time of embryological development (Machin, 1996), twining may affect the distribution of cells bearing the inactivated X-chromosome or abnormal epigenetic marks of imprinting, and therefore, the varying manifestation of allelic differences from these processes. Surprisingly, the effect primarily occurs in female co-twins rather than male co-twins, and, thus, it is likely due to the presence of more than one X-chromosome in females (Lubinsky and Hall, 1991; Matias et al., 2014). Furthermore, there are cases of MZ female co-twins discordant for skewed XCI and imprinted disorders (Orstavik et al., 1995) and non-imprinted diseases (Bennett et al., 2008). Interestingly, 1,050 ASE sites map to 205 known and candidate imprinted genes. ASE discordance, yet at a considerably lower extent than the described here, has been reported between at a pair of MZ “identical” co-twins clinically discordant for multiple sclerosis (Souren et al., 2016). Importantly, altered allelic expression of two imprinted genes (*ZNF331* and *GNAS*) and five non-imprinted genes (*ABLIM1*, *UBE2I*, *KIAA1267*, *CD6*, and *ATHL1*) were detected between the multiple sclerosis discordant co-twins.

Fourteen X-linked genes subjected to XCI (the non-escapee genes *UBL4A*, *FANCB*, *FTX, TAB3*, *WDR44*, *XIAP*, *IDS*, *MAP7D3*, *RLIM*, *RPL10*, *SLC9A7*, *TBC1D25*, *TLR7*, and *ZNF275*) in four female twin pairs exhibited ASE disparities in which one co-twin presented a biallelic profile and the sister female showed a monoallelic pattern. The RNA-Seq experiments were from pooled cells rather than single-cells and, thus, a biallelic model is the anticipated expression profile for non-escapee genes. The observed ASE disparities cannot be attributed to differences in cell culture conditions that result in decreased percentage of XCI mosaicism, which are expected to affect the expression profiles of all the 457 genes that are subjected to XCI (Carrel and Willard, 2005; Cotton et al., 2013; Balaton et al., 2015; Cotton et al., 2015; Tukiainen et al., 2017; Garieri et al., 2018; Shvetsova et al., 2019; Wainer Katsir and Linial, 2019).

What mechanisms might explain the observed ASE discordance in MZ co-twins? Data from prior RNA-Seq studies in co-twins (Baranzini et al., 2010; Lin et al., 2012a; Brown et al., 2014; Hibaoui et al., 2014; Buil et al., 2015; Dixon et al., 2015; Ding et al., 2017; Santoni et al., 2017) indicate that the differential allele expression of autosomal genes best reflects dynamic regulation processes consistent with either an allele being preferentially silenced or an inactive allele being restored. The biallelic expression of genes is a regulatory mechanism that outbalances the harmful effects of pathogenic expression-altering or loss-of-function risk variant alleles (Adzhubei et al., 2010; Landrum et al., 2016; Vaser et al., 2016). In each human euploid somatic cell, autosomal genes are anticipated to be symmetrically expressed from both the parental alleles in a cell type-specific manner throughout development. However, the biallelic RNA expression pattern is not a phenotypic hallmark of all genes since 10–30% of human autosomal genes assayed for polymorphic variant sites [i.e., expressed SNPs (eSNPs) or eSNVs] are dynamically subjected to the epigenetic phenomena of clonal (mitotically) stable, random monoallelic expression (Gimelbrant et al., 2007; Savova et al., 2016a; Savova et al., 2016b; Savova et al., 2017), or allelic bias (Dixon et al., 2015). Most enigmatic, genes that are biallelically expressed in a cell can be regulated in a neighboring cell to randomly switch their RNA expression from biallelic to monoallelic at a time (Chess, 2013; Eckersley-Maslin and Spector, 2014; Eckersley-Maslin et al., 2014). Also, distinct subsets of autosomal and X-linked genes are subjected to epigenetic silencing of one allele, in a parent-of-origin dependent manner by autosomal genomic imprinting (Baran et al., 2015) or in a random fashion by XCI (i.e., in females) (Tukiainen et al., 2017).

ASE discordance in X-linked genes that are subjected to XCI has been reported between MZ female co-twins in humans (Cheung et al., 2008; Antonarakis et al., 2018) and in mice (Wang et al., 2010). Subtle departure from equal allelic expression ratios is often genetically determined in cis (i.e., eQTLs) and trans, but part of the disparity can also be ascribed to the random sampling effect of X inactivation (Carrel and Willard, 2005; Cheung et al., 2008; Cotton et al., 2013; Balaton et al., 2015; Cotton et al., 2015; Tukiainen et al., 2017; Garieri et al., 2018; Shvetsova et al., 2019; Wainer Katsir and Linial, 2019). Moreover, allelic imbalance on the X-chromosome could also affect autosomal allelic expression. Notwithstanding, we note that the extent of ASE discordance in homokaryotypic male twin-pairs (on average, 3.2%, n = 1,478 discordant sites) is comparable genome-wide to that in female twin pairs (2.5%, n = 958 discordant sites).

There are genetic and functional consequences of the autosomal variant sites in genes that are expressed from a single allele in one cell at a time. Mainly, i) they bestow more extensive genetic diversity in humans (Savova et al., 2016a); ii) they often are gain-of-function rather than pathogenic expression-altering or loss-of-function risk variants (i.e., for neurodevelopmental disorders), and influence expression variance in cis; iii) the range of expression level of monoallelically expressed genes is higher than biallelically expressed genes (Savova et al., 2017); iv) ultimately, they increase cell-to-cell expression variability with a beneficial impact of avoiding genetic disease phenotypes (Savova et al., 2016a).

The extranumerary chromosome 21 in trisomic cells of Down syndrome patients is well known to result in genome-wide dysregulation of gene expression represented by chromosomal domains with genes whose expression levels are copy-dosage compensated, upregulated, or downregulated as compared with euploid cells (Letourneau et al., 2014). In the co-twins discordant for T21 and 21q recombination, the ASE discrepant profiles can be viewed ultimately as unexplained heritability or missing heritability due to the discordance in trisomy 21, recombination at 21q, altered genomic imprinting, random monoallelic expression, and RNA editing. Allelic imbalance (allelic-specific heterogeneity) in dosage-sensitive genes can arise by an stochastic adaptive regulation in both euploid and aneuploid cells as a consequence of low-level mRNA abundance and increased transcriptional burst frequency, rather than burst size (Deng and Disteche, 2019; Larsson et al., 2019; Symmons et al., 2019). The extent of the biallelic imbalance across eSNVs most likely reflects a gene network expression effect operating in the form of eQTLs (Mott et al., 2014; Pettigrew et al., 2016). Thus, part of the unexplained heritability or missing heritability could be explained by differences in cell-specific gene interactions. Moreover, the ASE profile discrepancies between fibroblasts and iPSC could be due to non-imprinted parental origin effects in each cell type associated with the aneuploidy. For example, the rS93366794 eSNP exhibited a concordant biallelic profile in iPSC but discordant in fibroblasts, being monoallelic in the T21 twin and biallelic in the normal co-twin. Interestingly, in an RNA-Seq study of a healthy brain, the *WRD4* gene bearing the rS93366794 site was reported to be expressed monoallelically from the paternal allele, but a mono-to-biallelic switch occurred in the offspring with *versus* without ASD (Lin et al., 2018).

Although the analysis presented here allowed the identification of a pervasive disparity in ASE profiles between co-twins, the biological significance of the extent and breadth of the observed differences in allele expression must only be assessed by independent experiments. However, the following results are of worth noting: i) among the eSNVs, there were several alleles known to be associated with disease conditions or predicted to be pathogenic; ii) the canonical imprinted gene *SNURF*, which is expressed monoallelically in over 50 tissues in the GTEx dataset, was expressed biallelically in iPCS; iii) in all the 10 twin sets, there was expressed mitochondrial microheteroplasmy; iv) among all the genes expressed in the 10 twin pairs, there were 55 ± 17 genes that exhibited elevated proportions (ranging from 50 to 100%) of ASE sites coincident with RNA editing sites.

The breadth and magnitude of ASE discordance disclose unprecedented epigenomic-wide inter-individual variation occurring in MZ co-twins. Prior ASE studies in MZ twins are restricted to a specific gene or gene sets and, therefore, do not uncover the apparent state of pervasive missing allelic heritability in MZ co-twins shown in the present study. Although independent validation through wet experimentation (i.e., allele-specific quantitative reverse transcription-PCR, RNA-fluorescence *in situ* hybridization, or allele-specific pyrosequencing) is required for the biologically relevant candidate ASE discordant sites (here regarded as potential leads), two critical implications emerge from the epigenomic-wide inter-individual variation observed in MZ co-twins: i) as in the case of inter-individual variation in DNA methylation (Maunakea et al., 2010; Bell et al., 2012; Young et al., 2017; Garg et al., 2018), ASE discordance may have to be looked at when assessing and calculating the impact of phenotypic variation in the differential susceptibility to specific human conditions and diseases (Skipper, 2008; Sun et al., 2013); ii) ASE discordance also might be considered instrumental for developing RNA biomarker signatures for forensic body fluid identification and kinship analysis (Blay et al., 2019).

The present systematic and integrative meta-analysis has three important limitations: sample size, the certainty of correct calling a positive eSNV site for a theoretical heterozygote position, and comparisons made in three different cell types. First, the experimental setting must be viewed as a case-study regarding a heterokaryotypic MZ twin pair discordant for trisomic 21 and chromosome 21 distal recombination. Reported cases of heterokaryotypic MZ twin pairs are rare. In **Table S1**, we listed all relevant cases studied in the literature. Notwithstanding, there is only one RNA-Seq public (i.e., not controlled) study in a pair of heterokaryotypic MZ twins, namely, the selected discordant index case. We used the index case as a reference case to investigate whether the underlying discordance in karyotype and recombination affect the ASE profiles. We initially hypothesized that any likely discordance in ASE differences will be restricted to chromosome 21 and that the differences will be more significant across and beyond the recombination event. However, the initial analysis indicated the occurrence of genome-wide rather that chr21-restricted ASE discordance. To investigate whether the observed genome-wide ASE discordance was limited to the unique index case, we investigated nine pairs of homokaryotypic MZ twins. Surprisingly, we observed genome-wide discordance in ASE, similar in breadth and magnitude to that observed in the index case, albeit in different cell lines. Second, to decrease the chances of false-positive ASE calls, we called eSNV sites using base quality control Q30 and ≥12 read depth, which are selecting criteria that excel in stringency published reports of the kind (Q20 and ≥8 reads) (Cheung et al., 2008; Baran et al., 2015; Tan et al., 2017; Tukiainen et al., 2017). Because the probability of correct SNV calling increases at higher coverage levels for a theoretical heterozygote position, we provided results for three read coverages (12, 20, and 40 reads). Coverage of 40 reads provides a 99,9% probability of correct SNV call (**Datasets S3**-S14). Third, the observation of genome-wide ASE discordance in the same type of cell lines (nine MZ twin pairs) and different cell lines (index case) is a very reassuring remark. Cheung et al. (2008) used 100K Affymetrix SNP array on the same set of homokaryotypic MZ twin biosamples and identified 201 SNPs with significant evidence of differential allelic expression. Of those, we confirmed 137 eSNVs as discordant, 38 sites of which were common to the nine twin-pairs (**Datasets S4**–S14). Unfortunately, no public next-generation sequencing data are available for DNA-Seq and RNA-Seq matched biosamples from MZ twins. Thus, we cannot address at present the question of how much of the genetic variation does contribute to the total percentage of ASE in MZ twins.

## Concluding Remarks

Our genome-wide scans for allelic expression discordance reveal an apparent state of pervasive missing allelic heritability in MZ co-twins. The extent and breadth of the ASE discordant sites are not exclusively associated with differences due to chromosomal aberrations and recombination, but also relate to the epigenome-wide differential allele expression phenomena of genomic imprinting and RNA editing. We conclude that most of ASE discordant sites observed within MZ pairs (either homokaryotypic or heterokaryotypic co-twins) cannot be attributed solely to the estimated within-pair incongruencies in DNA (Huang et al., 2019) or correspond to random transcriptional allelic noise varying across experiments (Chakravarti, 2011). The epigenome-wide ASE discordance may have essential effects on physiology, phenotype, or inheritance, and implications for the Developmental Origins of Health and Disease (DOHaD) approach in co-twins (Yamada and Chong, 2017).

## Web Resources

The URLs for public data used herein are as follows:

UCSC Genome Browser, https://genome.ucsc.edu/

GTEx portal, https://www.gtexportal.org/

NCBI SRA, https://www.ncbi.nlm.nih.gov/sra/

NCBI GEO, http://www.ncbi.nlm.nih.gov/geo/

dbGaP, http://www.ncbi.nlm.nih.gov/gap

PhenoScanner, http://www.phenoscanner.medschl.cam.ac.uk/phenoscanner

e-GRASP, http://www.mypeg.info/egrasp

HaploReg, http://compbio.mit.edu/HaploReg

PheGenI, https://www.ncbi.nlm.nih.gov/gap/phegeni

Geneimprint, http://www.geneimprint.com/

Metaimprint, http://bioinfo.hrbmu.edu.cn/MetaImprint/

dbMAE, https://mae.hms.harvard.edu/

OMIM, http://www.omim.org

RADAR, http://rnaedit.com/

Otago´s Catalogue of Imprinted Genes, http://igc.otago.ac.nz/

R software package, http://www.R-project.org

GATK, https://software.broadinstitute.org/gatk/

## Data Availability Statement

RNA-Seq data experiments used in this manuscript are from publicly available BioProjects. The primary data can be accessed from the NCBI SRA repository (https://www.ncbi.nlm.nih.gov/sra ) under the following run entries: SRR1182244, SRR1182246, SRR1182249, SRR1182248, SRR1182252, SRR1182253, SRR1182245, SRR1182247, SRR1182250, SRR1182251, SRR1182254, SRR1182255, SRR1028343, SRR1028344, SRR1028345, SRR1028346, SRR1028347, SRR1028348, SRR1028349, SRR519874, SRR519875, SRR519876, SRR519877, SRR519878, SRR519879, SRR519880, SRR519881, SRR519882, SRR519883, SRR519884, SRR519885, SRR519886, SRR519887, SRR519888, SRR519889, SRR519890, SRR519891, SRR3390461, SRR3390473, SRR3389246, SRR3390437.

## Author Contributions

RJ, CF, JS, DM, EM-A: Conceived and designed experiments. RJ, CF, JS, YC, VR, GC, EM-A: Develop scripts and carried computational analyses. JS, DM, AG: performed SRA RNA-Seq analysis for control SNPs queries. RJ, CF, JS, DM, AG, EM-A: Performed cross-reference of ASE sites. DM, RJ, CF, JS, EM-A: Prepared figures. EM-A: Supervised the work and drafted the manuscript. All authors provided substantial contributions to the interpretation of data,revised and approved the manuscript.

## Funding

This study was supported by grants from the Fundação de Amparo à Pesquisa do Estado do Rio de Janeiro - FAPERJ (BR) (http://www.faperj.br/) [grant number E26/010.001036/2015 to EM-A] and from the Conselho Nacional de Desenvolvimento Científico e Tecnológico - CNPq (BR) (http://cnpq.br/) [grant number 308780/2015-9 to EM-A]. JS and DM received undergraduate PIBIC/CNPq fellowships from the Universidade Estadual do Norte Fluminense Darcy Ribeiro UENF (BR) (http://www.uenf.br/). CF is a recipient of a graduate fellowship from the Fundação Coordenação de Aperfeiçoamento de Pessoal de Nível Superior - CAPES (http://www.capes.gov.br/). The agencies had no role in the study design, data collection, and analysis, decision to publish, or preparation of the manuscript.

## Conflict of Interest

The authors declare that the research was conducted in the absence of any commercial or financial relationships that could be construed as a potential conflict of interest.

## Supplementary Material

Dataset S1 RNA-Seq read counts and allele ratios supporting **Figure 1**.

Dataset S2 BioProjects and RNA-Seq experiments used in this study.

Dataset S3 ASE concordant and discordant sites identified in heterokaryotypic MZ twins assayed in fibroblasts and induced pluripotent stem cells (iPSC) derived from the co-twins discordant for trisomy 21 and maternal recombination at 21q.

Dataset S4 Summary of the average distribution of eSNVs per gene in the entire set of biosamples and heterogeneity scores across genes. **(A)** Summary of informative of eSNVs, **(B)** Summary of eSNVs by ASE profile. Ranking of genes by ASE heterogeneity/homogeneity scores in either heterokaryotypic **(C)** and homokaryotypic **(D)** twins.

Dataset S5 Concordant and discordant ASE sites identified in unrelated, non-twin males **(A)** and female **(B)** pairs assayed in peripheral mononuclear cells. Ranking of genes by ASE heterogeneity/homogeneity scores in unrelated, non-twin males and females **(C)**.

Datasets S6–S14 Concordant and discordant ASE sites identified in homokaryotypic MZ twins assayed in cultured B-cells derived from nine pairs of co-twins not discordant for a specific health condition.

Dataset S15 Cross-reference of ASE sites from the ten monozygotic twin pairs with allele expression profiles from secondary data of the GTEx project in multiple tissues.

Dataset S16 Summary of A-to-I(G) RNA-editing sites resulting in synonymous and non-synonymous substitutions in the ten twin pairs set.

Dataset S17 Protein-coding genetic eSNVs associated with disease risk or disease pathogenesis in the ten twin pairs set.

Dataset S18 Gene ontology enrichment annotations.

Table S1 Review of cases of heterokaryotypic co-twins reported in the literature.

Table S2 Distribution of ASE sites by gene structure sequence context. Cross-reference of ASE sites disparities observed in the present study with the annotation features of the eSNV disparities found in the whole-genome sequencing study by Huang et al. 2019.

Table S3 ASE disparities in X-linked genes subject to XCI between MZ twins.

Table S4 Evidence for allele-specific expression form of mitochondrial microheteroplasmy in MZ twin pairs.

Figure S1 Representation of the discordant maternal 21q inheritance in the pair of co-twins heterokaryotypic for trisomy 21 reported by Dahoun et al. (2008). The MZ co-twins are discordant for trisomy 21 of maternal origin in twin 1 (T1DS) and maternal allelic disparity at 21qter likely carried by disomic twin 2 (T2N). The discordant inheritance of 21q is probably due to meiosis I subtelomeric recombination event likely occurring between the maternal chromosomes 21 within the 1.7Mb interval (hg38) delimited by the short tandem repeat marker D21S1445, where alleles were identical in both twins and the short tandem repeat marker D21S1611, where different alleles were inherited.

Figure S2 Flowchart of analysis. The in-house computational pipeline, PipASE, used for scanning and sorting out genome-wide, allele-specific differences between MZ co-twins.

Figure S3 Chromosomal distribution of eSNVs in iPSC. **(A)** Genome-wide e-karyotyping for the SNVs exhibiting allele-specific expression in iPSC derived from the co-twins discordant for T21 and maternal recombination at 21q. Shown is the distribution of all ASE sites that were concordant (gray ticks towards the left of each chromosome ideogram) or discordant (blue ticks towards the right side). **(B)** Assessment of chromosomal aberrations by e-karyotyping allelic bias using RNA-Seq data from iPSC in **(A)**.

Figure S4 Overview of the breadth and magnitude of allele-specific expression disparity between nine MZ control twin pairs. The RNA-Seq SRA entries for the nine twin pairs are SRR519874, SRR519875, SRR519876, SRR519877, SRR519878, SRR519879, SRR519880, SRR519881, SRR519882, SRR519883, SRR519884, SRR519885, SRR519886, SRR519887, SRR519888, SRR519889, SRR519890, and SRR519891. For each SRA entry above, the panels are **(A)** numbers of ASE sites distributed by the within-pair status of concordance or discordance in control homokaryotypic MZ control twins tested in cultured B-cells. The majority of ASE sites are concordant for a biallelic imbalance status. On average, the co-twins are discordant in 1074 ± 252.03 ASE sites. **(B)** Comparison of the effect size of LogASE. We calculated the log2 of allele-specific expression fold change using the equation LogASE = log2(T1_ASE / T2_ASE) for each eSNV in each tissue. LogASE estimates the magnitude of expression change between conditions for the variant. **(C)** Distribution of genes by numbers of ASE sites observed in cultured B-cells.

Figure S5 Chromosomal distribution of eSNVs in the homokaryotypic twin pairs. Genome-wide e-karyotyping for the SNVs exhibiting allele-specific expression in cultured B-cells from nine control twin pairs. Shown in each panel,**(A)** through **(I)**, is the distribution of all ASE sites that were concordant (gray ticks towards the left of each chromosome ideogram) or discordant (red ticks towards the right side). The RNA-Seq SRA entries for the nine twin pairs in are SRR519874, SRR519875, SRR519876, SRR519877, SRR519878, SRR519879, SRR519880, SRR519881, SRR519882, SRR519883, SRR519884, SRR519885, SRR519886, SRR519887, SRR519888, SRR519889, SRR519890, and SRR519891, respectively.

Figure S6 Assessment of chromosomal aberrations by e-karyotyping allelic bias using RNA-Seq data from control twin pairs. (A) through (I). The RNA-Seq SRA entries for the nine twin pairs used as controls are SRR519874, SRR519875, SRR519876, SRR519877, SRR519878, SRR519879, SRR519880, SRR519881, SRR519882, SRR519883, SRR519884, SRR519885, SRR519886, SRR519887, SRR519888, SRR519889, SRR519890, and SRR519891, respectively. For each SRA entry above, a plot is shown, which represents the distribution of allele ratios in cultured B-cells from nine pairs of co-twins who are not discordant for a specific health condition. None of the control twin pairs present detectable chromosomal aberrations.

Figure S7 Within twin-pair disparities in allele expression proportions at eSNVs that are coincident with canonical A-to-I(G) RNA editing sites in homokaryotypic twins. Shown is the distribution of eSNVs that positionally match canonical RNA editing sites between nine homokaryotypic co-twins, **(A)** through **(I)**, assayed in culture-B-cells. Each dot corresponds to an eSNV. The vast majority of sites exhibited a concordant biallelic imbalance profile (pink and light blue dots). Red dots represent eSNPs that were discordant between co-twins in that they exhibited allelic proportions differences higher than 25%, regardless of the discordance or concordance in the karyotype. Green dots represent eSNVs that exhibited discordant allelic profiles, being biallelic in one twin and monoallelic in the other. The linear models (solid black lines), the confidence interval of the models (broken purple lines) and of the prediction (solid purple lines) were constructed using R. Model equations: **(A)** Y = 8.15439 + 0.82362X; **(B)** Y = 7.20067 + 0.80502X; **(C)** Y = 6.0758 + 0.8080X; **(D)** Y = 12.74607 + 0.78267X; **(E)** Y = 14.78510 + 0.79019X; **(F)** Y = 5.64298 + 0.81255X; **(G)** Y = 7.97718 + 0.82152X; **(H)** Y = 8.36441 + 0.81231X; **(I)** Y = 6.60496 + 0.72912X. For all pairs, P < 2.2e-16. The RNA-Seq SRA entries for the nine twin pairs used as controls are SRR519874, SRR519875, SRR519876, SRR519877, SRR519878, SRR519879, SRR519880, SRR519881, SRR519882, SRR519883, SRR519884, SRR519885, SRR519886, SRR519887, SRR519888, SRR519889, SRR519890, and SRR519891.
