# Supplementary figures and images for: Pervasive Inter-Individual Variation in Allele-Specific Expression in Monozygotic Twins
Source: Front Genet. 2019 Nov 26;10:1178. doi: 10.3389/fgene.2019.01178 (PMC6887657; doi:10.3389/fgene.2019.01178)

Figure S1

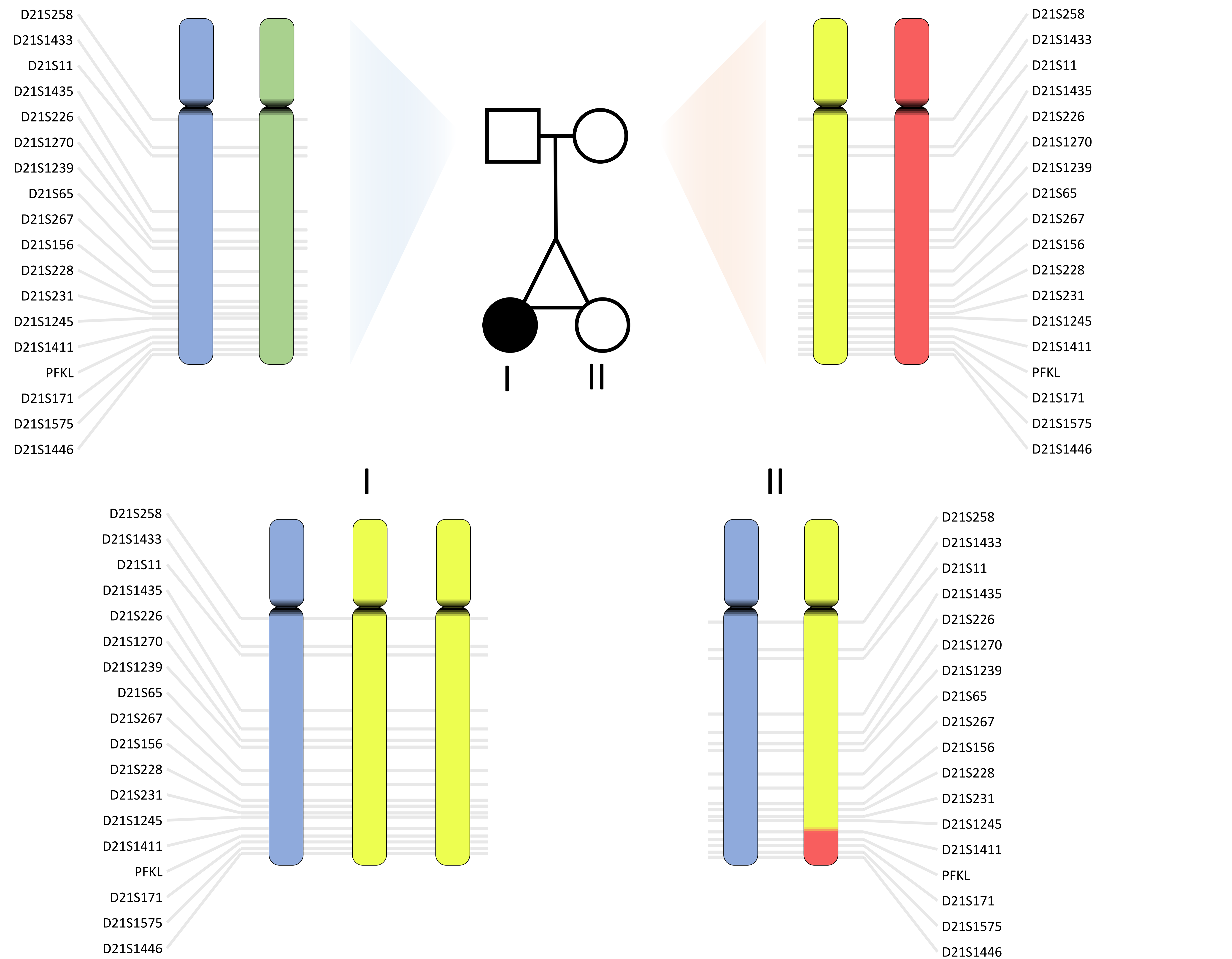

Supplement: Figure S1 — Representation of the discordant maternal 21q inheritance in the pair of co-twins heterokaryotypic for trisomy 21 reported by Dahoun et al. (2008). The MZ co-twins are discordant for trisomy 21 of maternal origin in twin 1 (T1DS) and maternal allelic disparity at 21qter likely carried by disomic twin 2 (T2N). The discordant inheritance of 21q is probably due to meiosis I subtelomeric recombination event likely occurring between the maternal chromosomes 21 within the 1.7Mb interval (hg38) delimited by the short tandem repeat marker D21S1445, where alleles were identical in both twins and the short tandem repeat marker D21S1611, where different alleles were inherited. [file Image_1.pdf]

**Figure S2**

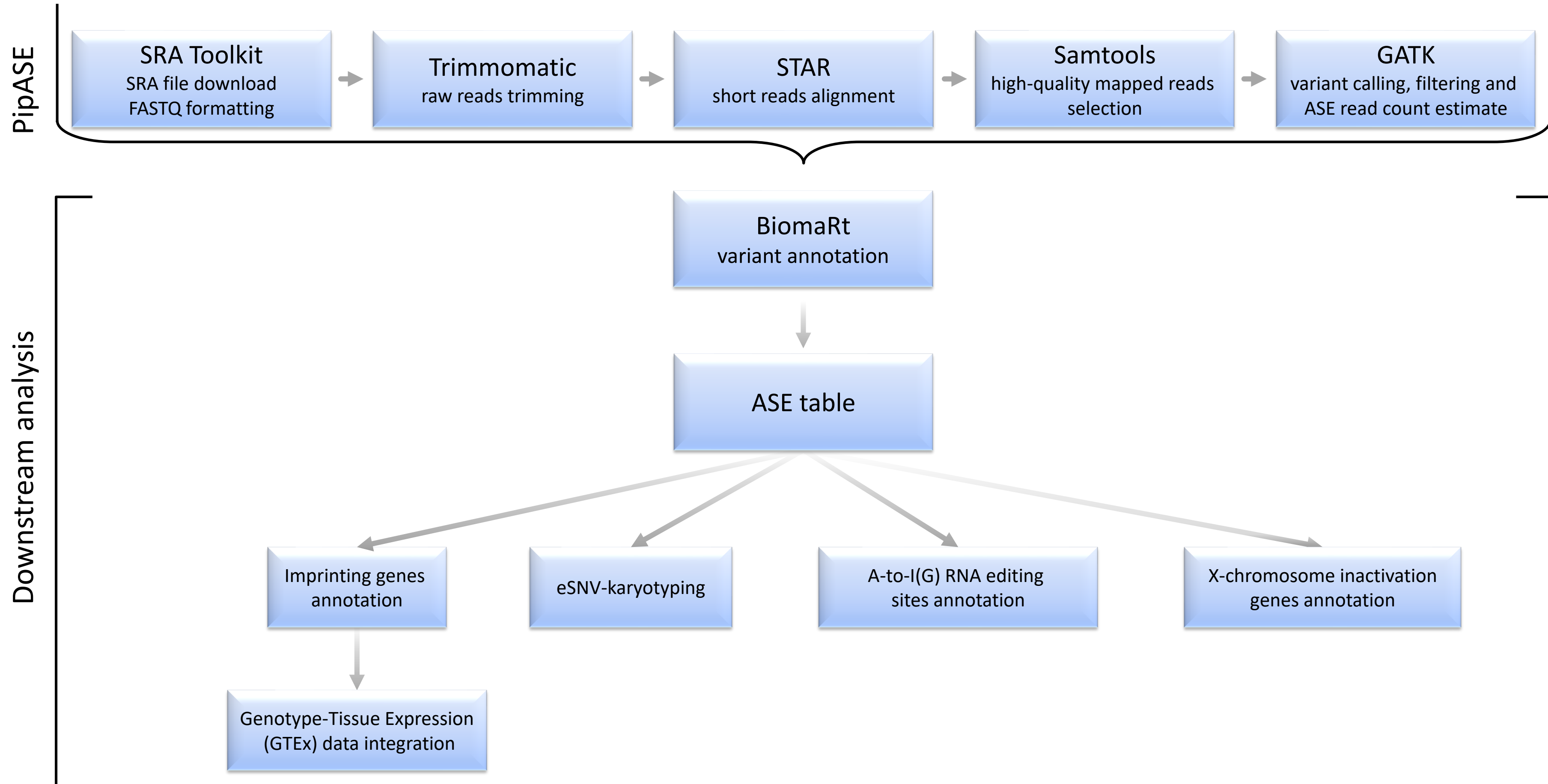

Supplement: Figure S2 — Flowchart of analysis. The in-house computational pipeline, PipASE, used for scanning and sorting out genome-wide, allele-specific differences between MZ co-twins. [file Image_2.pdf]

Figure S3

A

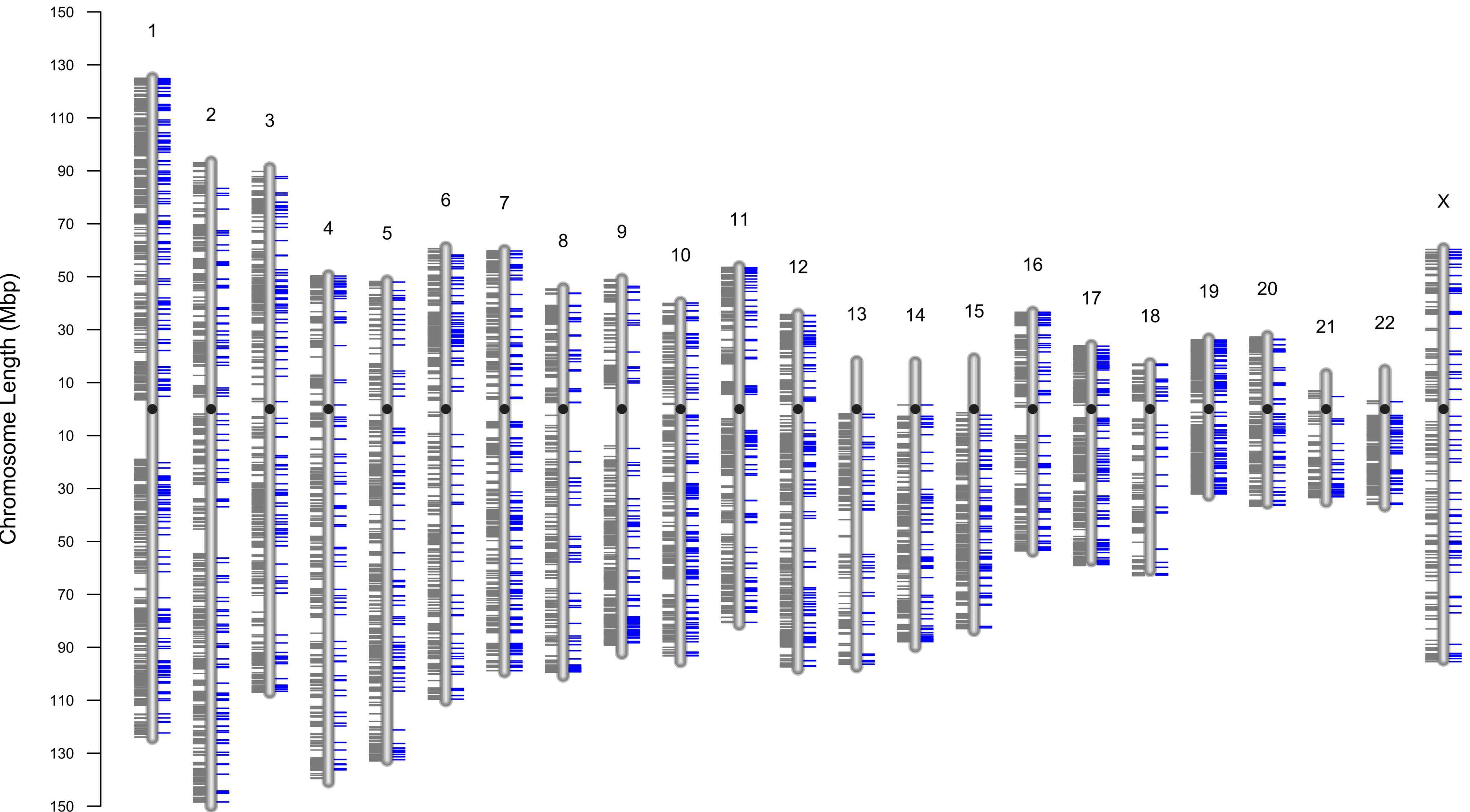

B

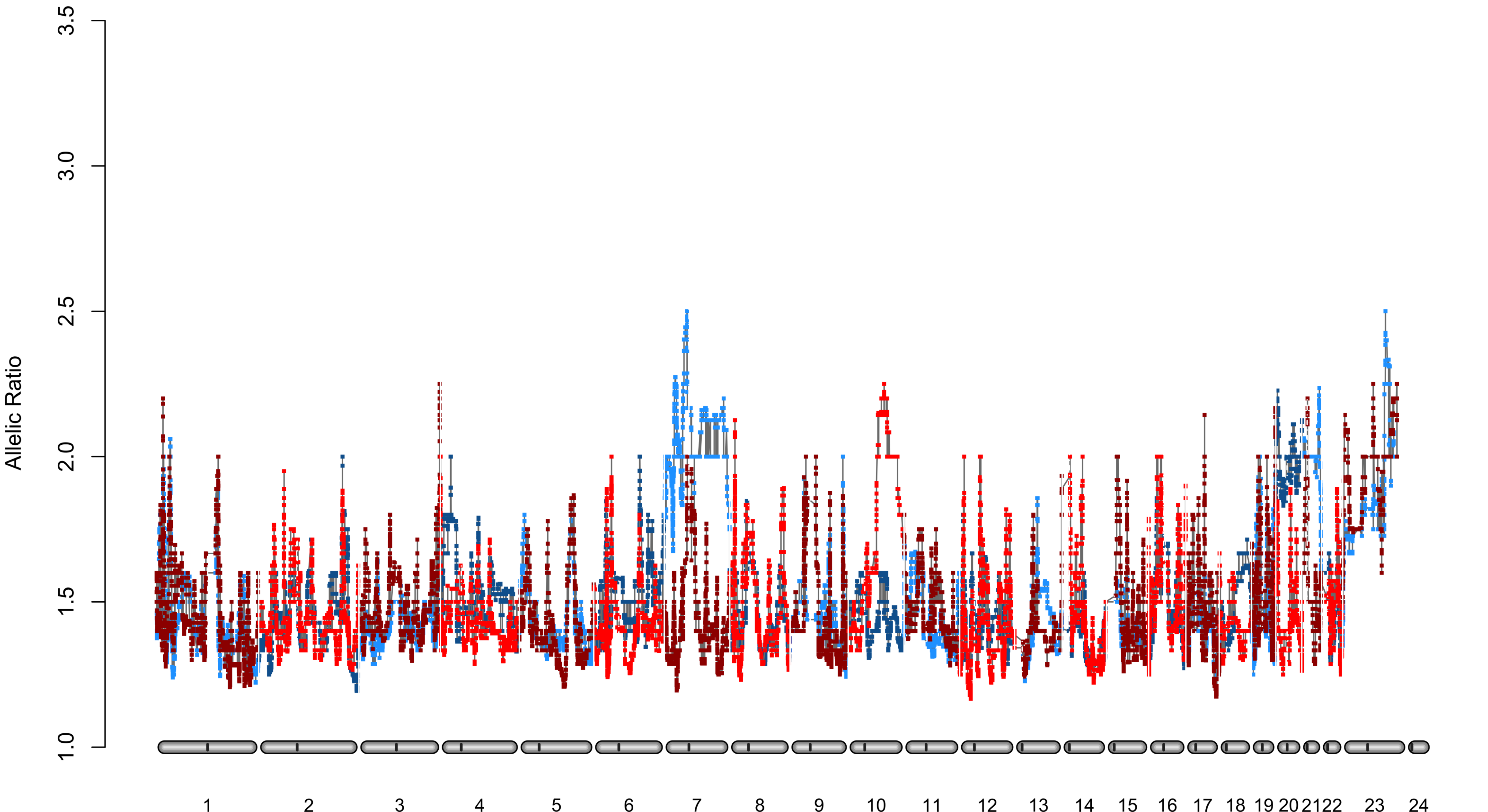

Supplement: Figure S3 — Chromosomal distribution of eSNVs in iPSC. (A) Genome-wide e-karyotyping for the SNVs exhibiting allele-specific expression in iPSC derived from the co-twins discordant for T21 and maternal recombination at 21q. Shown is the distribution of all ASE sites that were concordant (gray ticks towards the left of each chromosome ideogram) or discordant (blue ticks towards the right side). (B) Assessment of chromosomal aberrations by e-karyotyping allelic bias using RNA-Seq data from iPSC in (A). [file Image_3.pdf]

**Figure S6 (A)**

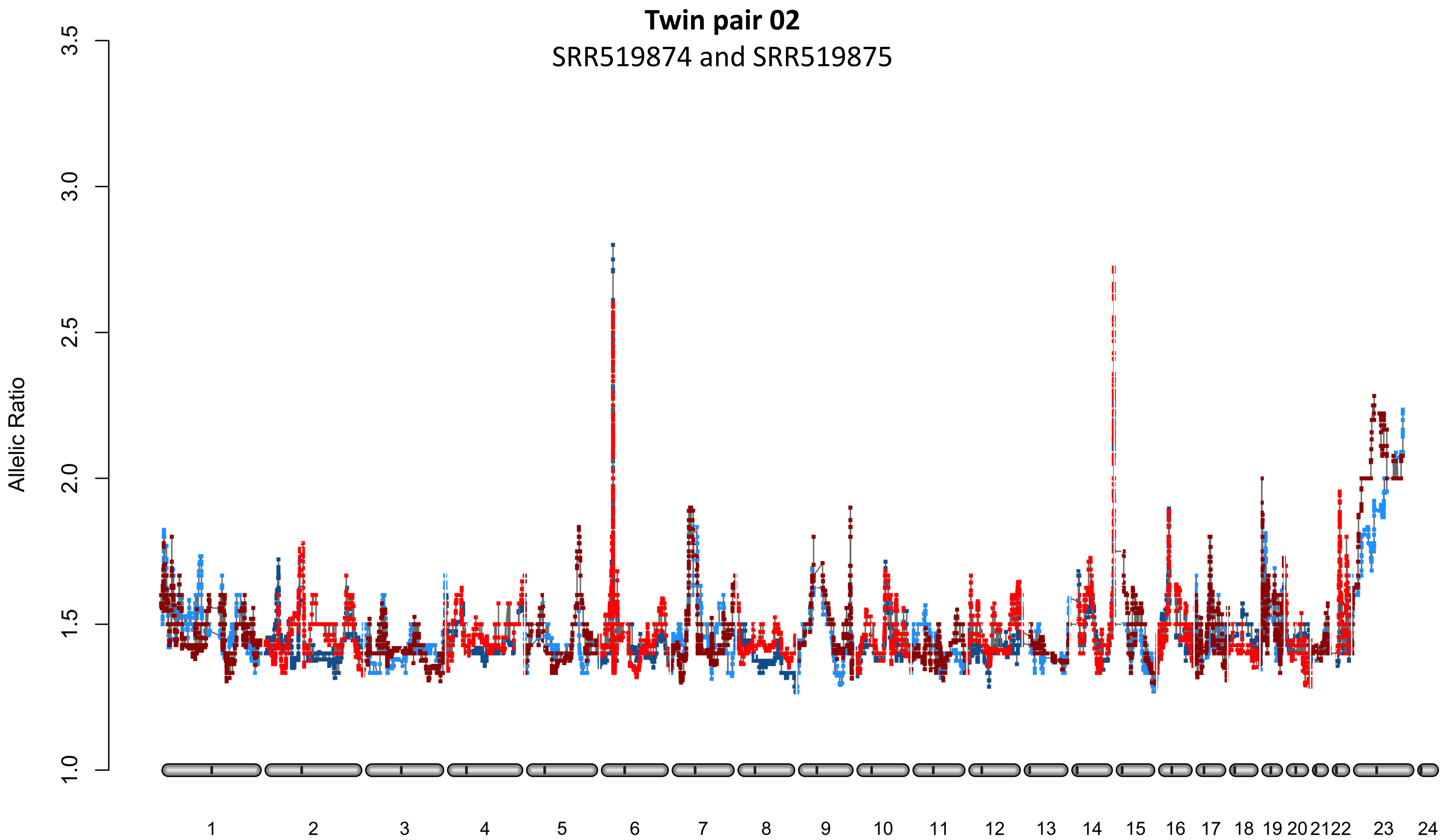

**Figure S6 (B)**

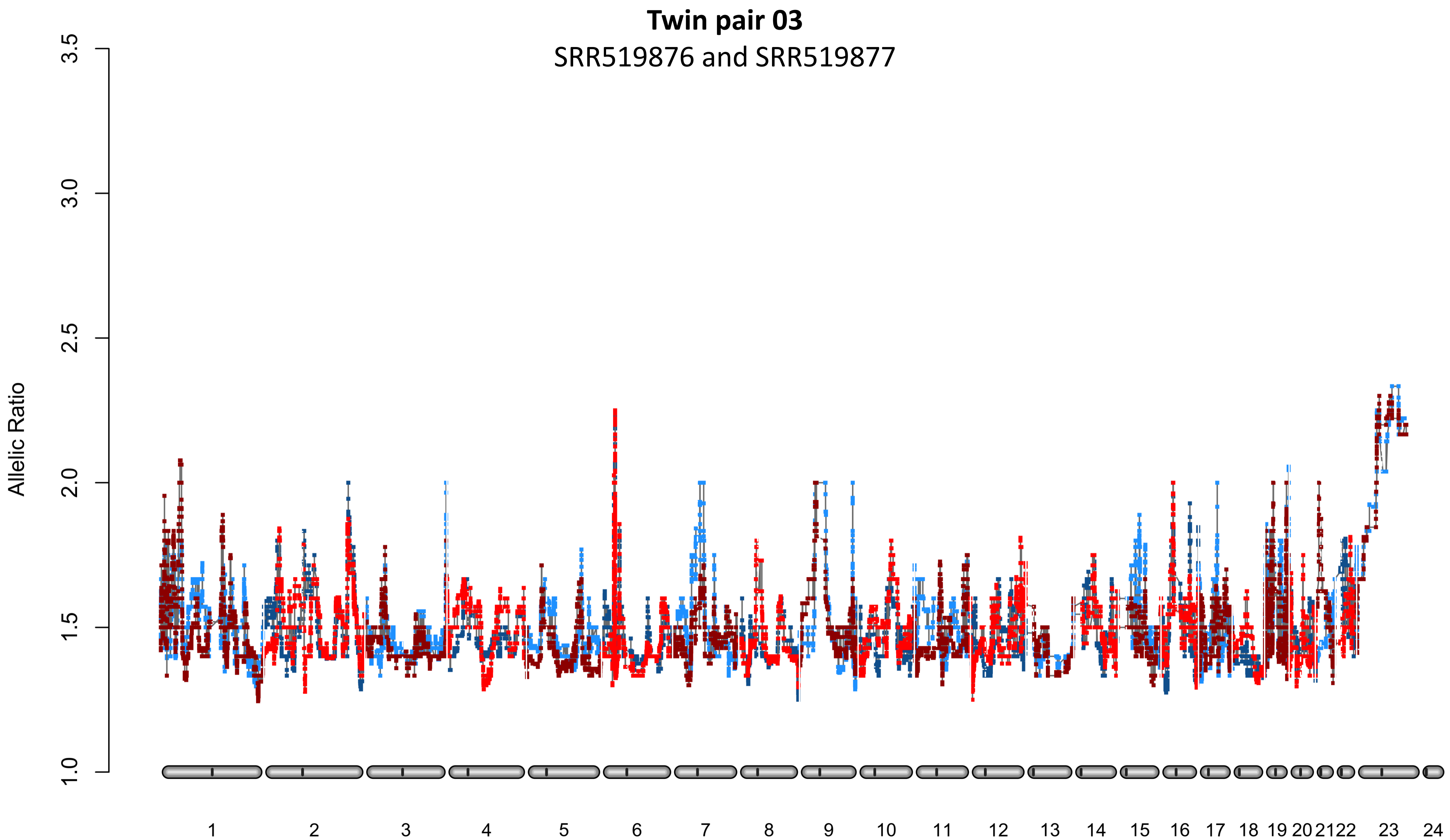

**Figure S6 (C)**

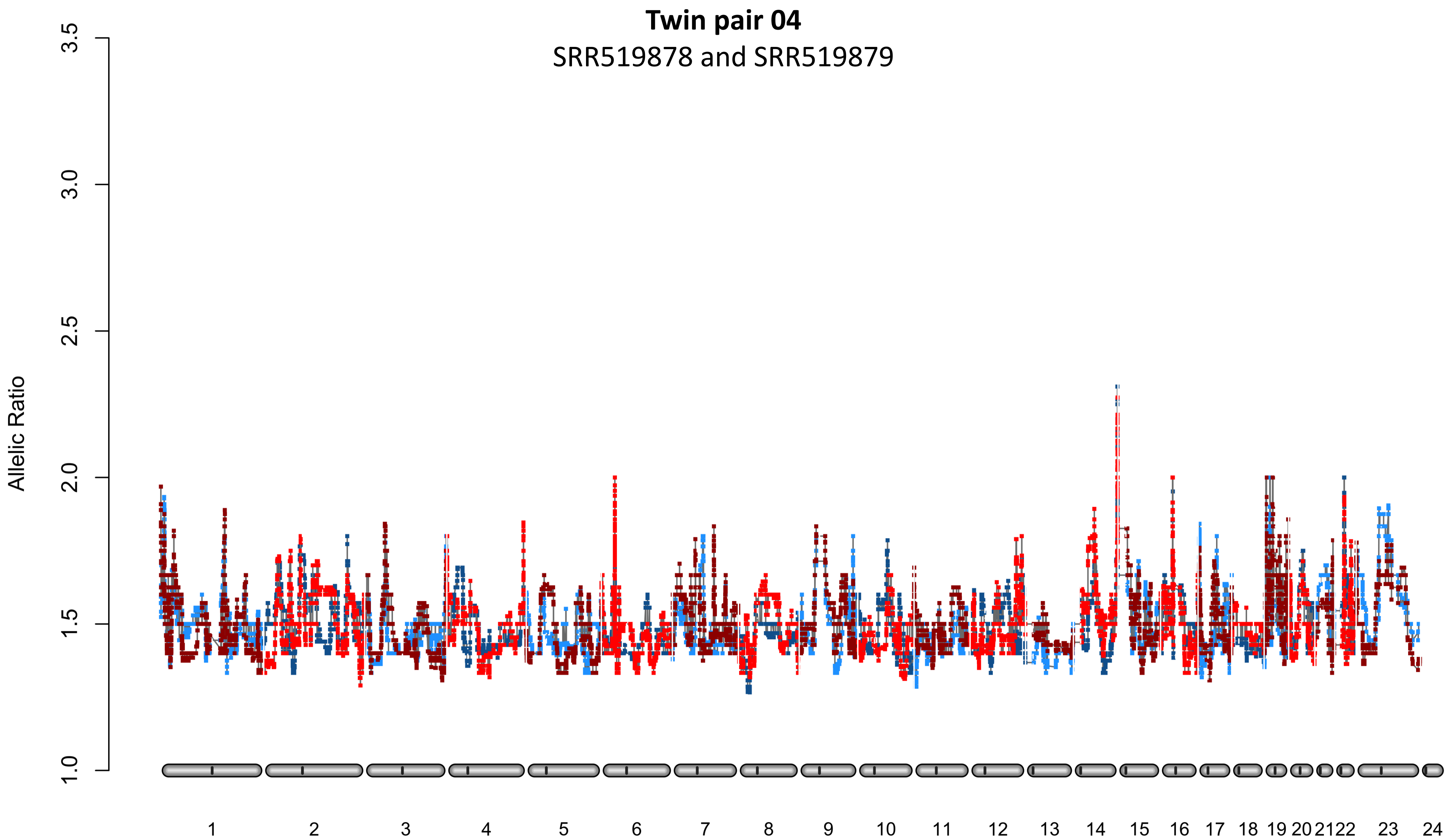

**Figure S6 (D)**

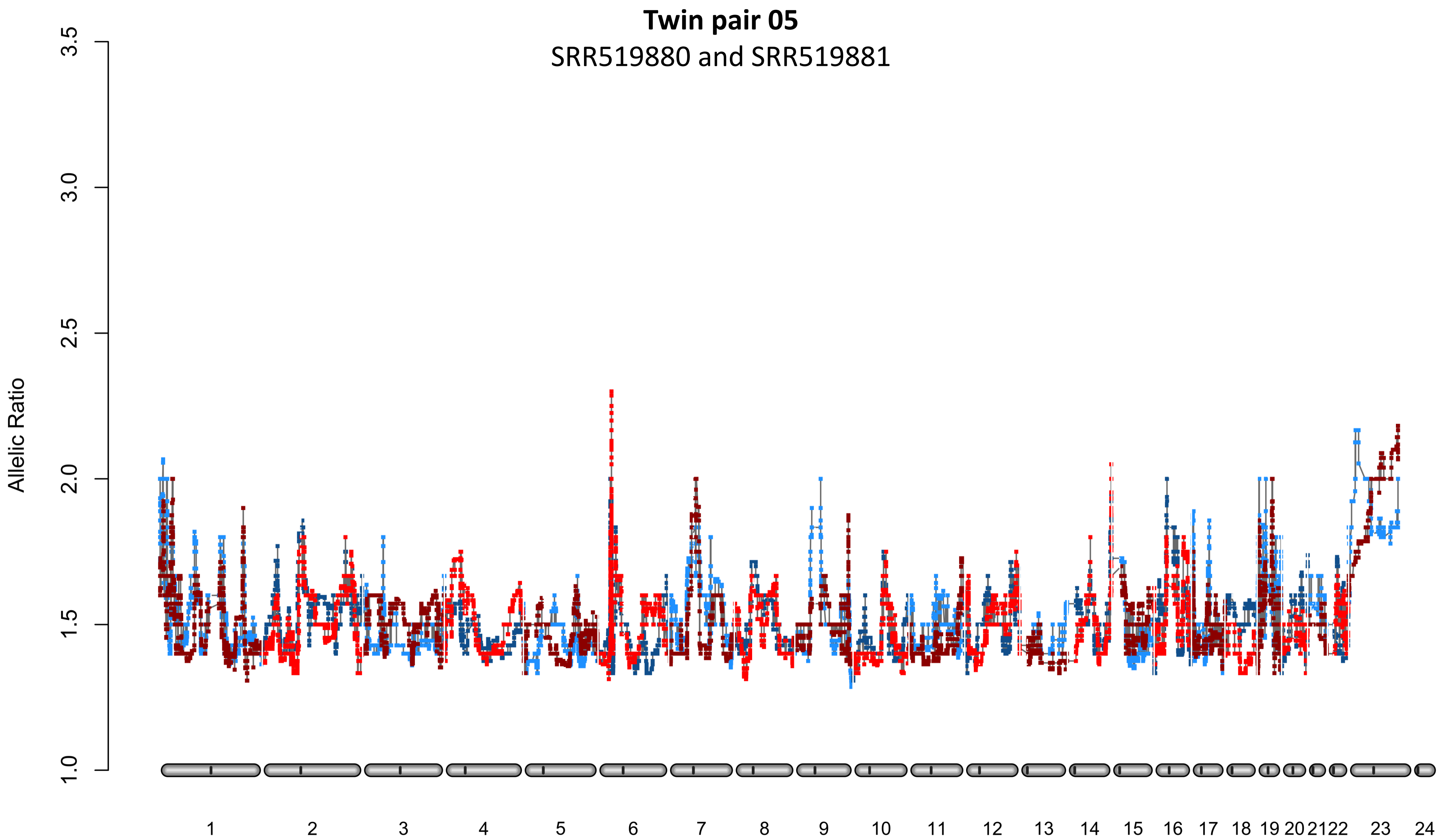

**Figure S6 (E)**

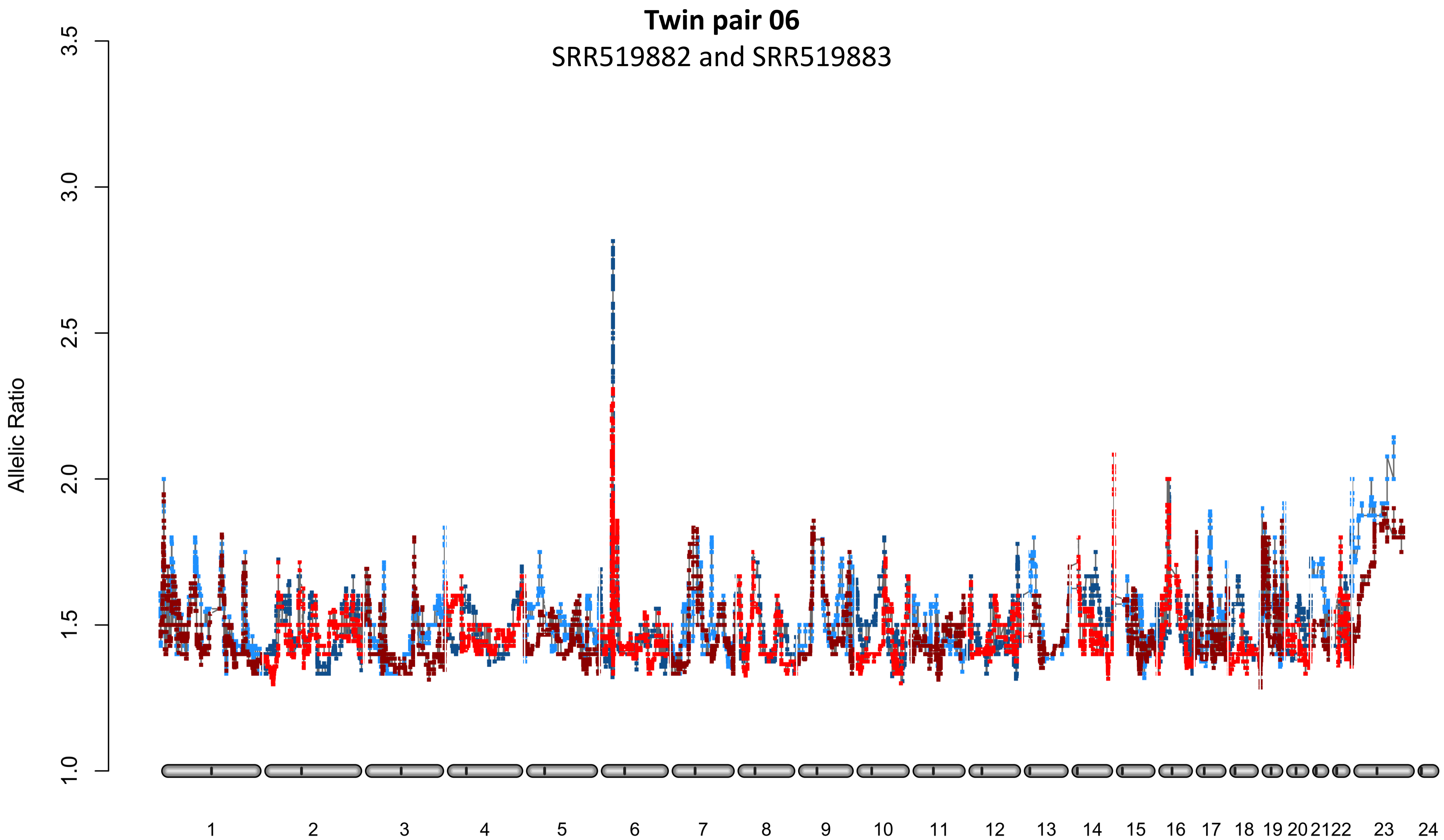

**Figure S6 (F)**

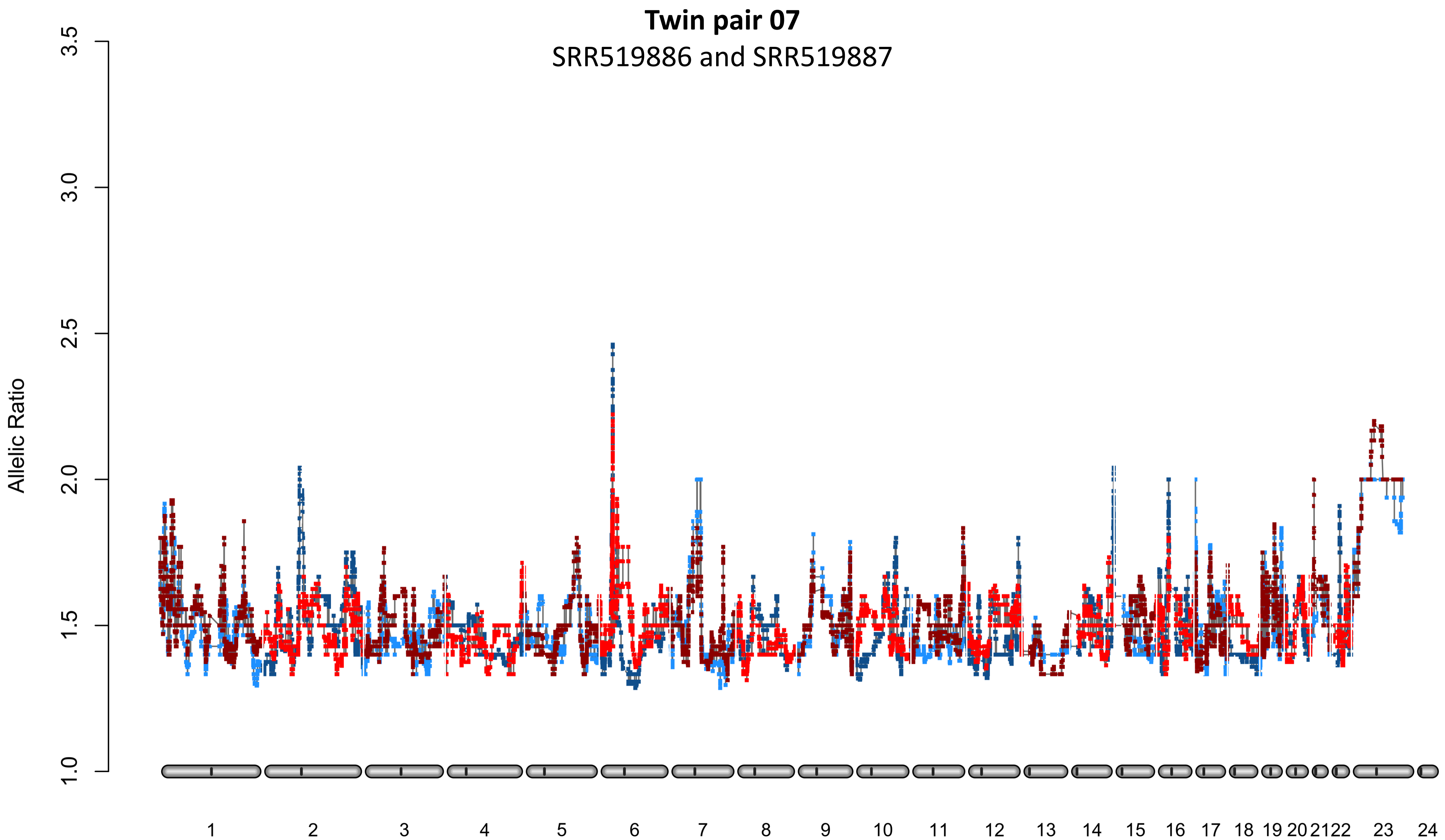

**Figure S6 (G)**

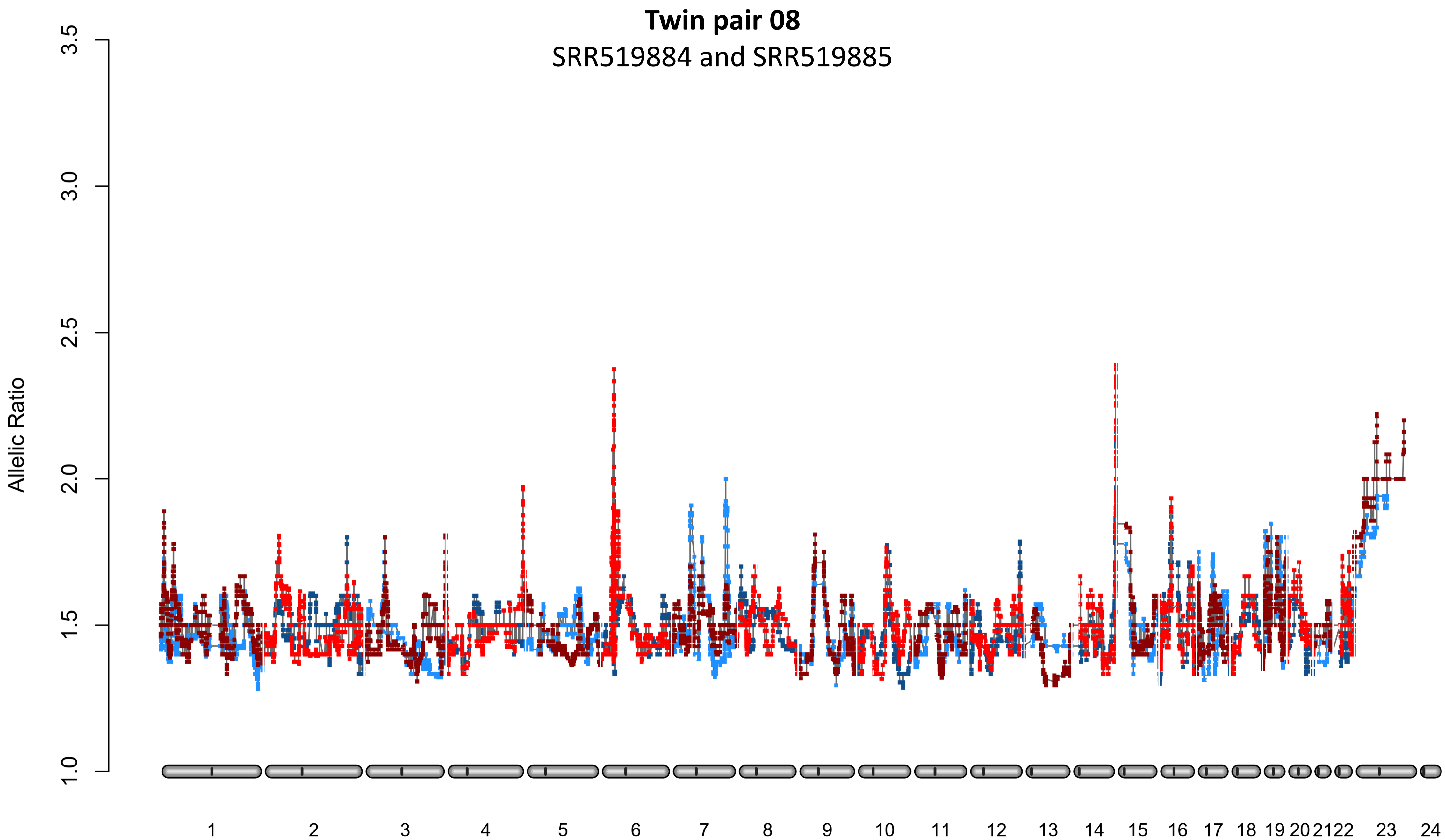

**Figure S6 (H)**

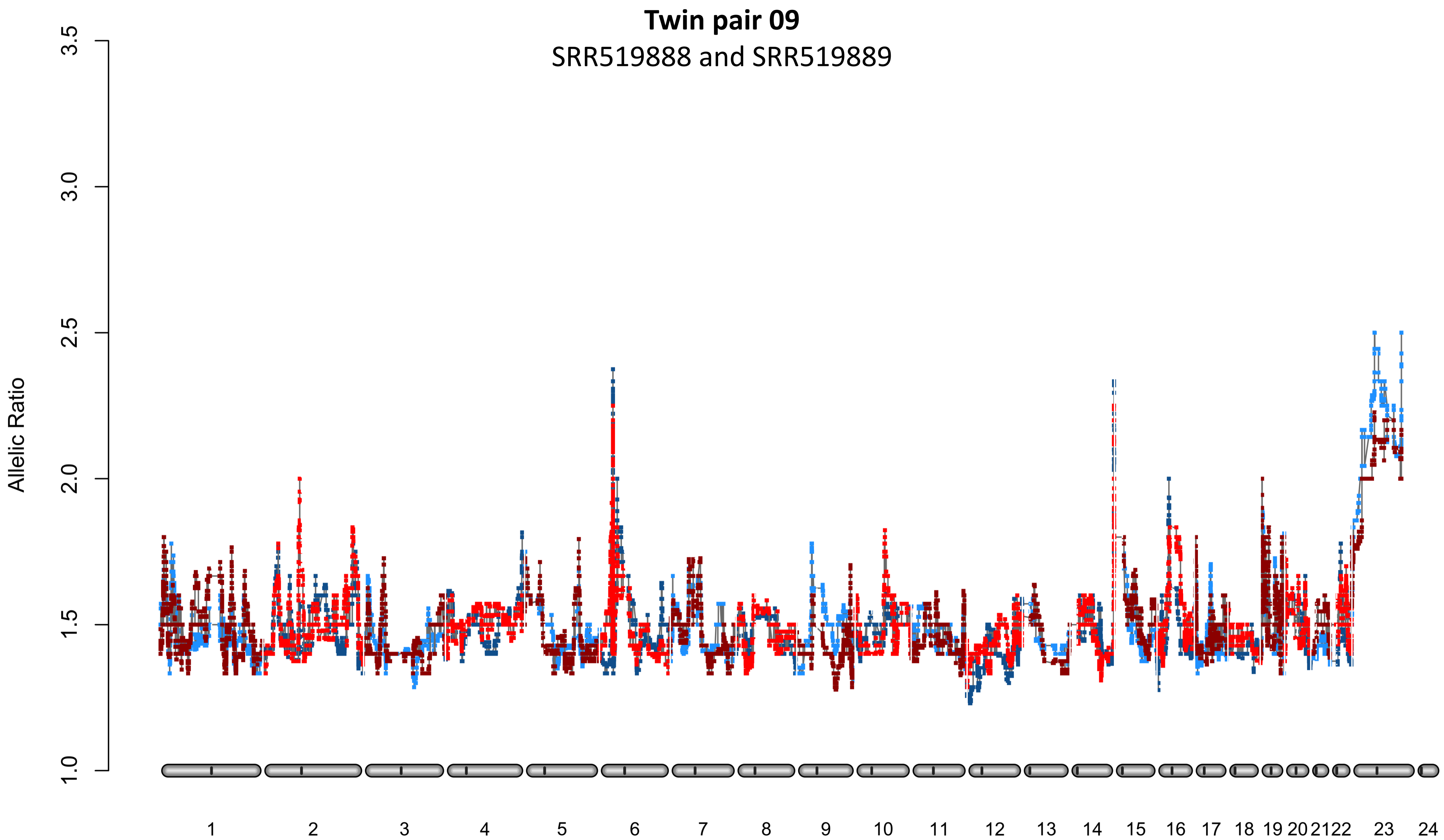

**Figure S6 (I)**

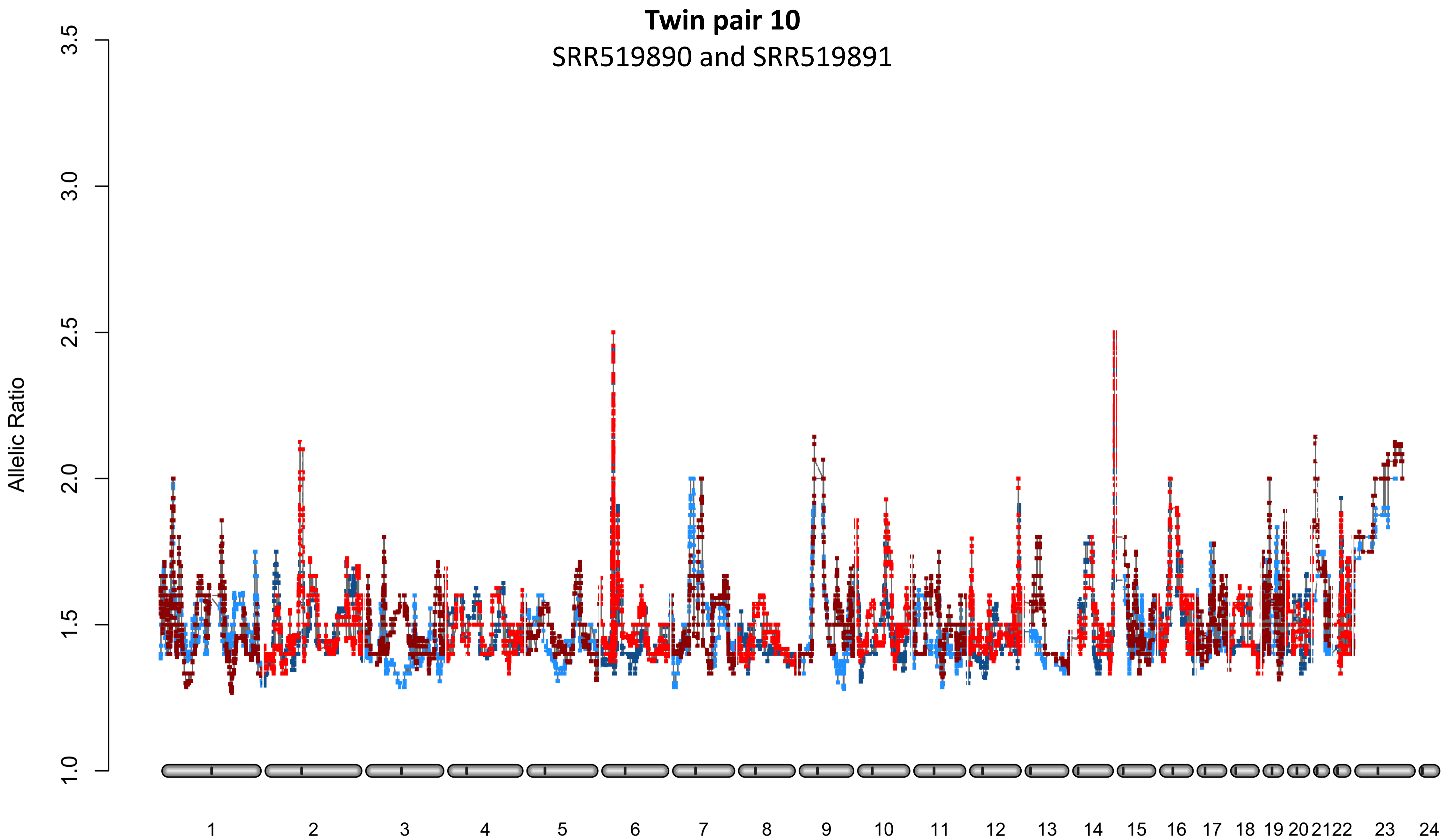

Supplement: Figure S6 — Assessment of chromosomal aberrations by e-karyotyping allelic bias using RNA-Seq data from control twin pairs. (A) through (I). The RNA-Seq SRA entries for the nine twin pairs used as controls are SRR519874, SRR519875, SRR519876, SRR519877, SRR519878, SRR519879, SRR519880, SRR519881, SRR519882, SRR519883, SRR519884, SRR519885, SRR519886, SRR519887, SRR519888, SRR519889, SRR519890, and SRR519891, respectively. For each SRA entry above, a plot is shown, which represents the distribution of allele ratios in cultured B-cells from nine pairs of co-twins who are not discordant for a specific health condition. None of the control twin pairs present detectable chromosomal aberrations. [file Image_6.pdf]

Figure S7

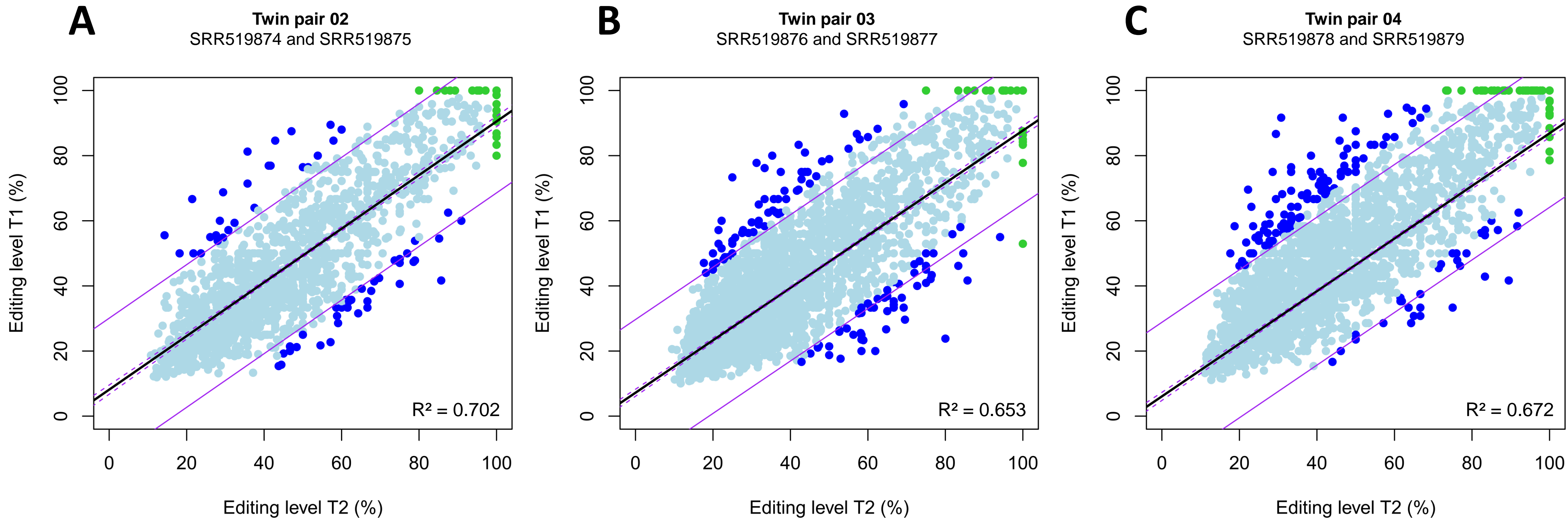

Figure S7

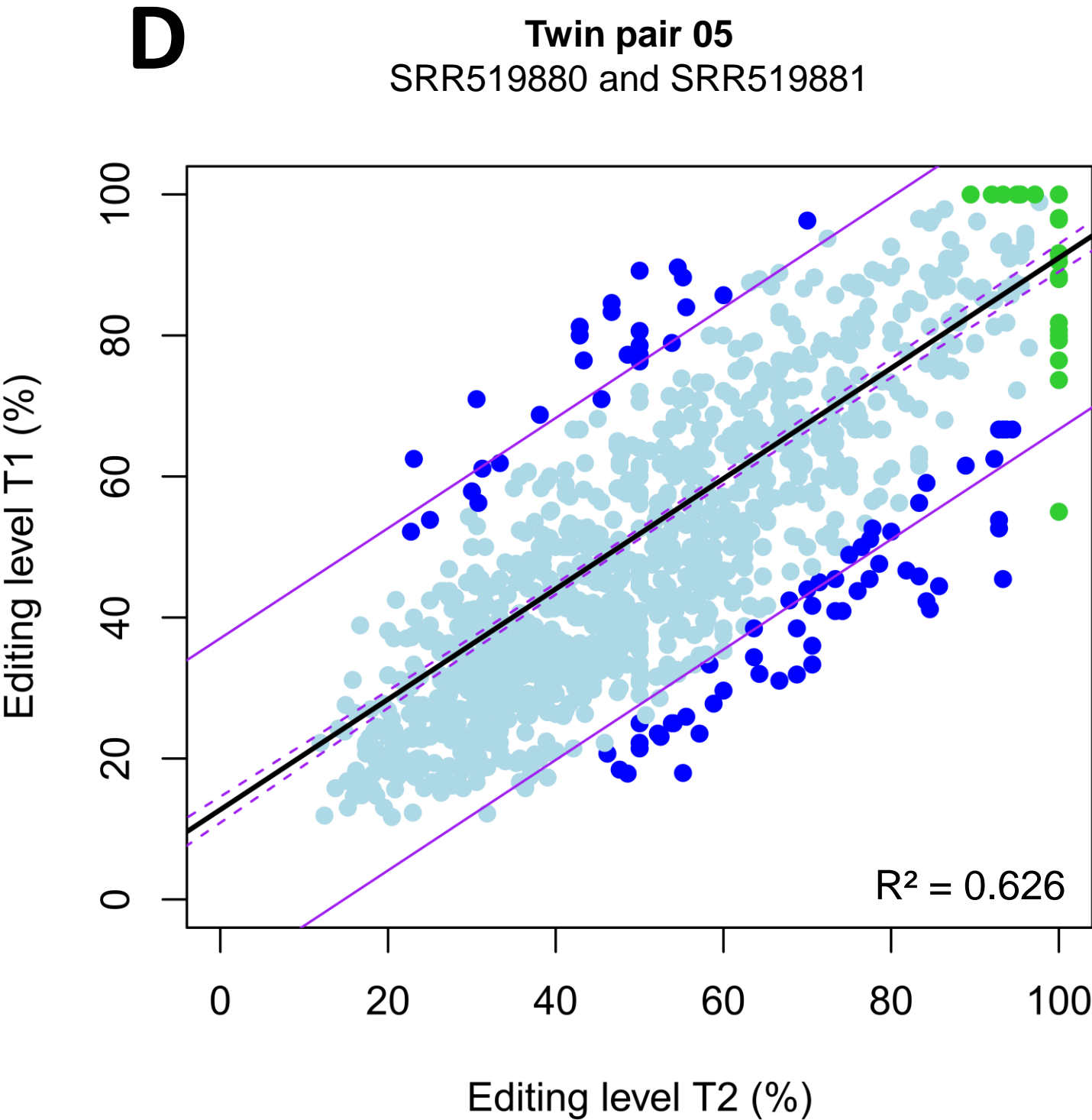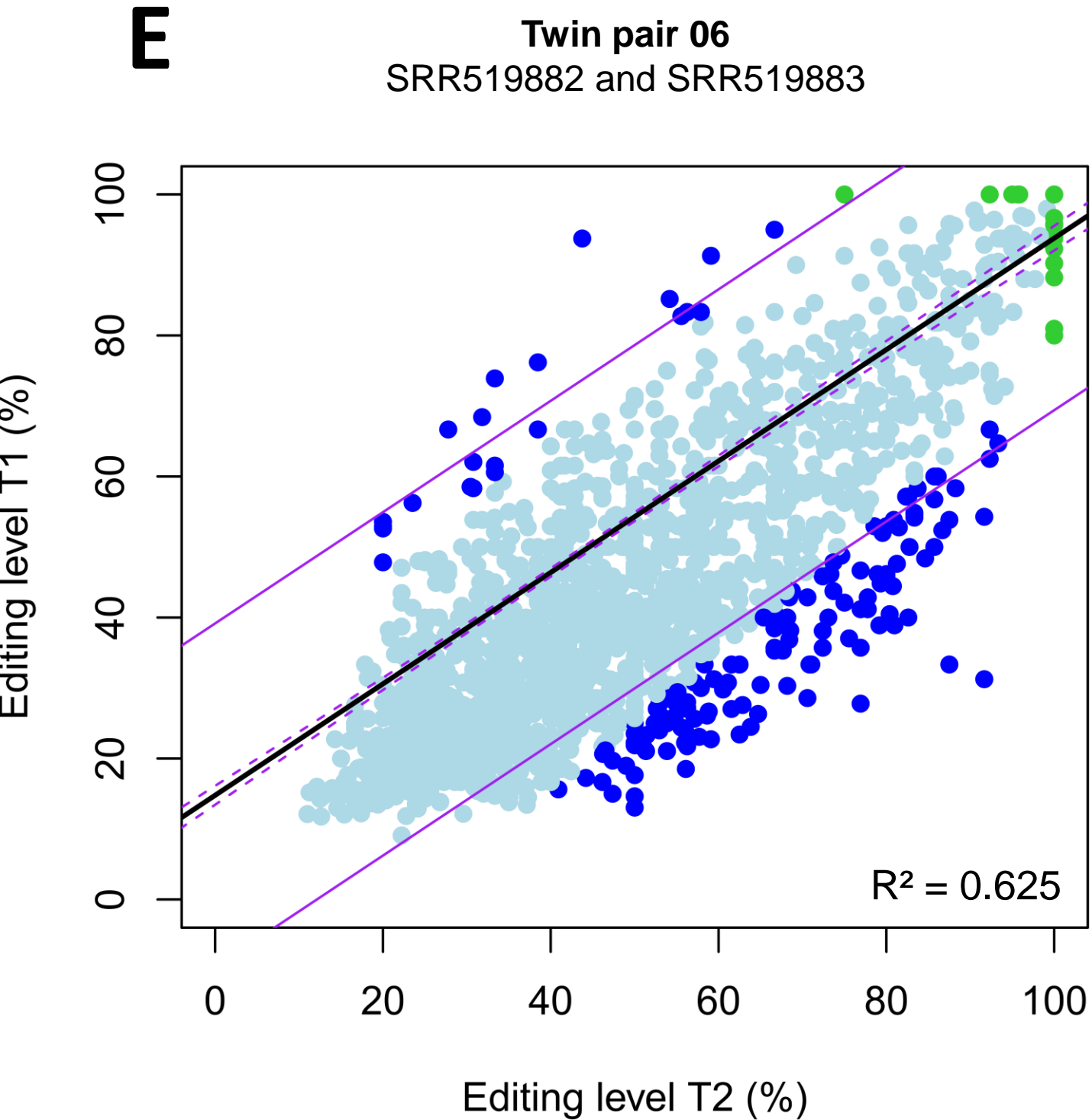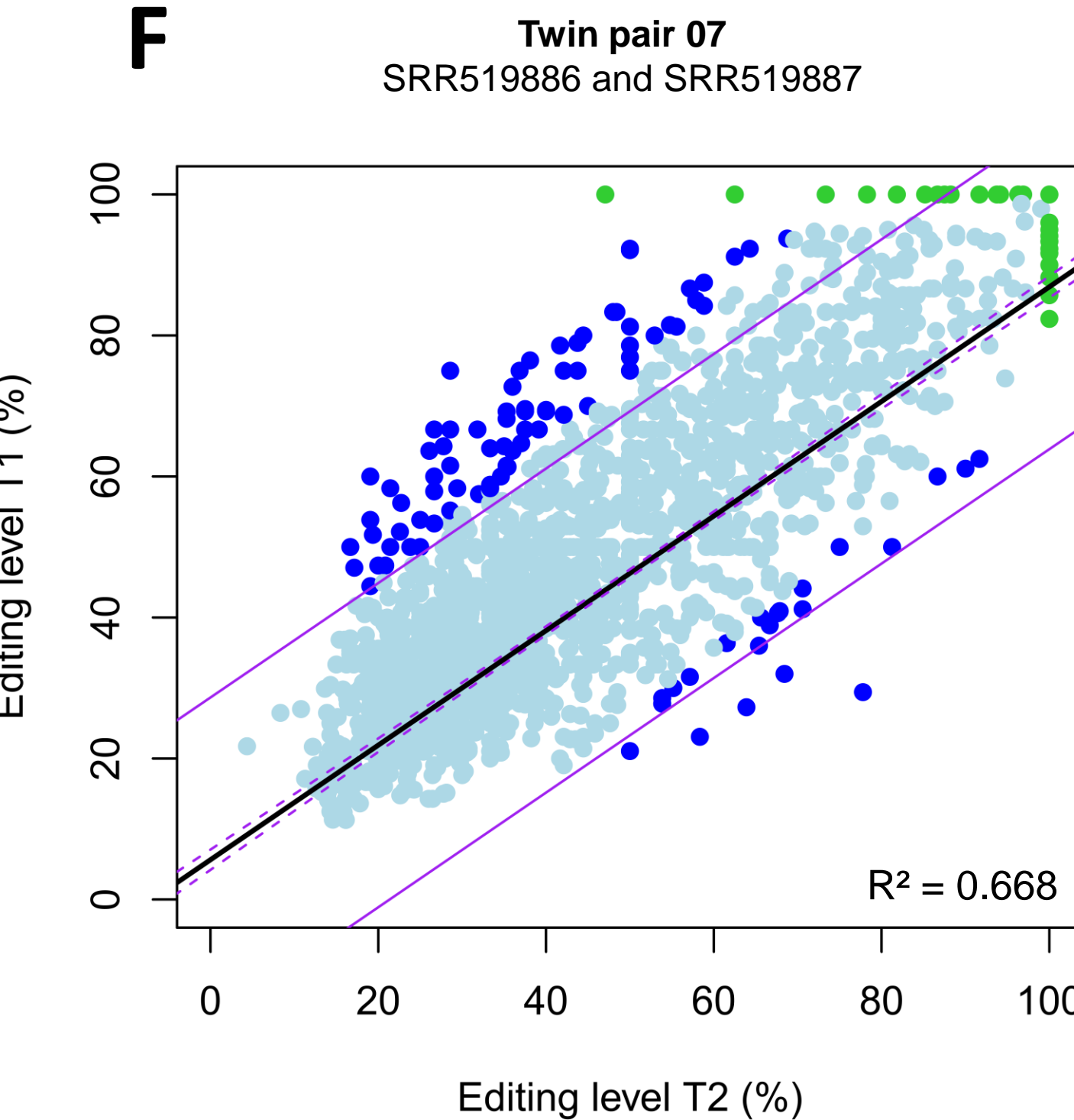

Figure S7

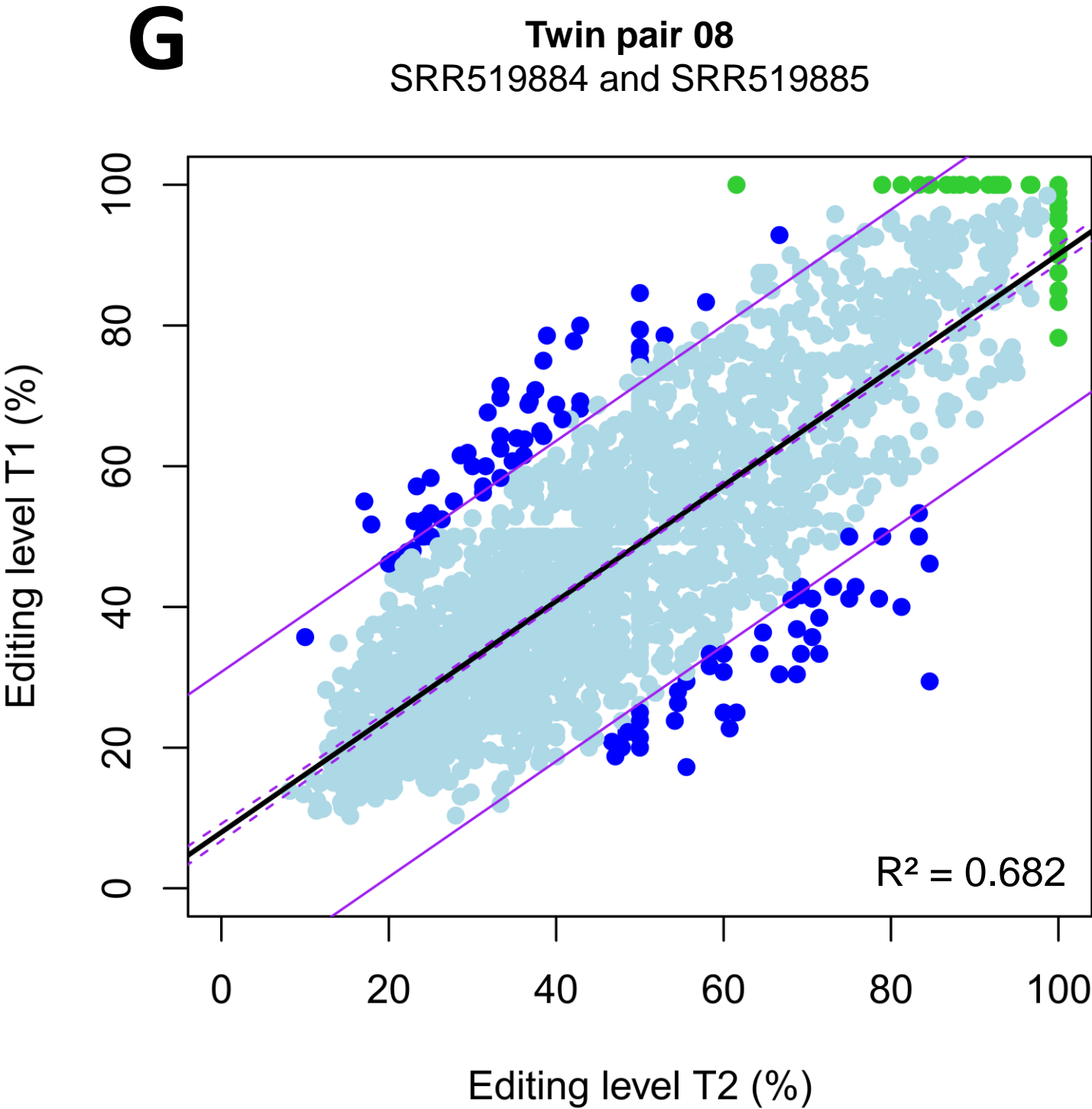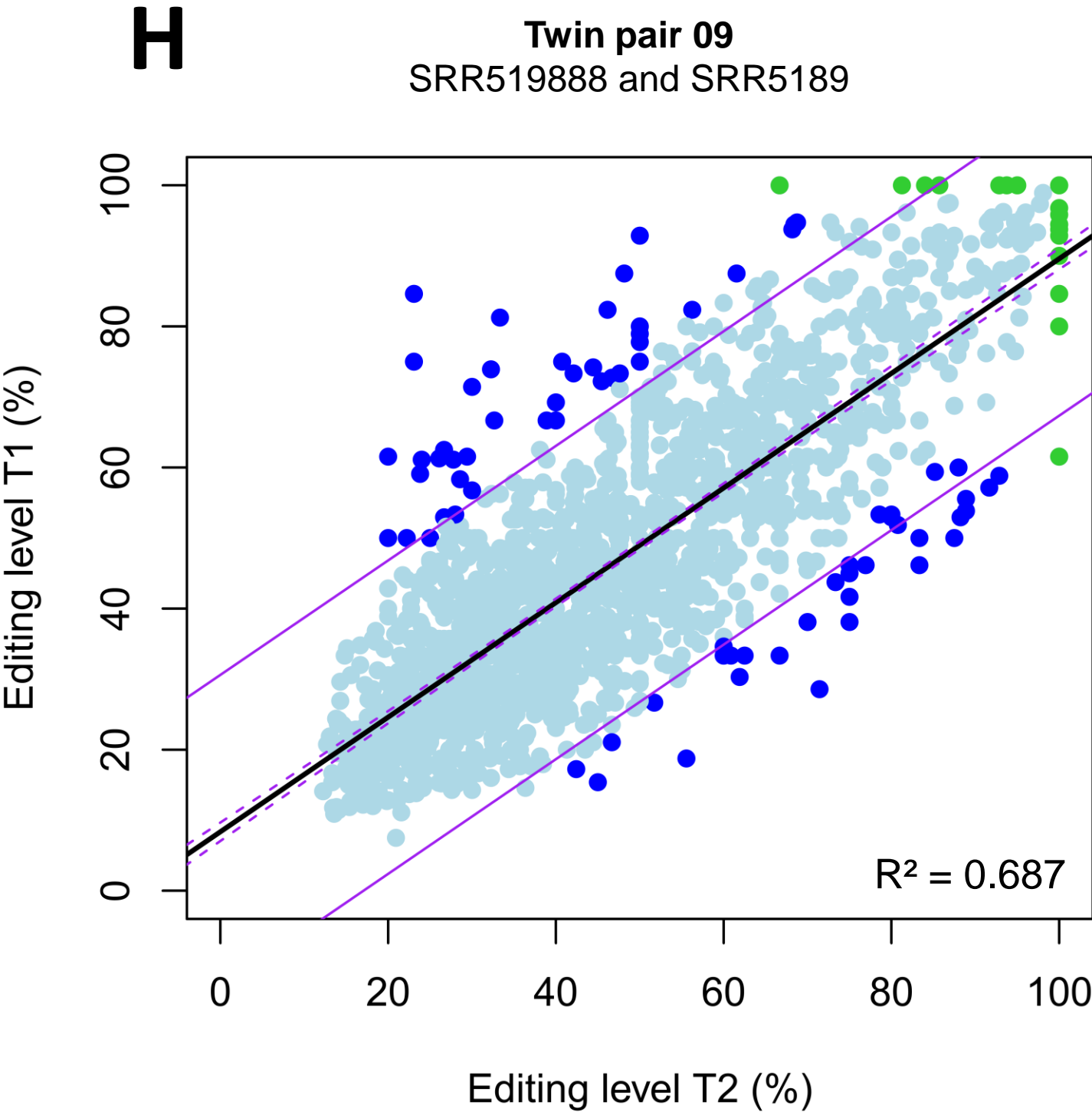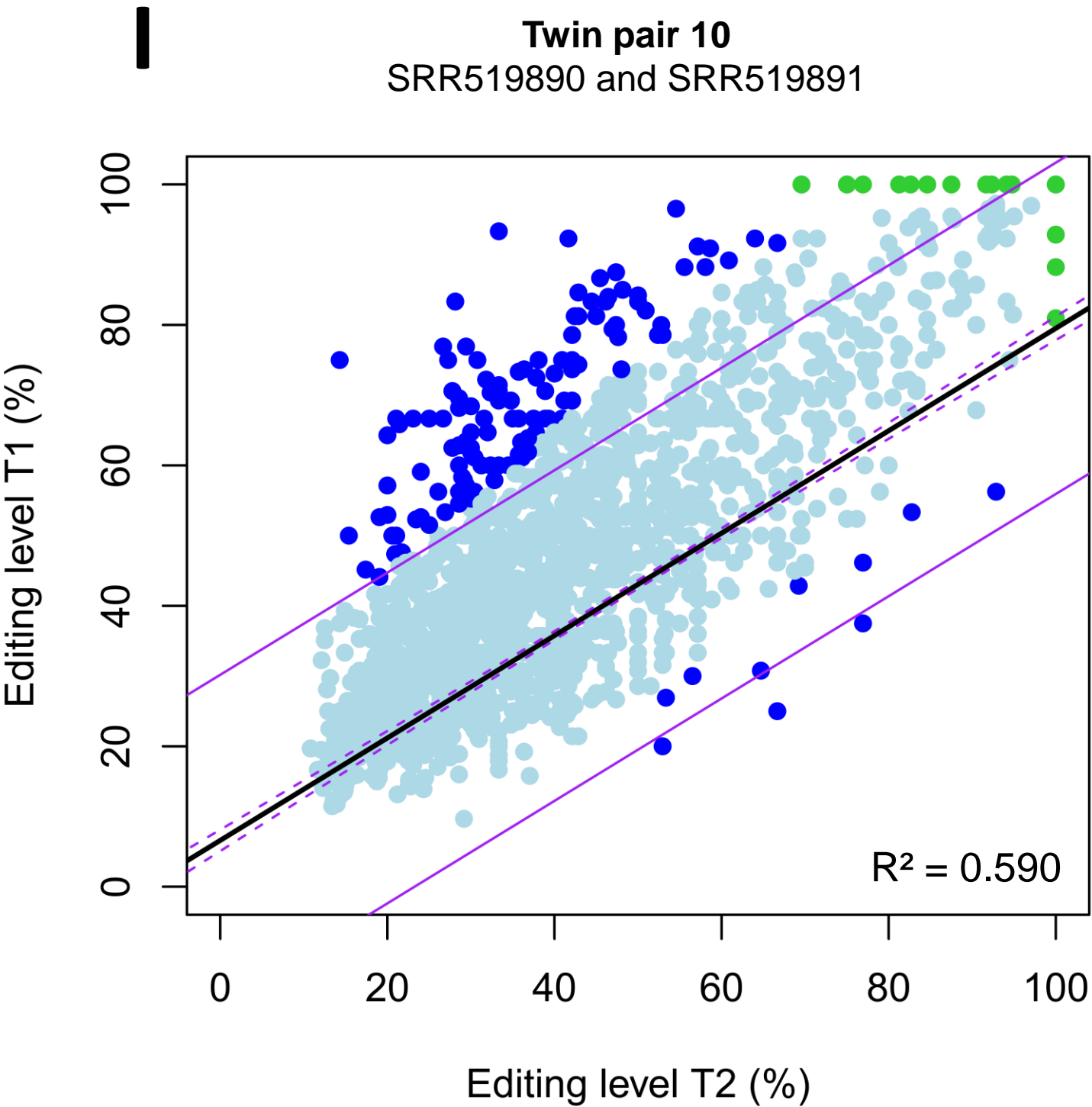

Supplement: Figure S7 — Within twin-pair disparities in allele expression proportions at eSNVs that are coincident with canonical A-to-I(G) RNA editing sites in homokaryotypic twins. Shown is the distribution of eSNVs that positionally match canonical RNA editing sites between nine homokaryotypic co-twins, (A) through (I), assayed in culture-B-cells. Each dot corresponds to an eSNV. The vast majority of sites exhibited a concordant biallelic imbalance profile (pink and light blue dots). Red dots represent eSNPs that were discordant between co-twins in that they exhibited allelic proportions differences higher than 25%, regardless of the discordance or concordance in the karyotype. Green dots represent eSNVs that exhibited discordant allelic profiles, being biallelic in one twin and monoallelic in the other. The linear models (solid black lines), the confidence interval of the models (broken purple lines) and of the prediction (solid purple lines) were constructed using R. Model equations: (A) Y = 8.15439 + 0.82362X; (B) Y = 7.20067 + 0.80502X; (C) Y = 6.0758 + 0.8080X; (D) Y = 12.74607 + 0.78267X; (E) Y = 14.78510 + 0.79019X; (F) Y = 5.64298 + 0.81255X; (G) Y = 7.97718 + 0.82152X; (H) Y = 8.36441 + 0.81231X; (I) Y = 6.60496 + 0.72912X. For all pairs, P < 2.2e-16. The RNA-Seq SRA entries for the nine twin pairs used as controls are SRR519874, SRR519875, SRR519876, SRR519877, SRR519878, SRR519879, SRR519880, SRR519881, SRR519882, SRR519883, SRR519884, SRR519885, SRR519886, SRR519887, SRR519888, SRR519889, SRR519890, and SRR519891. [file Image_7.pdf]
