# Supplementary material for: Pervasive Inter-Individual Variation in Allele-Specific Expression in Monozygotic Twins
Source: Front Genet. 2019 Nov 26;10:1178. doi: 10.3389/fgene.2019.01178 (PMC6887657; doi:10.3389/fgene.2019.01178)

Figure S4

Twin pair 02
SRR519874 and SRR519875

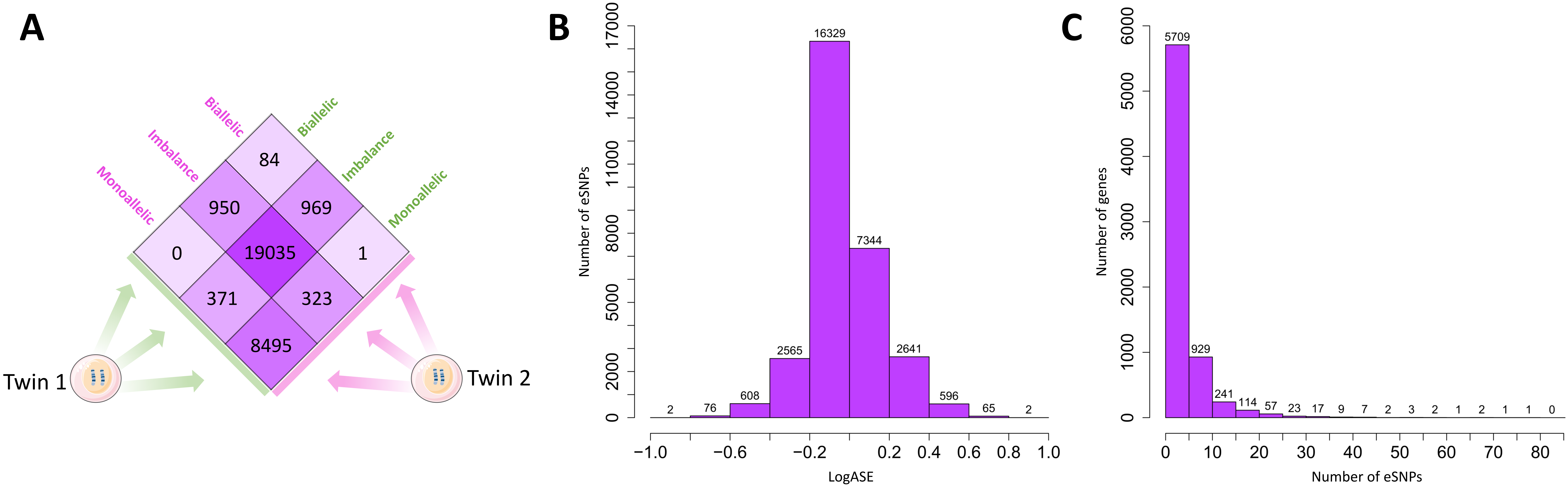

Twin pair 03
SRR519876 and SRR519877

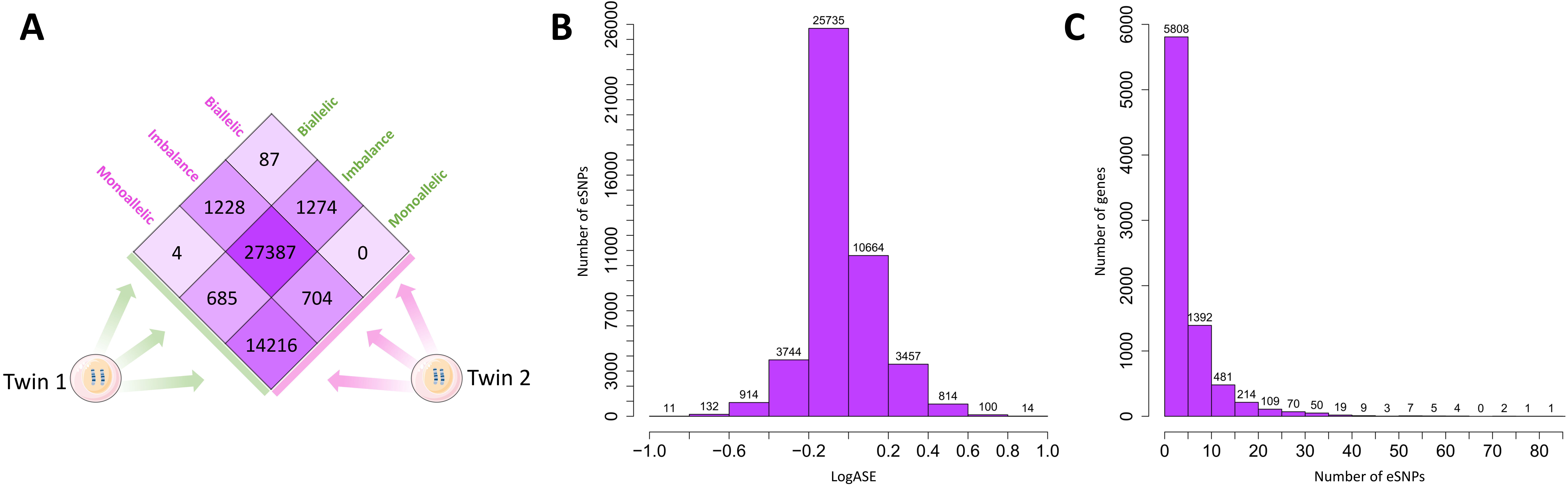

Twin pair 04

SRR519878 and SRR519879

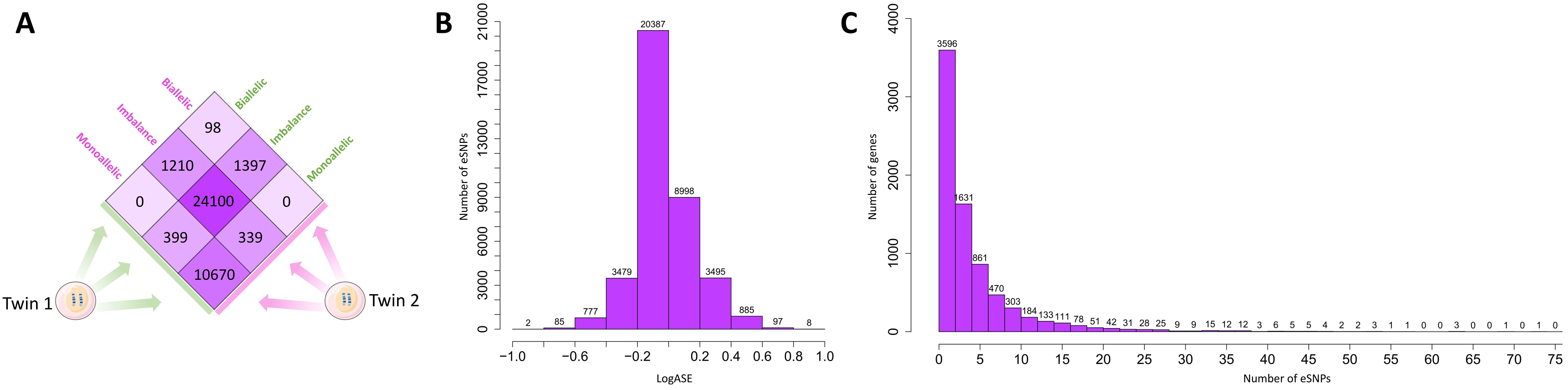

Twin pair 05
SRR519880 and SRR519881

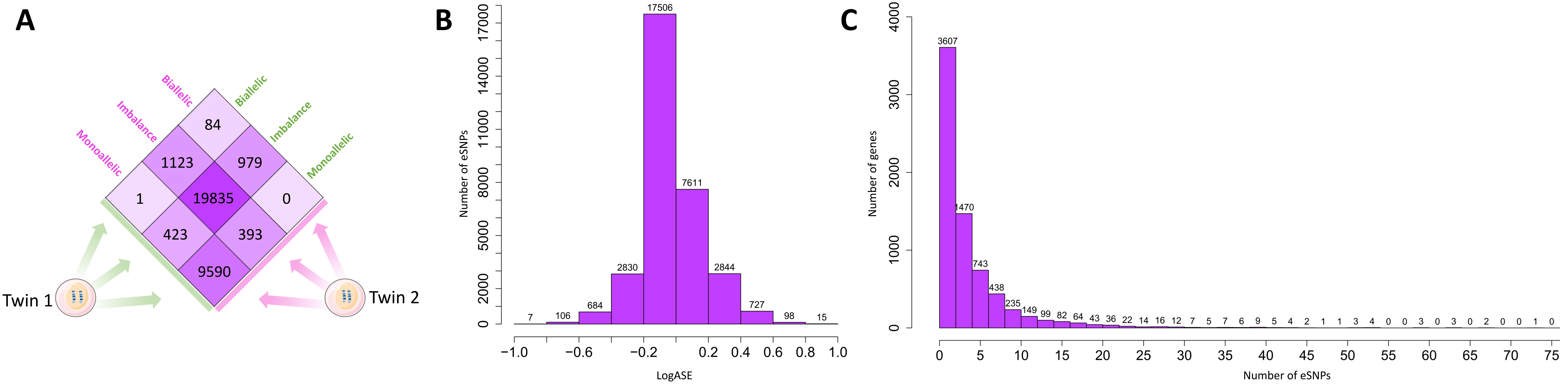

Twin pair 06
SRR519882 and SRR519883

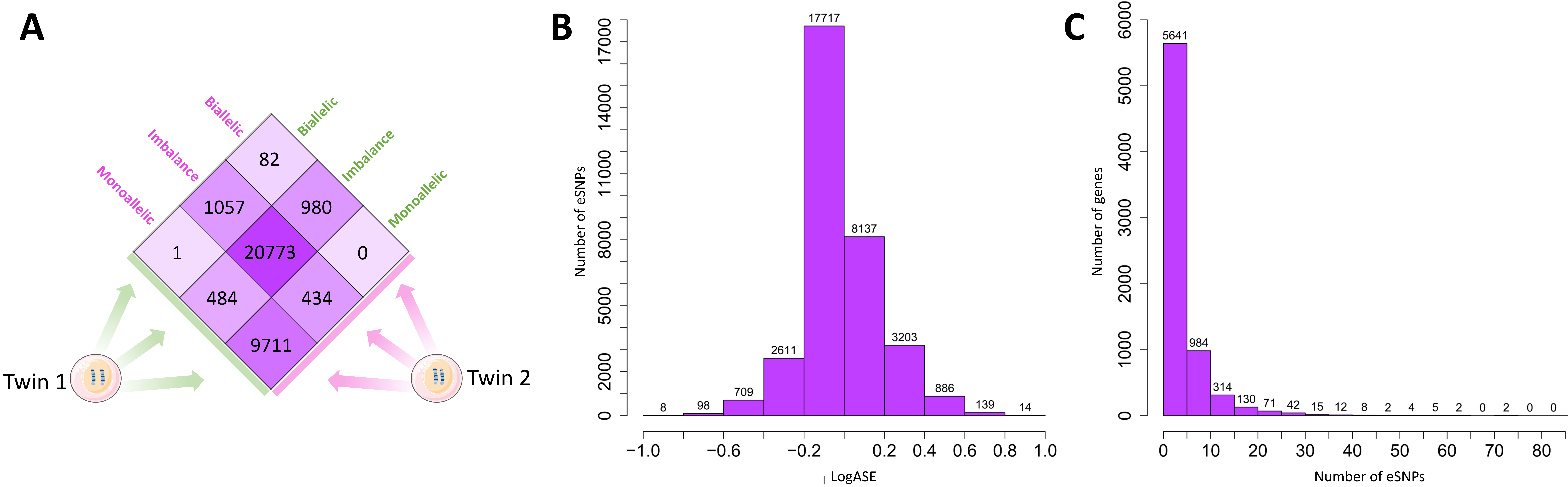

Twin pair 07
SRR519886 and SRR519887

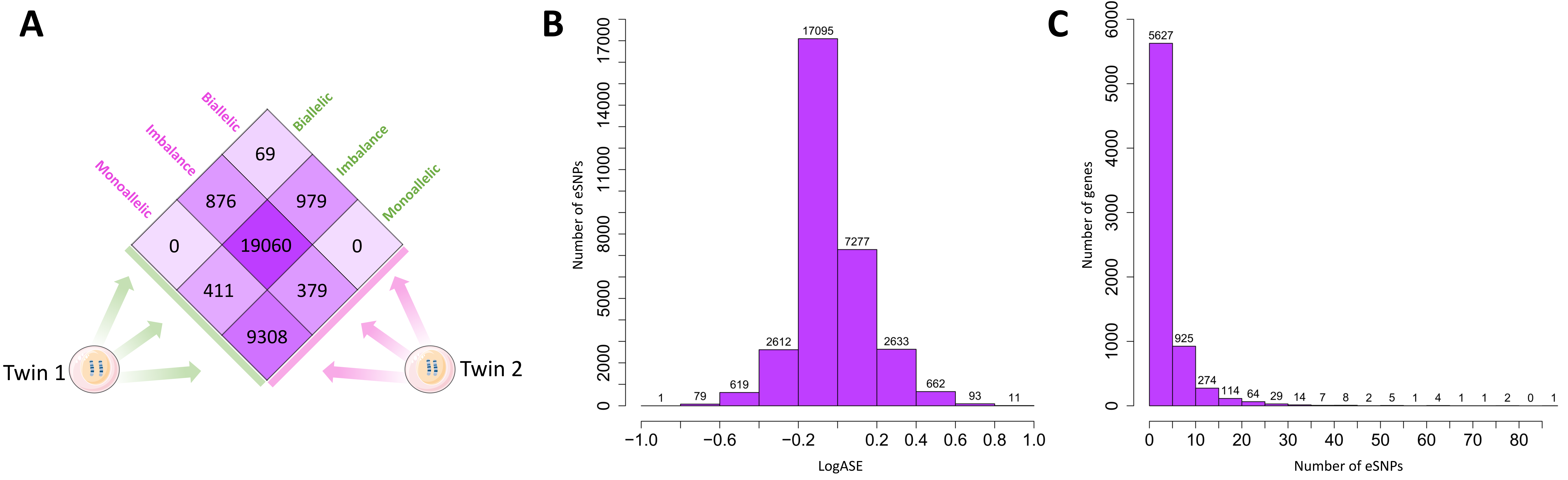

Twin pair 08
SRR519884 and SRR519885

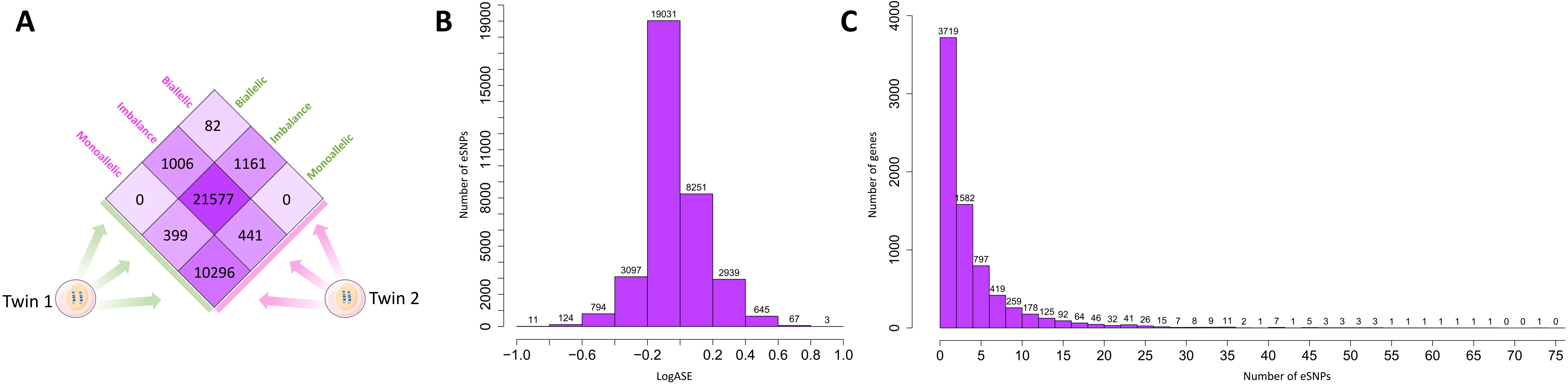

Twin pair 09

SRR519888 and SRR519889

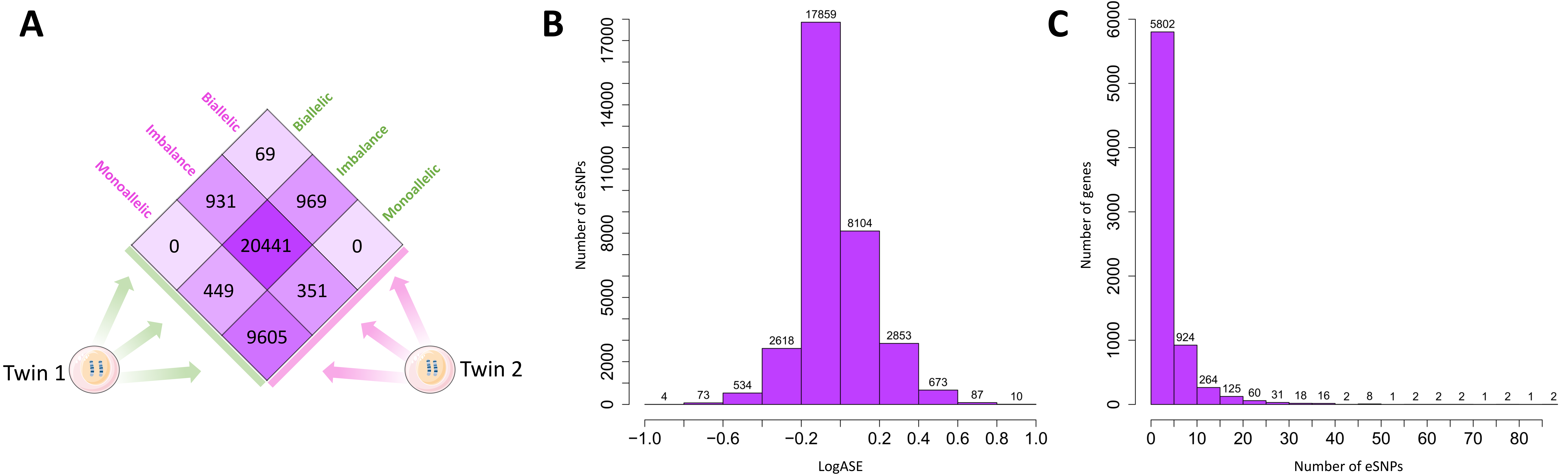

Twin pair 10
SRR519890 and SRR519891

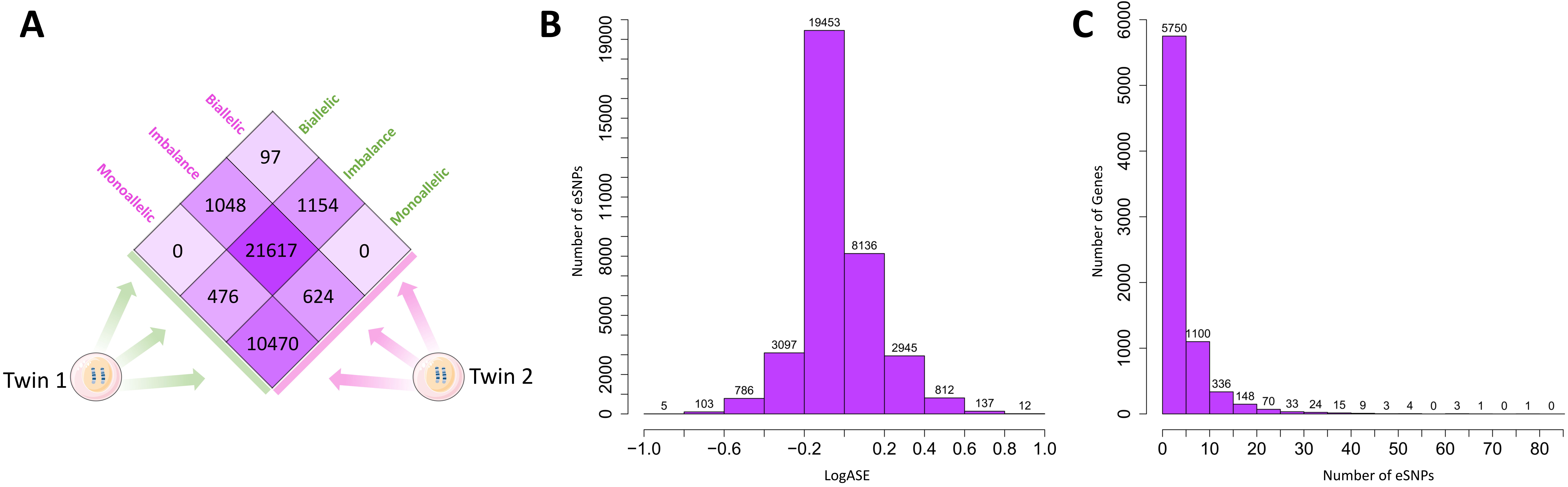

Supplement: Figure S4 — Overview of the breadth and magnitude of allele-specific expression disparity between nine MZ control twin pairs. The RNA-Seq SRA entries for the nine twin pairs are SRR519874, SRR519875, SRR519876, SRR519877, SRR519878, SRR519879, SRR519880, SRR519881, SRR519882, SRR519883, SRR519884, SRR519885, SRR519886, SRR519887, SRR519888, SRR519889, SRR519890, and SRR519891. For each SRA entry above, the panels are (A) numbers of ASE sites distributed by the within-pair status of concordance or discordance in control homokaryotypic MZ control twins tested in cultured B-cells. The majority of ASE sites are concordant for a biallelic imbalance status. On average, the co-twins are discordant in 1074 ± 252.03 ASE sites. (B) Comparison of the effect size of LogASE. We calculated the log2 of allele-specific expression fold change using the equation LogASE = log2(T1_ASE / T2_ASE) for each eSNV in each tissue. LogASE estimates the magnitude of expression change between conditions for the variant. (C) Distribution of genes by numbers of ASE sites observed in cultured B-cells. [file Image_4.pdf]
