# Supplementary material for: Pervasive Inter-Individual Variation in Allele-Specific Expression in Monozygotic Twins
Source: Front Genet. 2019 Nov 26;10:1178. doi: 10.3389/fgene.2019.01178 (PMC6887657; doi:10.3389/fgene.2019.01178)
Supplement: Figure S5 — Chromosomal distribution of eSNVs in the homokaryotypic twin pairs. Genome-wide e-karyotyping for the SNVs exhibiting allele-specific expression in cultured B-cells from nine control twin pairs. Shown in each panel,(A) through (I), is the distribution of all ASE sites that were concordant (gray ticks towards the left of each chromosome ideogram) or discordant (red ticks towards the right side). The RNA-Seq SRA entries for the nine twin pairs in are SRR519874, SRR519875, SRR519876, SRR519877, SRR519878, SRR519879, SRR519880, SRR519881, SRR519882, SRR519883, SRR519884, SRR519885, SRR519886, SRR519887, SRR519888, SRR519889, SRR519890, and SRR519891, respectively. [file Image_5.pdf]

Figure S5 (A)

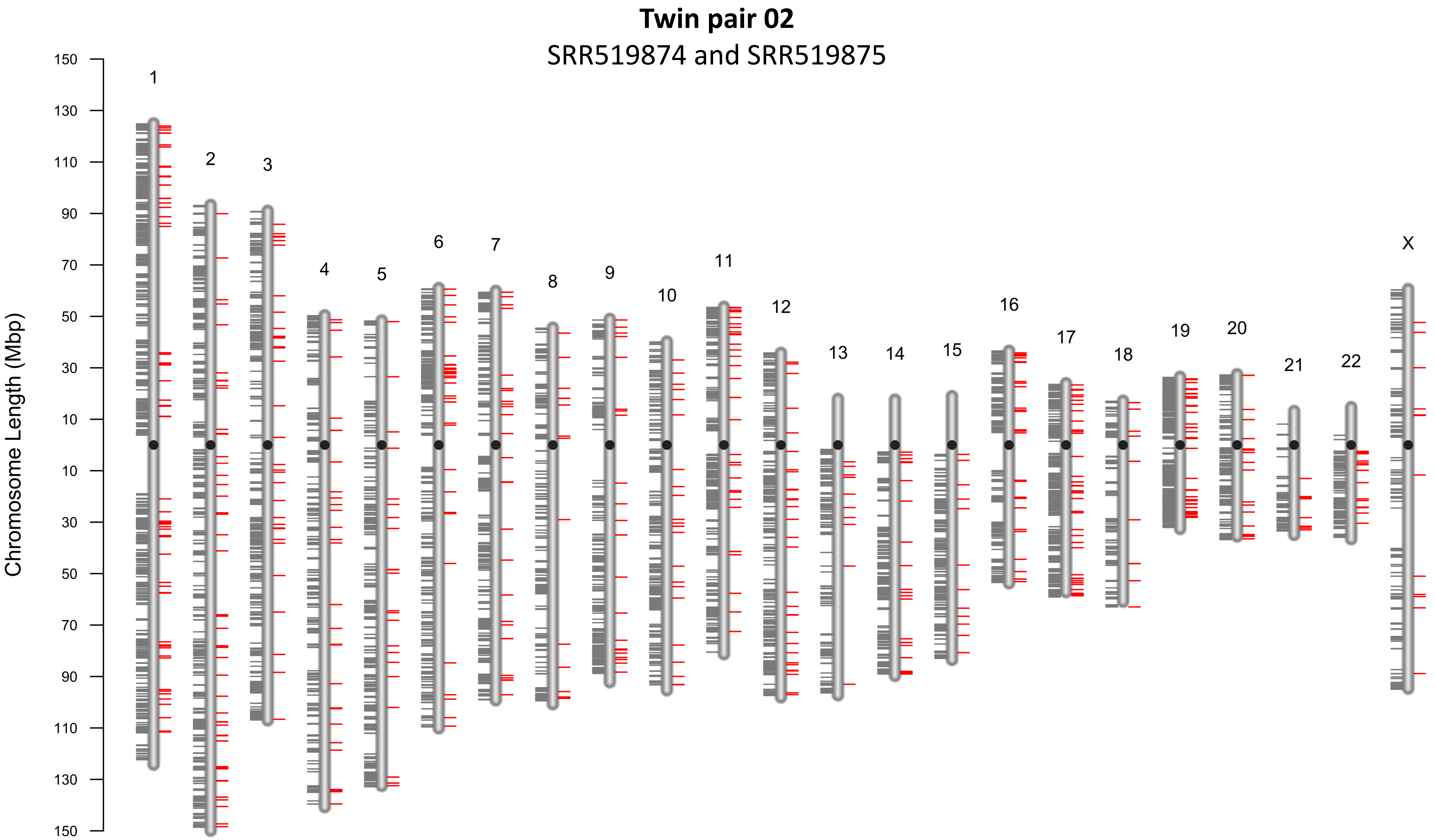
Twin pair 02
SRR519874 and SRR519875
Chromosome Length (Mbp)
150
130
110
90
70
50
30
10
10
30
50
70
90
110
130
150
1
2
3
4
5
6
7
8
9
10
11
12
13
14
15
16
17
18
19
20
21
22
X

Figure S5 (B)

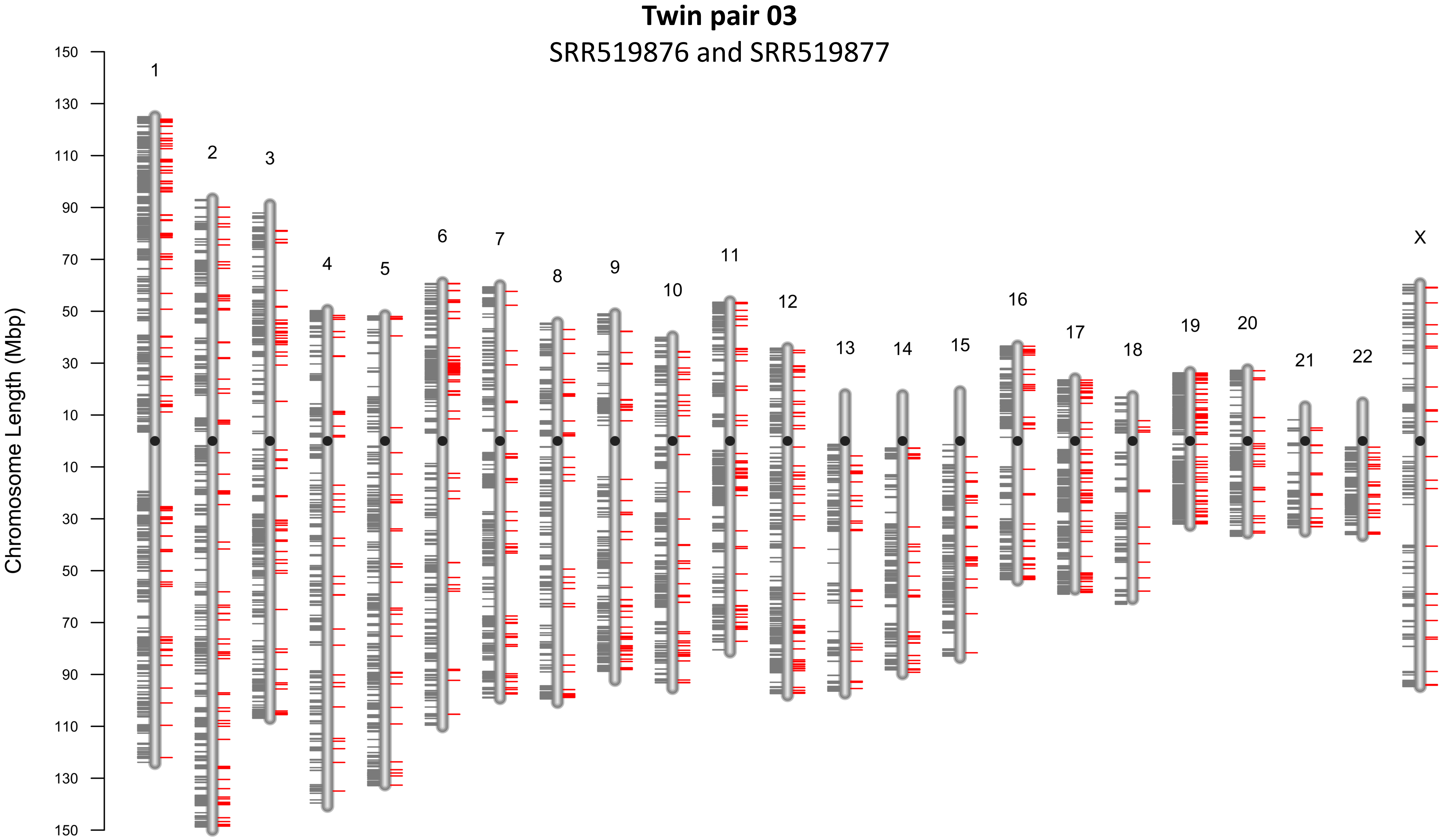

Twin pair 03
SRR519876 and SRR519877
Chromosome Length (Mbp)
150
130
110
90
70
50
30
10
10
30
50
70
90
110
130
150
1
2
3
4
5
6
7
8
9
10
11
12
13
14
15
16
17
18
19
20
21
22
X

Figure S5 (C)

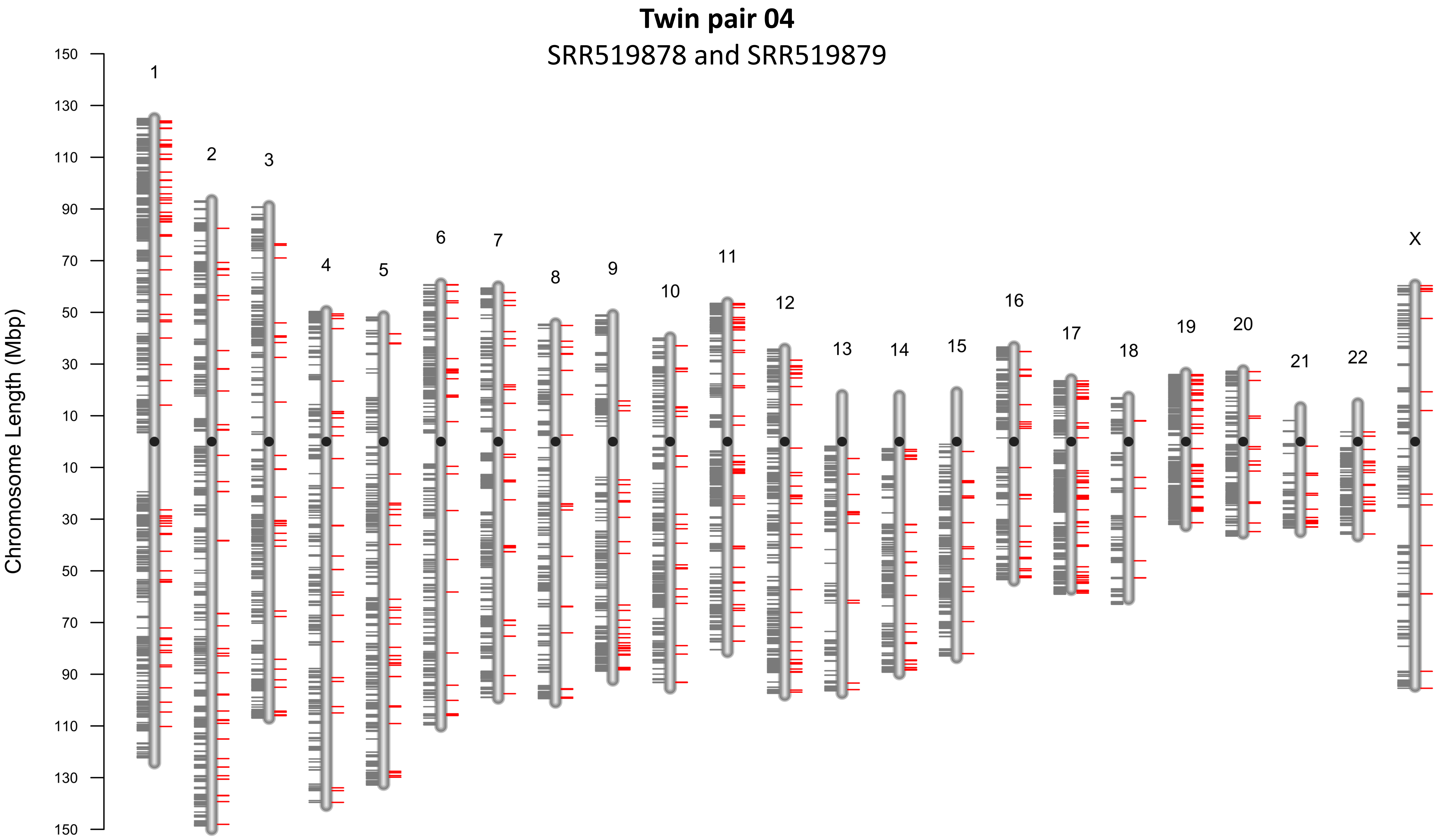

Twin pair 04
SRR519878 and SRR519879
Chromosome Length (Mbp)
150
130
110
90
70
50
30
10
10
30
50
70
90
110
130
150
1
2
3
4
5
6
7
8
9
10
11
12
13
14
15
16
17
18
19
20
21
22
X

Figure S5 (D)

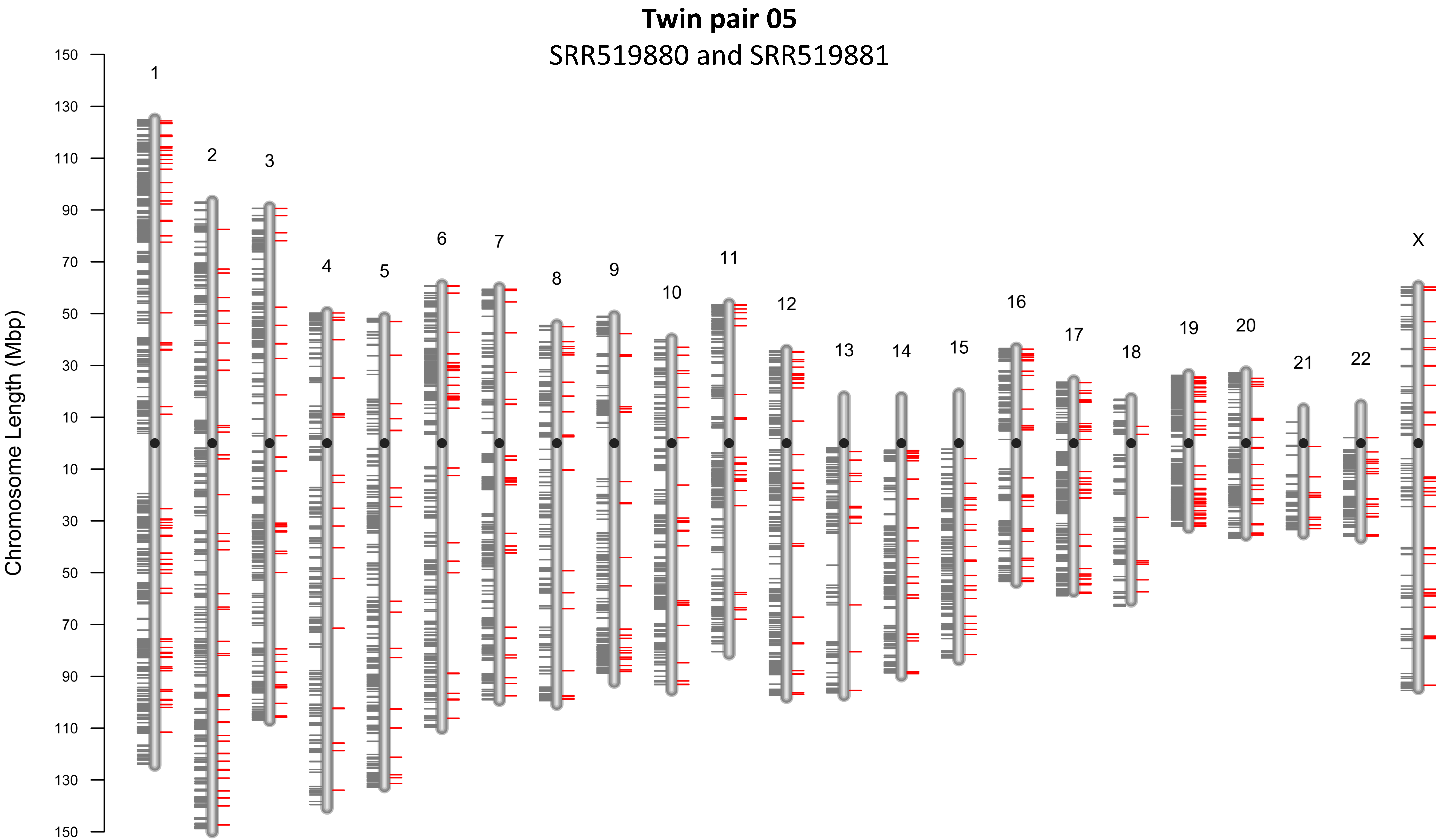

Twin pair 05
SRR519880 and SRR519881
Chromosome Length (Mbp)
150
130
110
90
70
50
30
10
10
30
50
70
90
110
130
150
1
2
3
4
5
6
7
8
9
10
11
12
13
14
15
16
17
18
19
20
21
22
X

Figure S5 (E)

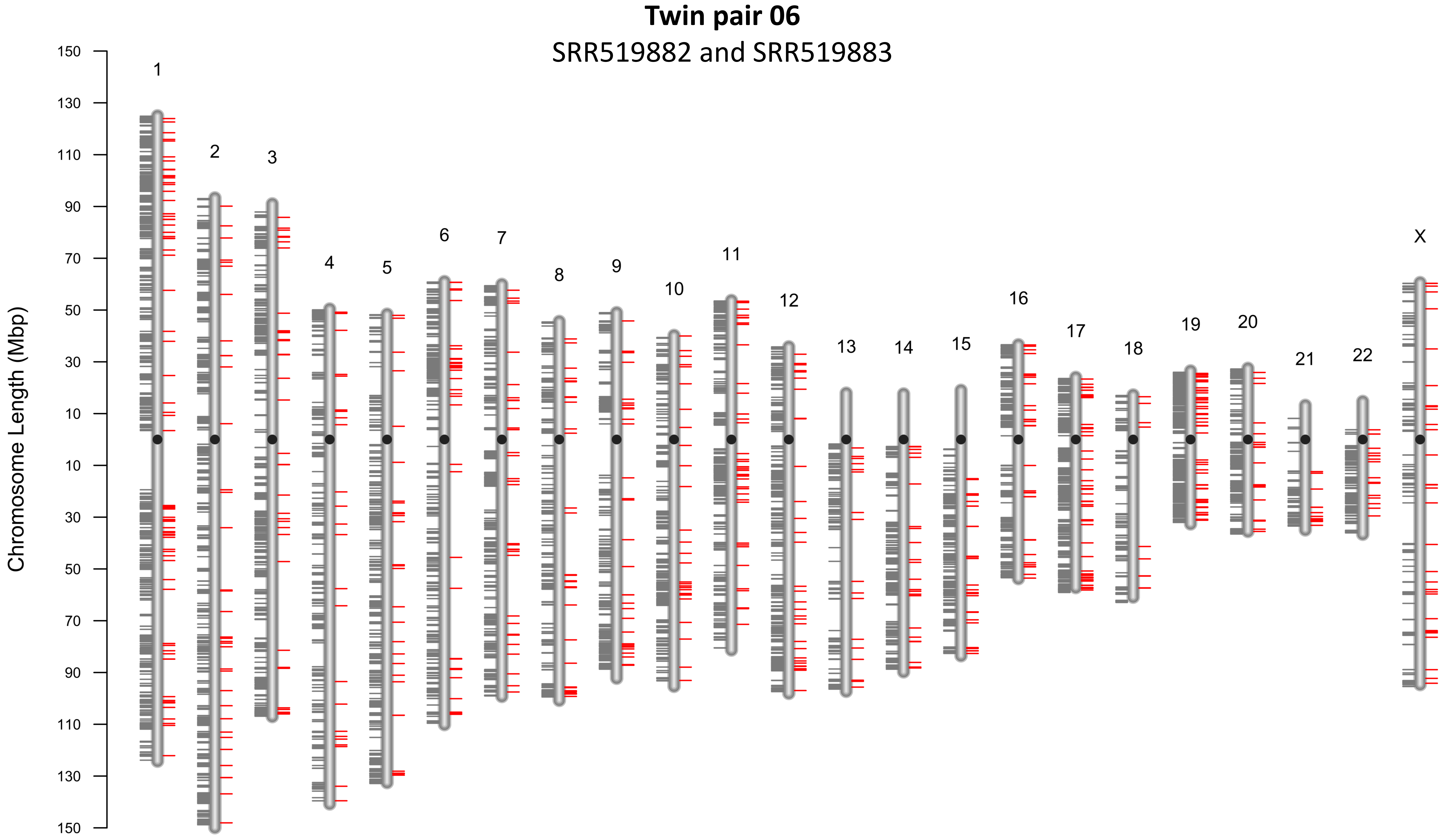

Twin pair 06
SRR519882 and SRR519883
Chromosome Length (Mbp)
150
130
110
90
70
50
30
10
10
30
50
70
90
110
130
150
1
2
3
4
5
6
7
8
9
10
11
12
13
14
15
16
17
18
19
20
21
22
X

Figure S5 (F)

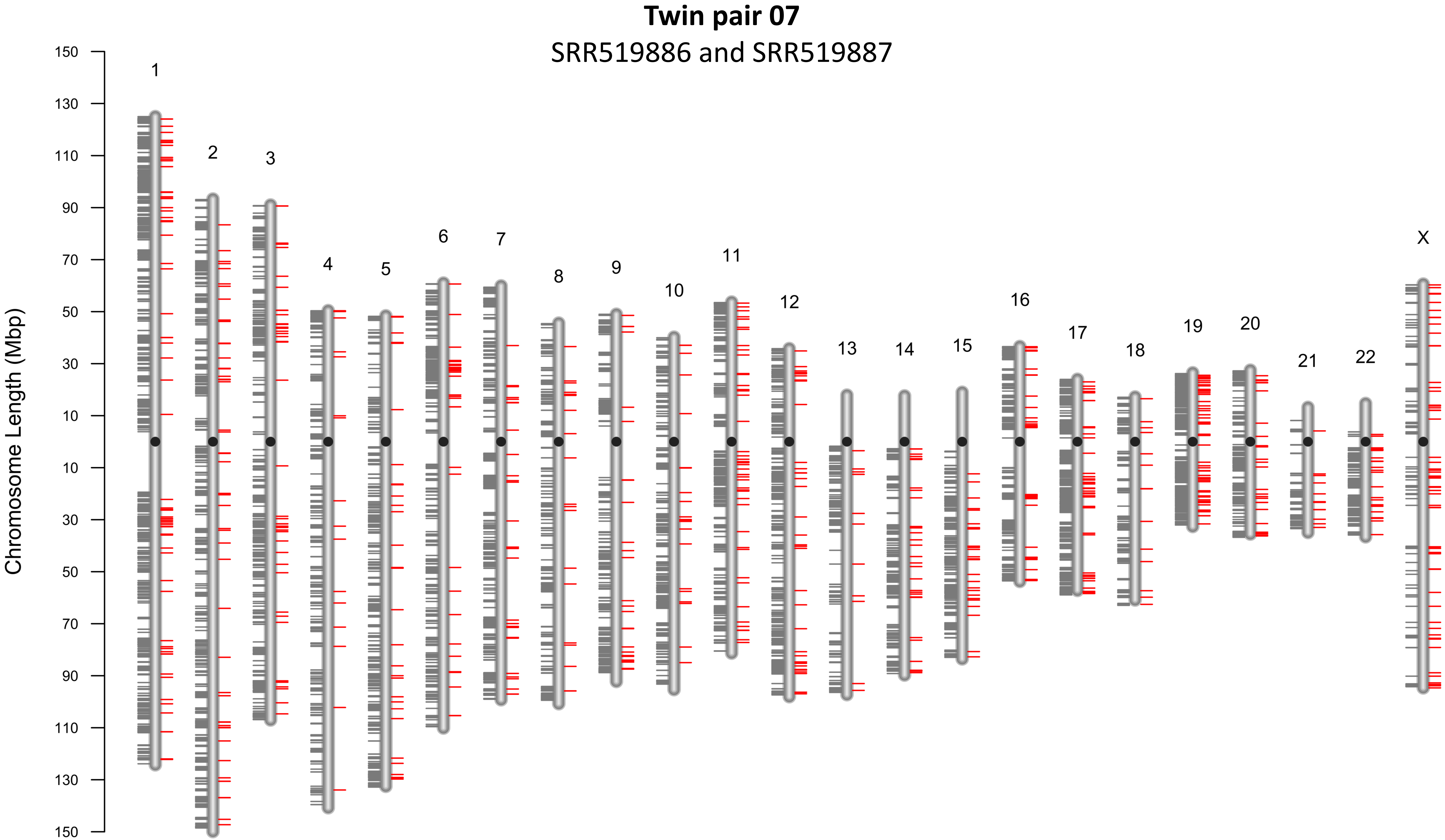

Twin pair 07
SRR519886 and SRR519887
Chromosome Length (Mbp)
150
130
110
90
70
50
30
10
10
30
50
70
90
110
130
150
1
2
3
4
5
6
7
8
9
10
11
12
13
14
15
16
17
18
19
20
21
22
X

Figure S5 (G)

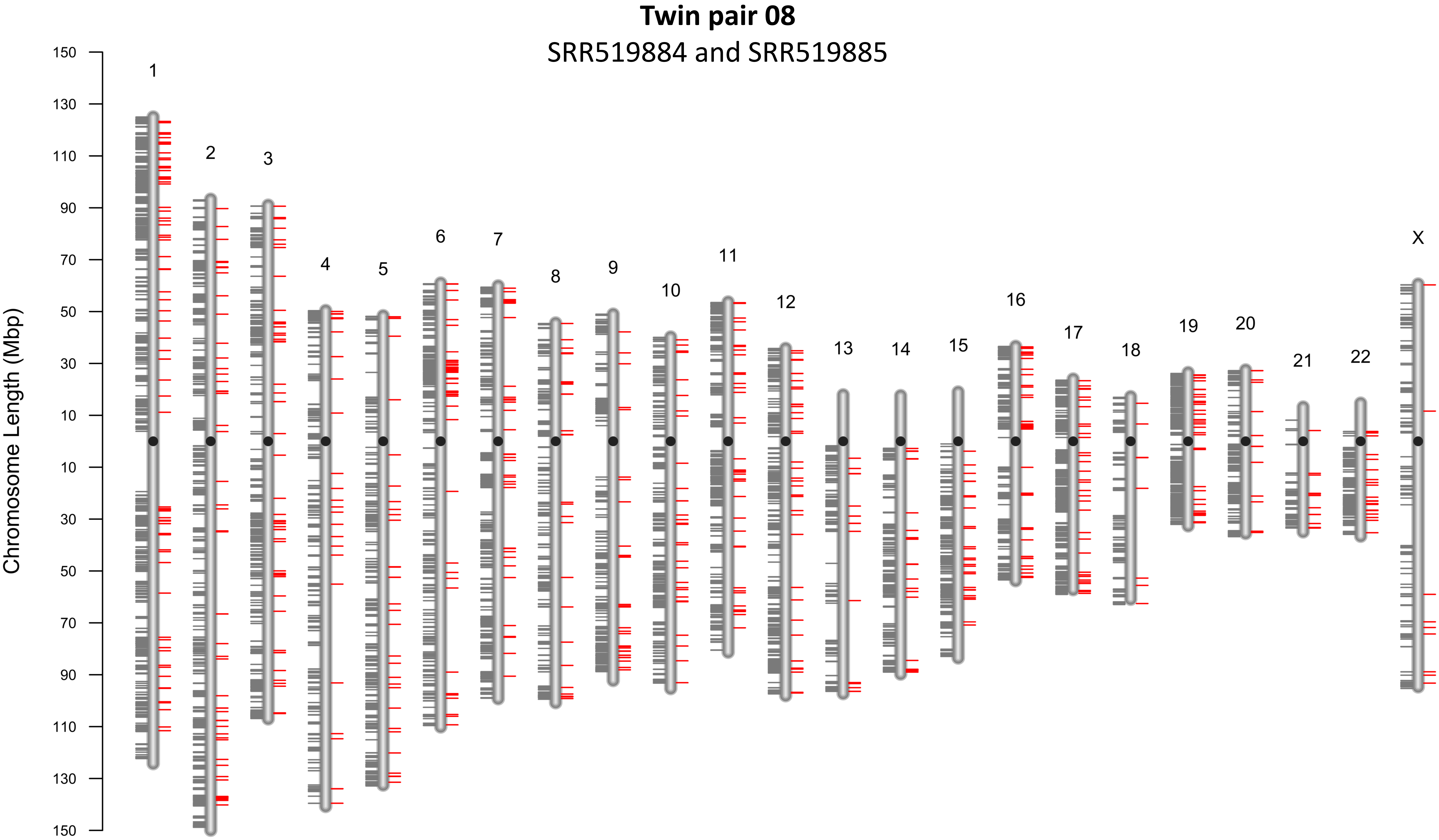

Twin pair 08
SRR519884 and SRR519885
Chromosome Length (Mbp)
150
130
110
90
70
50
30
10
10
30
50
70
90
110
130
150
1
2
3
4
5
6
7
8
9
10
11
12
13
14
15
16
17
18
19
20
21
22
X

Figure S5 (H)

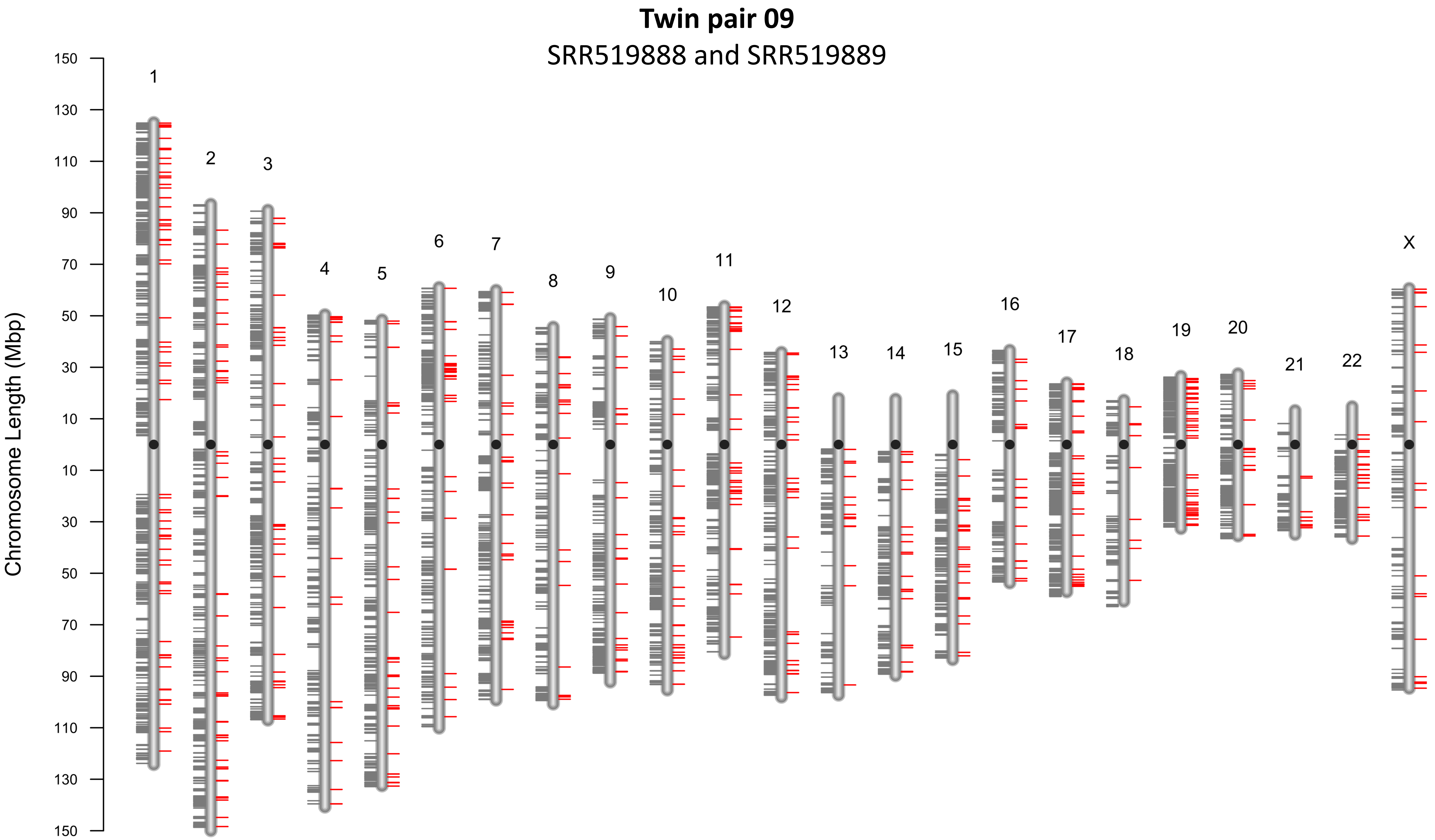
Twin pair 09
SRR519888 and SRR519889
Chromosome Length (Mbp)
150
130
110
90
70
50
30
10
10
30
50
70
90
110
130
150
1
2
3
4
5
6
7
8
9
10
11
12
13
14
15
16
17
18
19
20
21
22
X

Figure S5 (I)

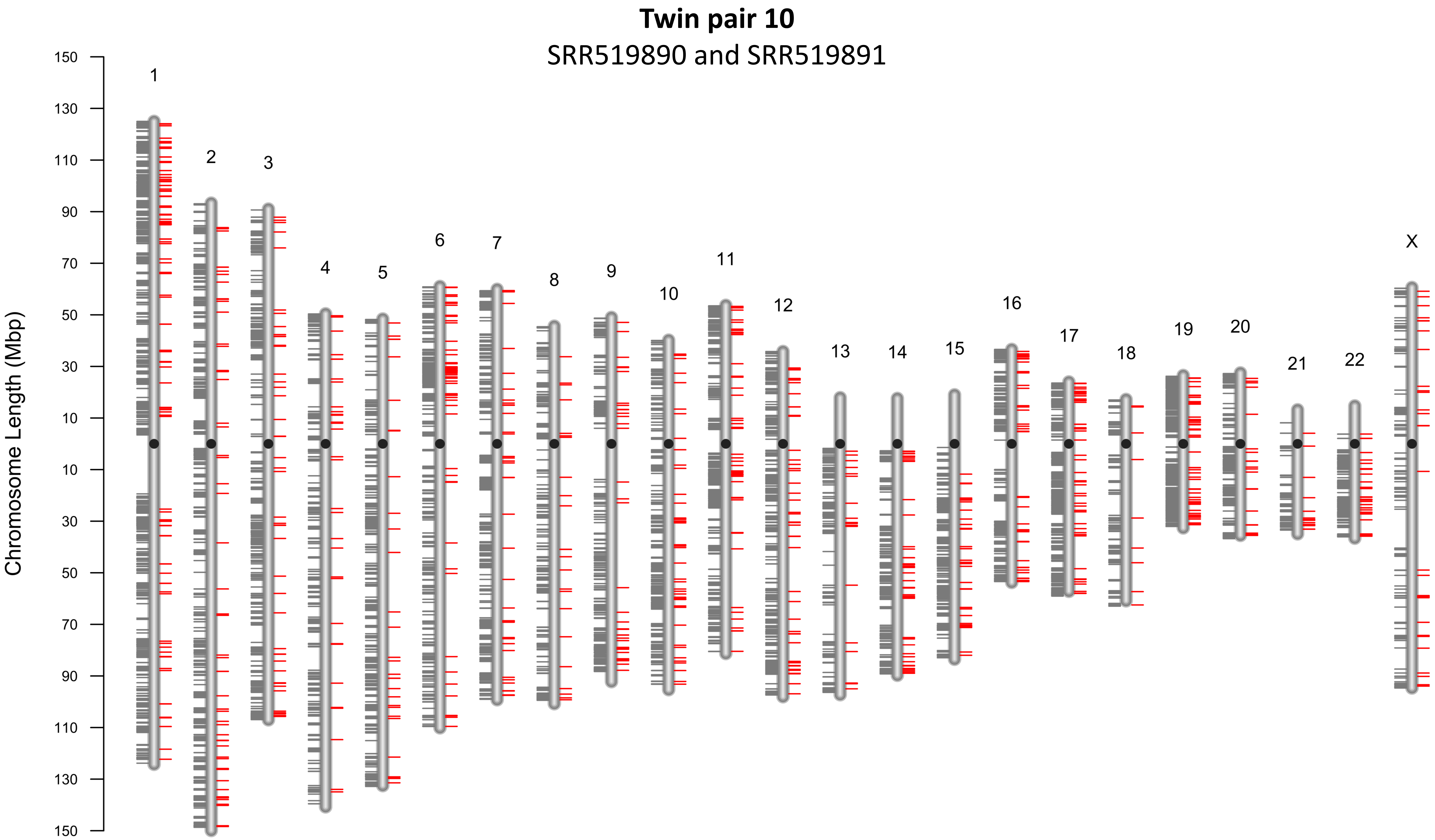

Twin pair 10
SRR519890 and SRR519891
Chromosome Length (Mbp)
150
130
110
90
70
50
30
10
10
30
50
70
90
110
130
150
1
2
3
4
5
6
7
8
9
10
11
12
13
14
15
16
17
18
19
20
21
22
X
