# Supplementary material for: Pervasive Inter-Individual Variation in Allele-Specific Expression in Monozygotic Twins
Source: Front Genet. 2019 Nov 26;10:1178. doi: 10.3389/fgene.2019.01178 (PMC6887657; doi:10.3389/fgene.2019.01178)
Supplement: Supplementary file 23 [file Presentation_1.pdf]

Pervasive Inter-individual Variation in Allele-Specific Expression in Monozygotic Twins

Ronaldo da Silva Francisco Junior; Cristina dos Santos Ferreira; Juan Carlo Santos e Silva; Douglas Terra Machado; Yasmmin Martins Cortes; Victor Ramos; Gustavo Simões Carnivali; Ana Beatriz Garcia; Enrique Medina-Acosta

SUPPLEMENTARY MATERIAL (Captions and legends)

Dataset S1. RNA-Seq read counts and allele ratios supporting Figure 1.

Dataset S2. BioProjects and RNA-Seq experiments used in this study.

Dataset S3. ASE concordant and discordant sites identified in heterokaryotypic MZ twins assayed in fibroblasts and induced pluripotent stem cells (iPSC) derived from the co-twins discordant for trisomy 21 and maternal recombination at 21q.

Dataset S4. Summary of the average distribution of eSNVs per gene in the entire set of biosamples and heterogeneity scores across genes. (A) Summary of informative of eSNVs, (B) Summary of eSNVs by ASE profile. Ranking of genes by ASE heterogeneity/homogeneity scores in either heterokaryotypic (C) and homokaryotypic (D) twins.

Dataset S5. Concordant and discordant ASE sites identified in unrelated, non-twin males (A) and female (B) pairs assayed in peripheral mononuclear cells. Ranking of genes by ASE heterogeneity/homogeneity scores in unrelated, non-twin males and females (C).

Datasets S6-S14. Concordant and discordant ASE sites identified in homokaryotypic MZ twins assayed in cultured B-cells derived from nine pairs of co-twins not discordant for a specific health condition.

Dataset S15. Cross-reference of ASE sites from the ten monozygotic twin pairs with allele expression profiles from secondary data of the GTEx project in multiple tissues.

Dataset S16. Summary of A-to-I(G) RNA-editing sites resulting in synonymous and non-synonymous substitutions in the ten twin pairs set.

Dataset S17. Protein-coding genetic eSNVs associated with disease risk or disease pathogenesis in the ten twin pairs set.

Dataset S18. Gene ontology enrichment annotations.

Table S1. **Review of cases of heterokaryotypic co-twins reported in the literature.**

Table S2. Distribution of ASE sites by gene structure sequence context. Cross-reference of ASE sites disparities observed in the present study with the annotation features of the eSNV disparities found in the whole-genome sequencing study by Huang et al. 2019.

Table S3. ASE disparities in X-linked genes subject to XCI between MZ twins.

Table S4. Evidence for allele-specific expression form of mitochondrial microheteroplasmy in MZ twin pairs.

Supplementary Figures

Figure S1. Representation of the discordant maternal 21q inheritance in the pair of co-twins heterokaryotypic for trisomy 21 reported by Dahoun et al. (**2008**). The MZ co-twins are discordant for trisomy 21 of maternal origin in twin 1 (T1DS) and maternal allelic disparity at 21qter likely carried by disomic twin 2 (T2N). The discordant inheritance of 21q is probably due to meiosis I subtelomeric recombination event likely occurring between the maternal chromosomes 21 within the 1.7Mb interval (hg38) delimited by the short tandem repeat marker D21S1445, where alleles were identical in both twins and the short tandem repeat marker D21S1611, where different alleles were inherited.

Figure S2. Flowchart of analysis. The in-house computational pipeline, PipASE, used for scanning and sorting out genome-wide, allele-specific differences between MZ co-twins.

Figure S3. Chromosomal distribution of eSNVs in iPSC. (**A**) Genome-wide e-karyotyping for the SNVs exhibiting allele-specific expression in iPSC derived from the co-twins discordant for T21 and maternal recombination at 21q. Shown is the distribution of all ASE sites that were concordant (gray ticks towards the left of each chromosome ideogram) or discordant (blue ticks towards the right side). (**B**) Assessment of chromosomal aberrations by e-karyotyping allelic bias using RNA-Seq data from iPSC in (**A**).

Figure S4. Overview of the breadth and magnitude of allele-specific expression disparity between nine MZ control twin pairs. The RNA-Seq SRA entries for the nine twin pairs are SRR519874, SRR519875, SRR519876, SRR519877, SRR519878, SRR519879, SRR519880, SRR519881, SRR519882, SRR519883, SRR519884, SRR519885, SRR519886, SRR519887, SRR519888, SRR519889, SRR519890, and SRR519891. For each SRA entry above, the panels are (**A**) numbers of ASE sites distributed by the within-pair status of concordance or discordance in control homokaryotypic MZ control twins tested in cultured B-cells. The majority of ASE sites are concordant for a biallelic imbalance status. On average, the co-twins are discordant in 1074 ± 252.03 ASE sites. (**B**) Comparison of the effect size of LogASE. We calculated the log2 of allele-specific expression fold change using the equation $LogASE = log2(T1\_ASE / T2\_ASE)$ for each eSNV in each tissue. LogASE estimates the magnitude of expression change between conditions for the variant. (**C**) Distribution of genes by numbers of ASE sites observed in cultured B-cells.

Figure S5. Chromosomal distribution of eSNVs in the homokaryotypic twin pairs. Genome-wide e-karyotyping for the SNVs exhibiting allele-specific expression in cultured B-cells from nine control twin pairs. Shown in each panel, (**A**) through (**I**), is the distribution of all ASE sites that were concordant (gray ticks towards the left of each chromosome ideogram) or discordant (red ticks towards the right side). The RNA-Seq SRA

entries for the nine twin pairs in are SRR519874, SRR519875, SRR519876, SRR519877, SRR519878, SRR519879, SRR519880, SRR519881, SRR519882, SRR519883, SRR519884, SRR519885, SRR519886, SRR519887, SRR519888, SRR519889, SRR519890, and SRR519891, respectively.

Figure S6. Assessment of chromosomal aberrations by e-karyotyping allelic bias using RNA-Seq data from control twin pairs. (**A**) through (**I**). The RNA-Seq SRA entries for the nine twin pairs used as controls are SRR519874, SRR519875, SRR519876, SRR519877, SRR519878, SRR519879, SRR519880, SRR519881, SRR519882, SRR519883, SRR519884, SRR519885, SRR519886, SRR519887, SRR519888, SRR519889, SRR519890, and SRR519891, respectively. For each SRA entry above, a plot is shown, which represents the distribution of allele ratios in cultured B-cells from nine pairs of co-twins who are not discordant for a specific health condition. None of the control twin pairs present detectable chromosomal aberrations.

Figure S7. Within twin-pair disparities in allele expression proportions at eSNVs that are coincident with canonical A-to-I(G) RNA editing sites in homokaryotypic twins. Shown is the distribution of eSNVs that positionally match canonical RNA editing sites between nine homokaryotypic co-twins, (**A**) through (**I**), assayed in culture-B-cells. Each dot corresponds to an eSNV. The vast majority of sites exhibited a concordant biallelic imbalance profile (pink and light blue dots). Red dots represent eSNPs that were discordant between co-twins in that they exhibited allelic proportions differences higher than 25%, regardless of the discordance or concordance in the karyotype. Green dots represent eSNVs that exhibited discordant allelic profiles, being biallelic in one twin and monoallelic in the other. The linear models (solid black lines), the confidence interval of the models (broken purple lines) and of the prediction (solid purple lines) were constructed using R. Model equations: (A) Y = 8.15439 + 0.82362X; (B) Y = 7.20067 + 0.80502X; (C) Y = 6.0758 + 0.8080X; (D) Y = 12.74607 + 0.78267X; (E) Y = 14.78510 + 0.79019X; (F) Y = 5.64298 + 0.81255X; (G) Y = 7.97718 + 0.82152X; (H) Y = 8.36441 + 0.81231X; (I) Y = 6.60496 + 0.72912X. For all pairs, $P < 2.2e\text{-}16$. The RNA-Seq SRA entries for the nine twin pairs used as controls are SRR519874, SRR519875, SRR519876, SRR519877, SRR519878,

SRR519879, SRR519880, SRR519881, SRR519882, SRR519883, SRR519884, SRR519885, SRR519886, SRR519887, SRR519888, SRR519889, SRR519890, and SRR519891.
